# Studies on the Lithiation, Borylation, and 1,2‐Metalate Rearrangement of *O*‐Cycloalkyl 2,4,6‐Triisopropylbenzoates

**DOI:** 10.1002/anie.202101374

**Published:** 2021-04-12

**Authors:** Rory C. Mykura, Pradip Songara, Eugenia Luc, Jack Rogers, Ellie Stammers, Varinder K. Aggarwal

**Affiliations:** ^1^ Department of Chemistry University of Bristol Cantock's Close Bristol BS8 1TS UK

**Keywords:** boronic esters, carbocycles, C−C coupling, cyclobutane, lithiated carbamates

## Abstract

A broad range of acyclic primary and secondary 2,4,6‐triisopropylbenzoate (TIB) esters have been used in lithiation‐borylation reactions, but cyclic TIB esters have not. We have studied the use of cyclic TIB esters in lithiation‐borylation reactions and looked at the effect of ring size (3‐ → 6‐membered rings) on the three key steps of the lithiation‐borylation protocol: deprotonation, borylation and 1,2‐metalate rearrangement. Although all rings sizes could be deprotonated, the cyclohexyl case was impractically slow, and the cyclopentyl example underwent α‐elimination faster than deprotonation at −78 °C and so could not be used. Both cyclobutyl and cyclopropyl cases underwent rapid borylation, but only the cyclobutyl substrate underwent 1,2‐metalate rearrangement. Thus, the cyclobutyl TIB ester occupies a “Goldilocks zone,” being small enough for deprotonation and large enough to enable 1,2‐migration. The generality of the reaction was explored with a broad range of boronic esters.

## Graphical abstract

A series of *O*‐cycloalkyl benzoates were studied to understand their ability to undergo a lithiation–borylation reaction. Ring size was the key feature: too large and the system could not be lithiated; too small and the system could not undergo 1,2‐migration. The cyclobutyl system occupies a “Goldilock's zone” where the lithiation–borylation reaction works well and shows broad substrate scope.

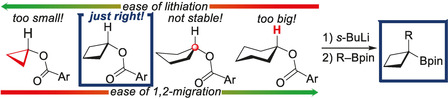

## Introduction

**Scheme 1:**
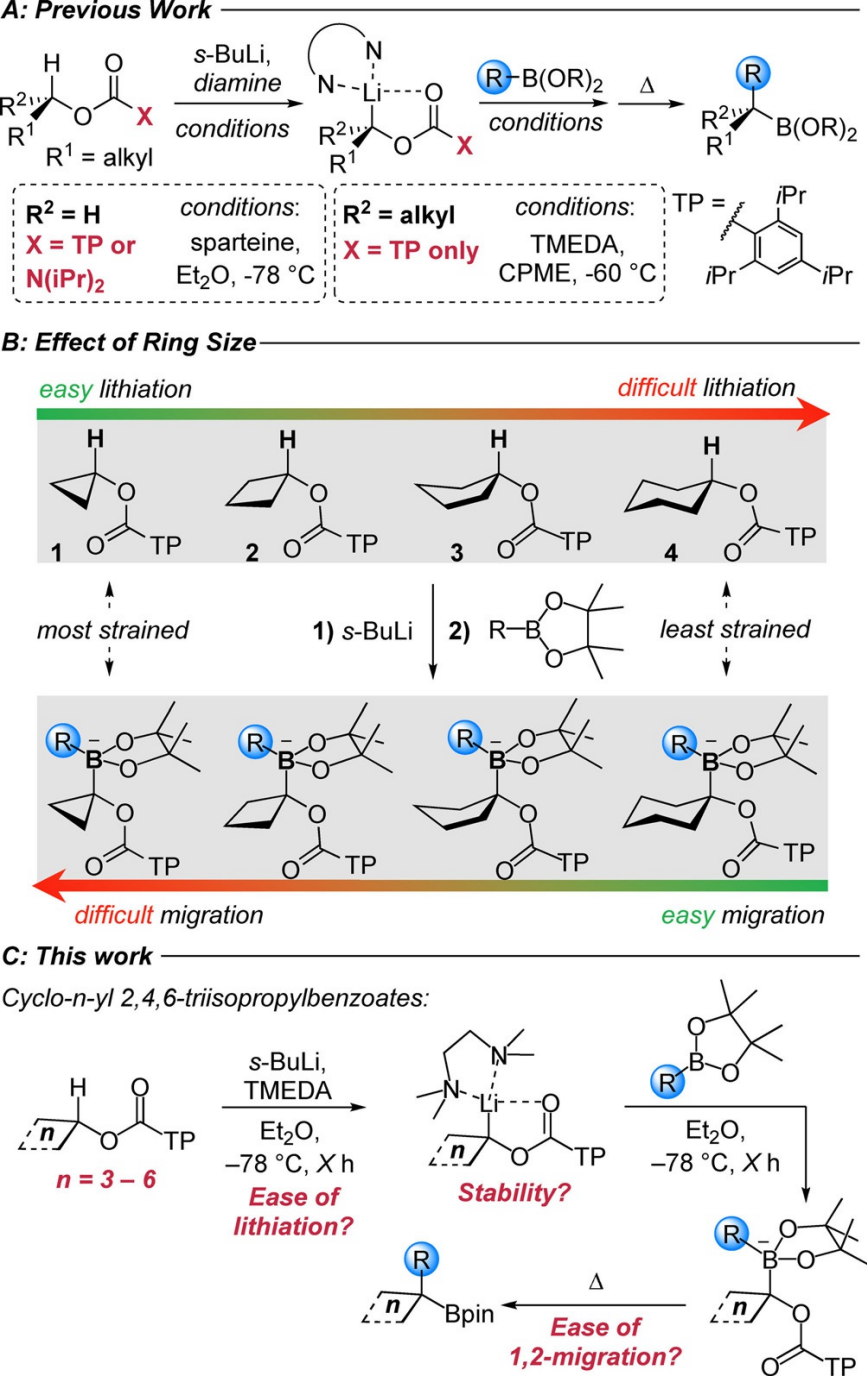
A) Current lithiation–borylation conditions with acyclic alkyl substrates. B) Factors affecting deprotonation and 1,2‐metalate rearrangement of cyclic TIB esters. C) This work: the effect of ring size on lithiation–borylation processes. TMEDA=*N*,*N*,*N*′,*N*′‐tetramethylethylenediamine, TIB=2,4,6‐i‐Pr_3_C_6_H_2_C(=O), TP=2,4,6‐i‐Pr_3_C_6_H_2_.

Boronic ester homologation represents a powerful method in asymmetric synthesis.^[1]^ Originally done through substrate control using a chiral diol on the boronic ester backbone^[2]^ it was later shown that reagent control offered greater flexibility and when used in an iterative manner was also more effective and more efficient. Both substituted chlorosulfoxides^[3]^ and hindered carbamates or benzoates^[4]^ have been investigated for reagent‐controlled homologation, the latter showing broader substrate scope. The hindered carbamates or benzoate esters (for example, *O*‐alkyl 2,4,6‐triisopropylbenzoate esters (TIB esters)^[5]^) are deprotonated by strong base in the presence of diamine ligands and trapped by boronic esters, leading to intermediate boronate complexes, which upon warming, undergo 1,2‐metalate rearrangement, forming homologated boronic esters (Scheme 1 A).^[6]^ The carbamate (or TIB ester) serves not only as a directing and stabilizing group for the lithiation step, but also as a leaving group in the 1,2‐metalate rearrangement step. The reaction is stereospecific, enabling homologated boronic esters to be produced with high enantioselectivity from enantioenriched lithiated carbamates/TIB esters. This lithiation–borylation protocol has been used extensively in the synthesis of natural[^1^, ^7^] and unnatural^[8]^ products. Both primary and secondary carbamate/TIB esters can be employed, but in the case of secondary substrates which do not have additional anion‐stabilizing groups (for example, aryl,^[9]^ allyl,^[10]^ or propargyl^[11]^) much more forcing conditions are required for deprotonation.^[12]^ In these cases TIB esters were found to facilitate deprotonation better than the corresponding carbamates. The lithiation–borylation methodology has been explored extensively on acyclic substrates but little work has been done on cyclic substrates that do not have additional anion‐stabilizing groups. Since carbocycles are ubiquitous in nature we were interested in exploring the scope of this methodology to cyclic variants bearing different ring sizes.

In traversing the series of cyclic alkanol TIB esters, deprotonation becomes progressively easier with decreasing ring size due to the increasing s‐character of the C−H bond (Scheme 1 B).^[13]^ In fact, there is only one report on the deprotonation of cyclic TIB esters, that of the cyclopropyl TIB ester, the most acidic in the series.^[14]^ Indeed, the high acidity of cyclopropanes enabled even bromocyclopropane to be deprotonated with lithium tetramethylpiperidine (LiTMP), and when (1‐bromocyclopropyl)lithium was generated in the presence of boronic esters, borylation and 1,2‐metalate rearrangement ensued.^[15]^ Numerous reports detail the lithiation of benzo fused 5‐ and 6‐membered systems however these benefit from stabilization by the adjacent phenyl ring.[^7b^, ^16^] For 1,2‐migration of the boronate complexes, the opposite trend is expected: it becomes progressively easier with increasing ring size due to the reduction in ring strain in the transition state (TS). In the case of the reaction of (1‐bromocyclopropyl)lithium with boronic esters, 1,2‐migration is enabled by having a very good leaving group. The competing trends in ease of lithiation with *decreasing* ring size and ease of 1,2‐migration with *increasing* ring size warranted a full investigation of lithiation‐borylation of cyclic substrates. We now report that of the 3–6‐membered rings investigated, only 4‐membered rings can be successfully employed. They occupy a “Goldilocks zone”, where small rings are required for deprotonation and large rings are required for 1,2‐migration (Scheme 1 b), although this study revealed a further important factor in determining success, the stability of the intermediate lithiated TIB ester towards α‐elimination. This work is not only of fundamental interest, but it is also of practical utility since the success with 4‐membered rings leads to cyclobutane products which are useful and highly sought‐after, particularly in medicinal chemistry as they provide a defined spatial arrangement of groups due to their rigid scaffolds.^[17]^

## Results and Discussion

**Scheme 2:**
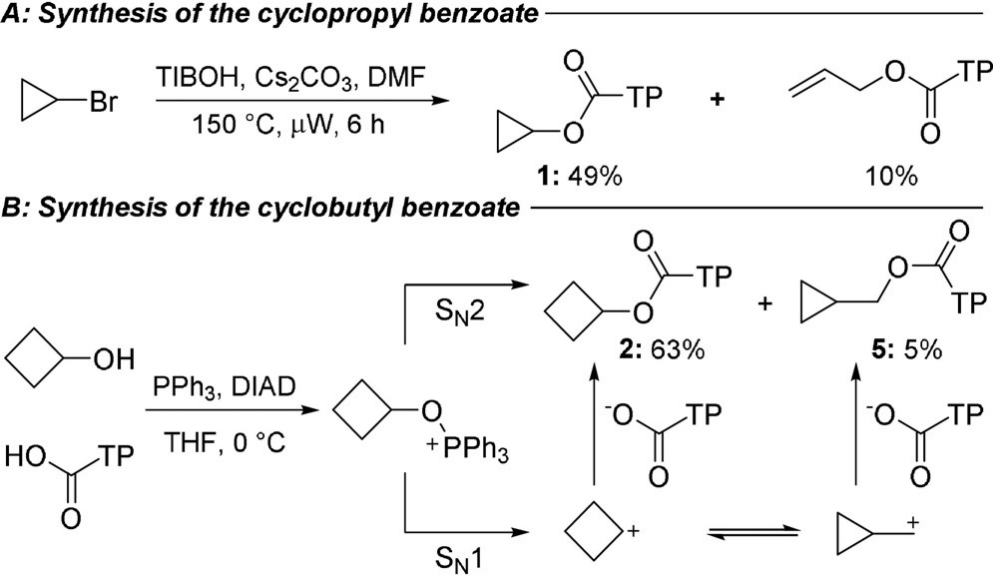
Synthesis of small ring benzoates and side‐products observed.

Our investigation began with the synthesis of the cyclic TIB esters **1**–**4**. After some experimentation we found that the cyclopropyl ester could be made by S_N_2 displacement of the corresponding bromide with 2,4,6‐triisopropylbenzoic acid (TIB acid) (Scheme 2), whilst the 4–6‐membered ring TIB esters were best made by Mitsunobu reaction of the corresponding alcohols. Cyclopentanol and cyclohexanol proceeded smoothly (see Supporting Information for details), however in the case of the cyclobutanol, a small amount (5 %) of cyclopropylmethyl TIB ester **5** was obtained. This was presumably formed from rearrangement of a cyclobutyl carbocation, generated by an S_N_1 pathway, to a cyclopropylmethyl carbocation which was then trapped by the carboxylic acid (Scheme 2).^[18]^ This side product was not separable by chromatography, but fortunately the minor component did not interfere with the subsequent chemistry.

**Scheme 3:**
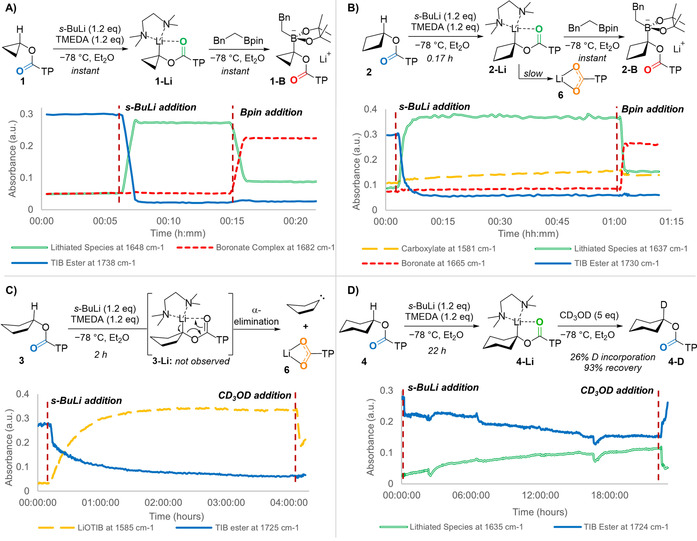
In situ IR spectroscopy traces for 3–6‐membered cycloalkyl TIB esters. A) Lithiation and borylation of the cyclopropyl TIB ester. The trace shows that the deprotonation is very rapid, the lithiated species is stable at −78 °C, and that borylation is rapid. B) Lithiation and borylation of the cyclobutyl TIB ester. The trace shows that deprotonation is rapid, the lithiated species slowly decomposes at −78 °C, and that borylation is rapid. C) Lithiation of the cyclopentyl TIB ester. The trace shows that the lithiated species is not stable and decomposes to the carboxylate. D) Partial lithiation and deuteration of the cyclohexyl TIB ester. The trace shows that the lithiation is slow and inefficient.

There are three steps associated with lithiation‐borylation reactions: 1) deprotonation to form the organolithium; 2) borylation to form the boronate complex; and 3) 1,2‐metalate rearrangement. In situ IR spectroscopy was used to optimise the first two steps, lithiation and borylation, as it allows determination of reaction times and desired stoichiometry in a single experiment, where the signal intensity of the carbonyl group is followed for each intermediate.^[19]^ In all examples, a solution of TIB ester **1**–**4** (0.3 m, Et_2_O) and *N*,*N*,*N*′,*N*′‐tetramethylethylenediamine (TMEDA, 1.2 equiv) was cooled to −78 °C and then *s*‐BuLi was added (1.3 m in cyclohexane, 1.2 equiv). Studying the most acidic cyclopropyl variant first (Scheme 3 A), upon addition of base a rapid decrease in the intensity of the signal attributed to the starting TIB ester (≈1730 cm^−1^) was observed. At the same time, a signal at a lower wavenumber 1648 cm^−1^ grew in intensity, which we attributed to the lithiated species **1‐Li**, which then plateaued and remained horizontal over 10 minutes showing that the lithiated cyclopropyl TIB ester was chemically stable under the reaction conditions. The lithiation was essentially instantaneous, being complete by the end of the dropwise addition of the base. A solution of phenethylboronic acid pinacol ester (1.0 m, Et_2_O) was then added over 2 minutes and another peak appeared (1682 cm^−1^), again instantaneously, which is indicative of the boronate complex **1‐B**. Even though the organolithium is tertiary (albeit bearing a small cyclopropyl group) rapid borylation ensued.

The cyclobutyl TIB ester **2** was then subjected to the same sequence (Scheme 3 B). In this case deprotonation was no longer instantaneous but it was still rapid, taking 10 minutes to reach a plateau. In contrast to the cyclopropyl example, the lithiated cyclobutyl substrate **2‐Li** was not chemically stable under the reaction conditions and decayed slowly over time. A broader second peak appeared at lower wavenumber 1581 cm^−1^, which is attributed to the carboxylate salt **6**, and was observed to grow after ≈50 minutes indicating slow decomposition by α‐elimination. Addition of phenethyl boronic acid pinacol ester then led to instantaneous borylation, like the cyclopropyl example, affording the cyclobutyl boronate complex **2‐B**. As the deprotonation of the cyclobutyl TIB ester was especially facile, we also attempted the lithiation‐borylation of bromo‐ and chlorocyclobutane, in a similar way to that employed for bromocyclopropane. However, we only observed trace product, presumably due to the enhanced rate of α‐elimination from having a better leaving group (see Supporting Information for details).

**Scheme 4:**
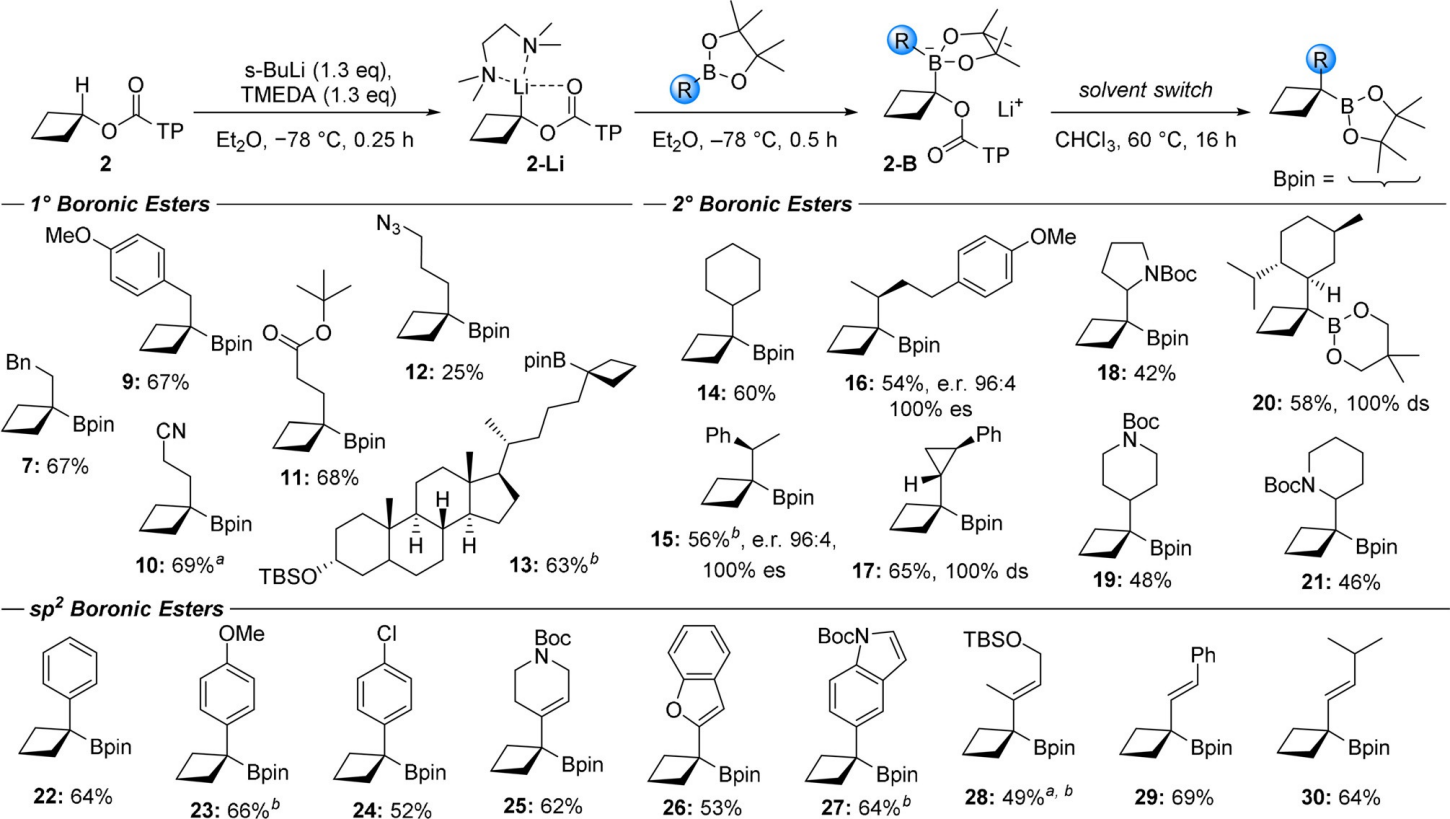
Substrate scope and boronic ester functionalisations. [a] Isolated with minor impurities. [b] Boronic ester was oxidized to the alcohol (H_2_O_2_, NaOH, 0 °C to 22 °C, 16 h) to aid purification owing to similar *R*_f_ values of reaction components (all boronic esters were stable to flash column chromatography).

Increasing the ring size further to the cyclopentyl TIB ester **3** led to unexpected results. While the starting material was converted more slowly than the smaller ring systems (as expected), there was no observable lithiated species **3‐Li**. Instead, only the broad peak of the carboxylate **6** was observed (Scheme 3 C). This indicates that the lithiated species undergoes decomposition (presumably by α‐elimination) at a faster rate than its formation. This was confirmed by addition of *d_4_*‐methanol (5 equiv) 4 h after addition of the base (when the formation of carboxylate had stopped by in situ IR spectroscopy), which led to a low recovery (27 %) of non‐deuterated (0 % deuterium incorporation) starting material. Attempts to stabilize the cyclopentyl lithiated species using the diisopropyl carbamate (a better stabilizing group and worse leaving group) were unsuccessful and no lithiated species was observed by in situ IR spectroscopy (see Supporting Information for details). We were surprised at the high instability of the lithiated cyclopentyl TIB ester and carbamate. Finally, the cyclohexyl TIB ester **4** showed much slower deprotonation still. After 22 h at −78 °C the reaction reached a plateau, with the in situ IR spectroscopy trace showing low conversion (Scheme 3 D). Quenching the reaction at this time with *d_4_*‐methanol gave 93 % recovery with only 26 % deuteration which is clearly impractical. Being able to observe the lithiated benzoate 4‐Li by in situ IR spectroscopy and the fact that we isolated **4‐D** showed that the lithiated benzoate **4‐Li** was much more stable than the 5‐membered ring analogue **3‐Li**. At −60 °C the deprotonation plateaued after 4 h and quenching with *d_4_‐*methanol gave similar levels of recovery (95 %) and deuterium incorporation (21 %) as at −78 °C. We have previously shown that *s*‐butyl TIB ester gave 35 % yield in a lithiation–borylation process^[12]^ at −60 °C after a 2 h lithiation time, indicating that cyclohexyl TIB ester is deprotonated even more slowly than acyclic substrates. Having established that both the cyclopropyl and cyclobutyl TIB esters could be lithiated and borylated, our attention turned to the 1,2‐metalate rearrangement. Beginning with the cyclopropyl boronate complex **1‐B** we attempted to promote 1,2‐metalate rearrangement but even under a variety of conditions (e.g. MgBr_2_, solvent swap to CHCl_3_), all reactions either returned starting material or resulted in decomposition (see Supporting Information for details). This clearly indicated that the barrier to 1,2‐migration for the 3‐membered ring was higher than alternative decomposition pathways or reversion to the starting components. Interestingly, cyclopropyl boronate complexes bearing a bromide leaving group do undergo 1,2‐metalate rearrangement,^[15]^ highlighting the difference the nature of the leaving group can make. Turning to the cyclobutyl boronate complex **2‐B**, we found that this time the 1,2‐metalate rearrangement began to occur at room temperature, but the reaction was slow. Even with heating in Et_2_O, boronate complex remained (Table 1, entries 1 and 2). Use of MgBr_2_ was not effective at promoting the 1,2‐metalate rearrangement (entry 3), but we found that a solvent switch to CHCl_3_ followed by heating to 60 °C enabled complete 1,2‐metalate rearrangement to occur in just 3 h, furnishing the cyclobutylboronic ester **7** in 67 % isolated yield. Solvent exchange to a non‐coordinating solvent, like CHCl_3_, has previously been found to promote 1,2‐migration of recalcitrant boronate complexes.^[20]^ A small amount of *O*‐migration of the pinacol group was also observed for all entries (**8**: <10 %). Having developed a successful lithiation‐borylation protocol for the cyclobutyl TIB ester (entry 4), we explored the scope of this process with different boronic esters (Scheme 4).

**Table 1:** Optimization of 1,2‐migration. 
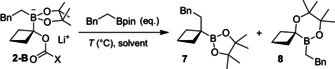

Entry	X	Equiv	Solvent	*T* [°C], *t* [h]	**8**:**7**:**2‐B** ^[a]^	Yield [%]
1	TP	1.2	Et_2_O	22, 16	10:70:20	64^[b]^ (59^[d]^)
2	TP	1.2	Et_2_O	30, 16	10:70:20	67^[b]^ (53^[d]^)
3	TP	1.5	Et_2_O	40, 16	15:65:25	42^[d,c]^
4	TP	1.5	CHCl_3_	60, 3	10:90:0	86^[b]^ (67^[d]^)
5	N(*i*Pr)_2_	1.5	CHCl_3_	60, 16	10:70:20	57^[b]^

[a] The ratio of *O*‐migrated side‐product (*δ*≈50 ppm) to *C*‐migrated product (*δ*≈32 ppm) to boronate complex (*δ*≈8 ppm), ratio determined by ^11^B NMR spectroscopy. [b] Crude ^1^H NMR yield relative to 1,3,5‐trimethoxybenzene. [c] 8 equivalents of a 1 m solution of MgBr_2_ in MeOH were added to the reaction. [d] Isolated yield after flash column chromatography.

The reaction proceeded well for a diverse collection of primary boronic esters including those bearing nitrile (**10**), ester (**11**) and azide (**12**) functional groups. A lower yield was observed for the azide **12** presumably due to competing nucleophilic addition of the organolithium **2‐Li** to the azide in the starting boronic ester. Reaction with a complex lithocholic acid derivative **13** also proceeded in good yield. Secondary boronic esters also worked well, with examples including cyclohexyl **14**, cyclopropyl **17**, *N*‐Boc‐pyrrolidine **18** and piperidines **19** and **21**. Although α‐amino substrates are poor migrating groups,^[20a]^ they nevertheless proceeded in moderate yields (**18** and **21**). Furthermore, using chiral and non‐racemic boronic esters the 1,2‐metalate rearrangement was found to be completely stereospecific (**15** and **16**). To demonstrate scalability, *N*‐Boc‐piperidine **19** was prepared on gram scale. In the case of the menthyl derivative **20**, little product was formed but switching to the less hindered neopentyl glycol ester resulted in an increased 60 % yield. Unusually, the neopentyl glycol boronic ester product was stable to silica gel chromatography. This hindered secondary boronic ester turned out to be the limit of reactivity with secondary TIB esters. No boronate complex was observed by in situ IR spectroscopy with *t*‐Bu pinacol boronic ester but boronate was observed using the neopentyl glycol ester (see Supporting Information for details). However, despite formation of the neopentyl glycol boronate complex, no product was obtained after attempted 1,2‐metalate rearrangement. Presumably, the hindered boronate complex reversed to starting materials upon heating. Similar observations were observed with acyclic secondary TIB esters, indicating that it is apparently too demanding for this methodology to place two quaternary centres next to each other using boronic esters, although this problem could be overcome using boranes.^[21]^

A range of sp^2^ boronic esters were also explored. Both electron poor and electron rich aromatics **22**–**24**, as well as heteroaromatics, such as benzofuran **26** and indole **27** worked well giving the products in good yield. Finally, alkenyl boronic esters performed well, providing tertiary allylic boronic esters **28**–**30** in good yields.

**Scheme 5:**
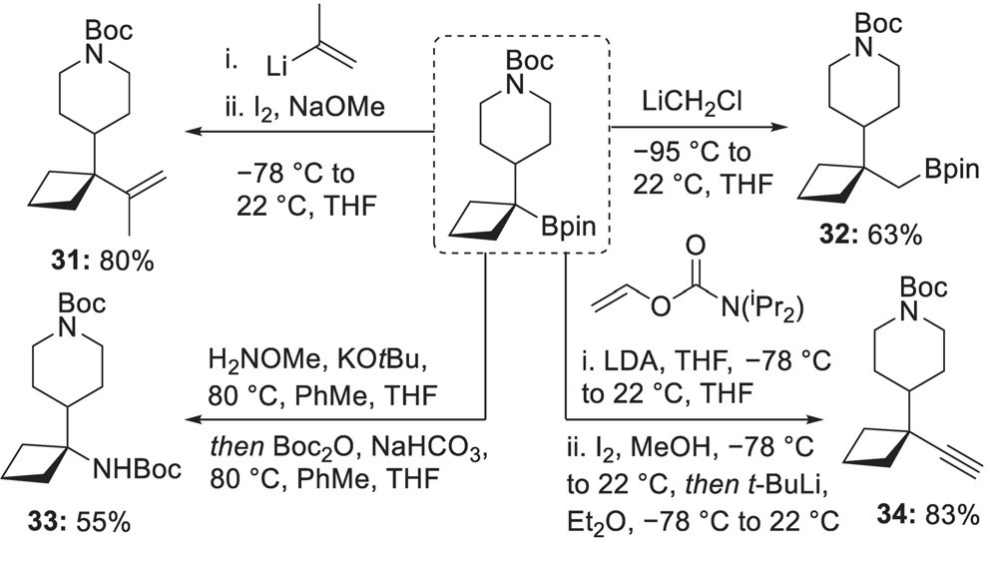
Boronic ester transformations.

To further illustrate the utility of the cyclobutyl boronic ester products, we transformed the boronic ester functionality present in substrate **19** into a range of functional groups (Scheme 5). Zweifel olefination with propenyllithium gave the olefin **31** in excellent yield,^[22]^ and alkynylation with vinyl carbamate gave the alkyne **34** in high yield.^[23]^ The tertiary boronic ester underwent a Matteson homologation to give a primary boronic ester product **32**.^[24]^ The boronic ester was also converted into the tertiary amine in moderate yield, which was protected as the carbamate **33**.^[25]^

## Conclusion

We have studied the lithiation‐borylation of a series of cyclic TIB esters and have shown that the success of the process is governed by a delicate balance of factors involving ease of lithiation, stability of the organolithium, and ease of 1,2‐migration. Of the ring sizes studied, deprotonation became progressively slower going from 3‐ → 6‐membered rings, with the cyclohexyl substrate being too slow to be practical. The organolithium intermediate was prone to α‐elimination and while the lithiated 3‐ and 4‐membered rings were stable, the lithiated cyclopentyl TIB ester underwent α‐elimination faster than deprotonation. The 3‐ and 4‐membered rings both underwent rapid deprotonation and trapping with boronic esters but the 3‐membered ring did not undergo 1,2‐metalate rearrangement, presumably because of the high strain in the TS of the migration. The cyclobutyl ring did undergo 1,2‐metalate rearrangement. Thus, the cyclobutyl ring occupies a “Goldilocks zone”, where the ring is small enough to promote deprotonation, but large and flexible enough to allow the 1,2‐metalate rearrangement to occur, and the organolithium is sufficiently stable. The process shows broad substrate scope, and the applicability of these boron substituted cyclobutanes has been demonstrated by transforming the boronic ester into a range of functional groups.

## Conflict of interest

The authors declare no conflict of interest.

## Supporting information

As a service to our authors and readers, this journal provides supporting information supplied by the authors. Such materials are peer reviewed and may be re‐organized for online delivery, but are not copy‐edited or typeset. Technical support issues arising from supporting information (other than missing files) should be addressed to the authors.

Supplementary
