# Supplementary material for: Studies on the Lithiation, Borylation, and 1,2‐Metalate Rearrangement of O‐Cycloalkyl 2,4,6‐Triisopropylbenzoates
Source: Angew Chem Int Ed Engl. 2021 Apr 12;60(20):11436–41. doi: 10.1002/anie.202101374 (PMC8251992; doi:10.1002/anie.202101374)
Supplement: Supplementary file 1 — Supplementary [file ANIE-60-11436-s001.pdf]

Angewandte Chemie
Eine Zeitschrift der Gesellschaft Deutscher Chemiker

Supporting Information

Studies on the Lithiation, Borylation, and 1,2-Metalate Rearrangement of *O*-Cycloalkyl 2,4,6-Triisopropylbenzoates

*Rory C. Mykura, Pradip Songara, Eugenia Luc, Jack Rogers, Ellie Stammers, and Varinder K. Aggarwal**

anie_202101374_sm_miscellaneous_information.pdf

Return to contents

Contents

1 General Information 4

1.1 React-IR Procedure 5

2 Preparation of benzoate/carbamate starting materials 6

3 Preparation of boronic ester starting materials 12

3.1 Commercially available Boronic Esters: 12

3.2 Boronic esters synthesised using literature procedures 12

4 Optimisation using in situ IR spectroscopy 15

4.1 Cyclobutyl benzoate 15

4.2 Cyclobutyl Carbamate 16

4.3 Attempts to use teritary boronic esters with cyclobutyl benzoate 17

4.4 Cyclopropyl benzoate 18

4.4.1 Failed 1,2-migration attempts. 19

4.5 Cyclopentyl benzoate 20

4.6 Cyclopentyl carbamate 22

4.7 Cyclohexyl TIB ester 23

4.8 In Situ IR spectroscopy 3D traces 25

5 Attempts to use chloro/bromocyclobutane 25

6 Lithiation-Borylation of cyclobutyl benzoate 28

6.1 General procedures 28

6.2 Substrate Scope 29

6.2.1 Primary boronic esters 29

6.2.2 Secondary boronic esters 34

6.2.3 sp^2 boronic esters 42

6.3 Boronic ester functionalisation using boronic ester 19 49

6.3.1 Zweifel Olefination 49

6.3.2 Matteson Homologation 50

6.3.3 Amination 51

6.3.4 Alkynalation 52

7 Spectra 54

8 References 87

Return to contents

Return to contents

1 General Information

Anhydrous solvents were either dried using an Anhydrous Engineering alumina column drying system (THF, toluene, CH_2Cl_2) or obtained as Acroseal bottles and used directly. All other employed solvents were reagent grade solvents and were used directly. Petroleum ether refers to the fraction collected between 40 – 60 °C. Reactions requiring anhydrous conditions (where specified) were conducted under a N_2 / Argon atmosphere using standard Schlenk techniques unless otherwise stated. All reagents were purchased from commercial sources and used as received, unless otherwise stated. Flash column chromatography was carried out using Aldrich silica gel (40-63 μm) or on a Biotage isolera one machine (following λ_{all}) using the column and gradient specified (CVs = column volumes). Reactions were monitored by thin-layer chromatography (TLC) when practical, using Merck Kieselgel 60 F254 fluorescent treated silica which was visualized under UV light (254 nm) or by staining with an aqueous basic potassium permanganate or *p*-anisaldehyde solution as stated. **^{1}H NMR** spectra were recorded using either Jeol ECS/ECZ 400 MHz, Bruker 400 MHz, Bruker Cryo 500 MHz, or Varian VNMR (400 MHz or 500 MHz) spectrometers. Chemical shifts (*δ*) are given in parts per million (ppm) and coupling constants (*J*) are given in Hertz (Hz). **^{13}C NMR** spectra were recorded using either Jeol ECS/ECZ 400 MHz, Bruker (101) MHz, Bruker Cryo 126 MHz, or Varian VNMR (101 MHz or 126 MHz). Use of "**/**" (forward slash) in assignments (for example "**6/7**") refers to environments **6 or 7**. **High resolution mass spectra** (**HRMS**) were recorded on a Bruker Daltonics Apex IV typically by Electrospray Ionisation (ESI) or the method stated. **IR** spectra were recorded on a Perkin Elmer Spectrum One FT-IR as a thin film. Only selected absorption maxima (ν_{max}) are reported in wavenumbers (cm^{-1}). Melting points were recorded in degrees Celsius (°C) using a Stuart SMP30 melting point apparatus. **Optical rotations** (**$[\alpha]^D$ T**) were measured on a Bellingham & Stanley Ltd. ADP 220 polarimeter. ADP220 polarimeter and is quoted in (° ml)(g dm)$^{-1}$. **Chiral HPLC** was performed on a HP Agilent 1100 with the isocratic gradient specified and the column specified, monitoring by DAD (Diode Array Detector), usually at 210 nm unless otherwise specified.

Return to contents

1.1 React-IR Procedure

React-IR setup standard procedure:

To set up a reaction with the React-IR probe, a 3 necked 25 mL flask was used. The recently cleaned probe was then inserted into the top of the flask using a Teflon adaptor (supplied with the instrument) inside a glass adaptor, secured with a clip, and placed at a height where it would be immersed in the reaction mixture, but did not touch the stirring bar. The contrast and align, performance (5 runs) and stability (duration of 5 minutes) tests were then performed and saved. After the tests, the flask was placed under vacuum and dried with a heat gun. A new experiment could then be started. A background could be taken in the solvent to remove any solvent peaks, however this could also be achieved using the software's solvent subtraction, once a solvent reference spectrum had been taken. The carbonyl peaks observed did not overlap with solvent. Once set up, before clicking the 'start experiment button' all starting materials were added and the reaction cooled to the desired temperature for 30 minutes. The expression 'reaction temperature' refers to temperatures of cooling bath mixtures which result in temperatures of either −60 °C or −78 °C. The resistance temperature detector (RTD) on the probe was not used.

Note: It is important to turn the React-IR instrument on 1 – 3 hours before an experiment is started, usually the night before. Once ready the 'TEMP OK' display under the 'contrast and align' test will change from 'false' to 'okay'. This could take anywhere from 1 – 3 hours.

Return to contents

2 Preparation of benzoate/carbamate starting materials

1 Cyclopropyl 2,4,6-triisopropylbenzoate

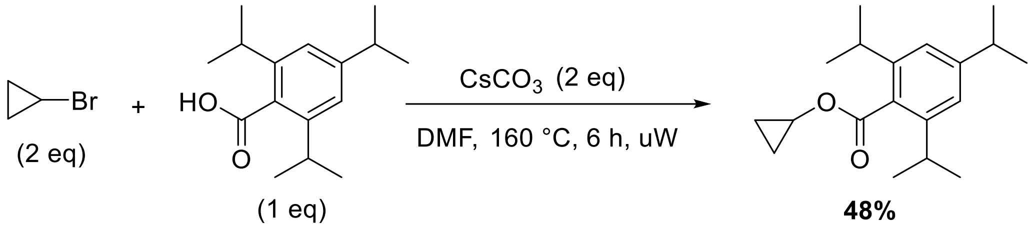

In a 5.0 mL microwave vial were combined 2,4,6-triisopropylbenzoic acid (0.497 g, 2 mmol, 1.0 eq) and DMF (1.0 mL, not anhydrous). $CsCO_3$ (1.30 g, 4 mmol, 2.0 eq) was added and the mixture stirred for 10 minutes. Bromocyclopropane (0.32 mL, 0.434 g, 4 mmol, 2.0 eq) was added in a single portion and the reaction heated to 160 °C for 6 h with rapid stirring. The brown mixture was then filtered through a plug of SiO_2 (washing with Et_2O) and the DMF removed under reduced pressure (toluene (3 mL x 3) was added and removed to aid DMF removal) to give a crude oil. This was then purified twice on a Biotage system using a 50 g Ultra column, 1 to 8% Et_2O in pentane [1-11-2] CVs, to give the desired product (0.279 g, 48%) as a clear oil.

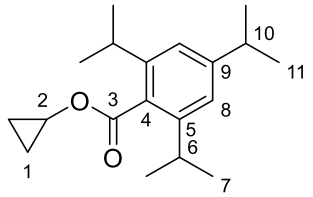

R*f* = 0.32 (4% Et_2O in pentane, UV/anisaldehyde).

^{1}H NMR (400 MHz, $CDCl_3$): *δ* = 6.99 (s, 2H, **8**), 4.43 – 4.37 (m, 1H, **2**), 2.94 – 2.80 (m, 3H, **6 & 10**), 1.24 & 1.23 (2 x d, *J* = 6.9 Hz, 18H, **7 & 11**), 0.83 – 0.77 (m, 4H, **1**).

^{13}C NMR (101 MHz, $CDCl_3$): *δ* = 171.8 (**3**), 150.4 (**5/9**), 144.9 (**5/9**), 130.3 (**4**), 121.0 (**8**), 49.4 (**2**), 34.6 (**6/10**), 31.5 (**6/10**), 24.2 (**7/11**), 24.1 (**7/11**), 5.2 (**1**).

All data agree with a previous report.[1]

Return to contents

2 Cyclobutyl 2,4,6-triisopropylbenzoate

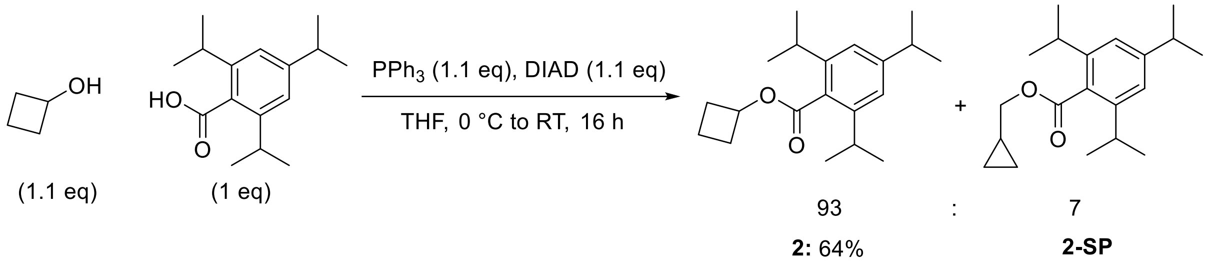

To a flame-dried flask under nitrogen was added 2,4,6-triisopropylbenzoic acid (TIBOH, 6.26 g, 25.2 mmol, 1.00 eq) and PPh_3 (7.26 g, 27.7 mmol, 1.10 eq). The flask was then treated to three vacuum nitrogen cycles with stirring. THF (25.2 mL, 1 M) and cyclobutanol (2.17 mL, 27.7 mmol, 1.10 equiv.) were added. The flask was cooled to 0 °C and DIAD (5.44 mL, 27.7 mmol, 1.10 eq) was added dropwise over a period of 15 minutes. After 10 minutes, the reaction was warmed to room temperature and left to stir at room temperature for 16 h. The crude material was concentrated *in vacuo* and the residue was dissolved in minimal pentane (<25 mL) and stirred vigorously at room temperature for 2 minutes. The resultant white precipitate was filtered and washed with pentane (<100 mL). The filtrate was concentrated *in vacuo* and purified by column chromatography (3% to 6% Et_2O/petrol) to afford the mixture of products **2** **& 2-SP** (5.26 g, 93:7 ratio of desired benzoate **2** to inseparable cyclopropylmethyl benzoate **2-SP,** 64% desired product).

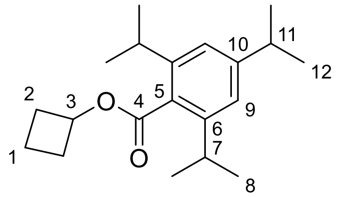

R_f = 0.57 (10% Et_2O/petrol, *p*-anisaldehyde);

^{1}H NMR (400 MHz, $CDCl_3$): δ = 7.00 (s, 2H, **9**), 5.25 (1H, p, J = 7.5 Hz, **3**), 2.94 – 2.82 (m, 3H, **7/11**), 2.51 – 2.40 (m, 2H, **2**), 2.23 – 2.11 (m, 2H, **2**), 1.91 – 1.80 (m, 1H, **1**), 1.77 – 1.64 (m, 1H, **1**), 1.25 (d, J = 6.8 Hz, 12H, **8**), 1.24 (d, J = 6.8 Hz, 6H, **12**).

^{13}C NMR (101 MHz, $CDCl_3$): δ = 170.3 (**4**), 150.2 (**6/10**), 144.9 (**6/10**), 130.7 (**5**), 121.0 (**9**), 69.4 (**3**), 34.6 (**7/11**), 31.5 (**7/11**), 30.5 (**2**), 24.3 (**9/13**), 24.1 (**9/13**), 14.1 (**1**).

HRMS-ESI$^+$ (*m/z*): [M + Na]+ calculated for $C_{20}H_{30}O_2Na$, 325.2143; found, 325.2140;

IR (ν_{max}/cm^{-1}, neat): 2959, 2869, 1721 (C=O), 1606, 1463, 1248, 1136, 1080, 937, 879, 755, 612.

3 Cyclopentyl 2,4,6-triisopropylbenzoate

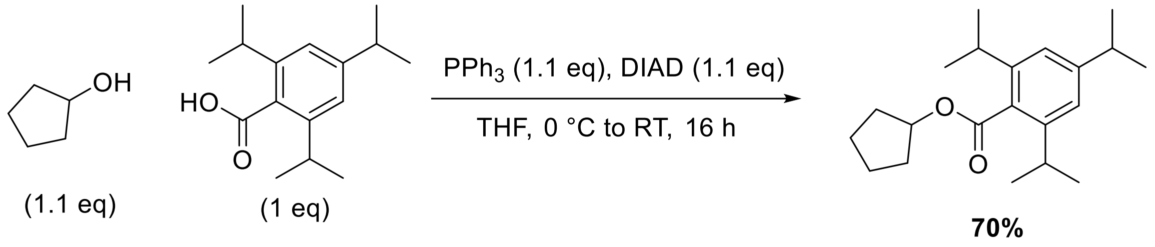

Following the same procedure as for the cyclobutyl benzoate (2) using TIBOH (1.24 g, 5 mmol, 1.0 eq), cyclopentanol (0.5 mL, 0.474 g, 5.5 mmol, 1.1 eq), PPh_3 (1.44 g, 5.5 mmol, 1.1 eq), DIAD (1.1 mL, 1.11 g, 5.5 mmol, 1.1 eq) and THF (25 mL) to give the desired product (1.10 g, 70%) as a white solid.

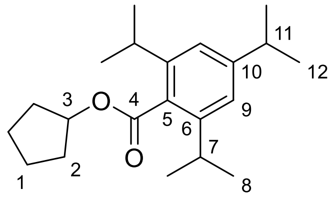

R_f = 0.31 (4% Et_2O in pentane, $KMnO_4$)
^{1}H NMR (400 MHz, $CDCl_3$): δ = 7.01 (s, 2H, **9**), 5.47 (tt, J = 5.9, 2.9 Hz, 1H, **3**), 2.90 (hept, J = 6.8 Hz, 3H, **7 & 11**), 2.02 – 1.90 (m, 2H, **2**), 1.90 – 1.81 (m, 2H, **2**), 1.80 – 1.70 (m, 2H, **1**), 1.70 – 1.56 (m, 2H, **1**), 1.26 (d, J = 6.9 Hz, 12H, **8**), 1.25 (d, J = 7.0 Hz, 6H, **12**).

^{13}C NMR (101 MHz, $CDCl_3$): δ = 170.7 (**4**), 150.1 (**6/10**), 144.8 (**6/10**), 131.0 (**5**), 120.9 (**9**), 77.9 (**3**), 34.6 (**7/11**), 32.8 (**2**), 31.4 (**7/11**), 24.3 (**8/12**), 24.10 (**8/12**), 23.8 (**1**).

HRMS-ESI$^+$ (m/z): $[M + H]^+$ calculated for $C_{21}H_{33}O_2$, 317.2475; found, 317.2466.

IR (ν_{max}/cm^{-1}, neat): 2959.2, 2869.9, 1708.6, 1606.7, 1461.0, 1253.8, 1078.3.
Melting point: 51 – 52 °C (pentane).

4 Cyclohexyl 2,4,6-triisopropylbenzoate

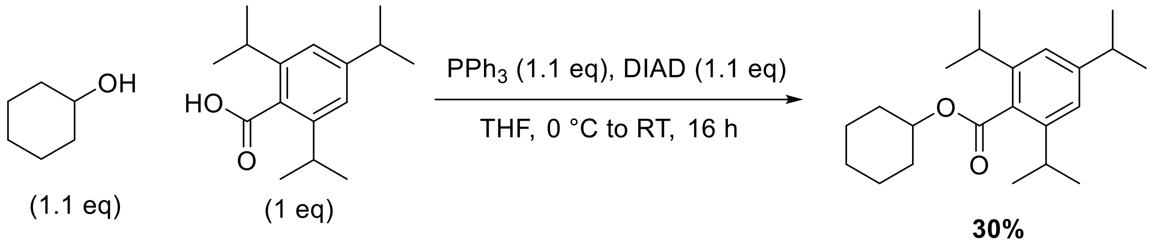

30%

Following the same procedure as for the cyclobutyl benzoate (2) using TIBOH (1.24 g, 5 mmol, 1.0 eq), cyclohexanol (0.58 mL, 0.474 g, 5.5 mmol, 1.1 eq), PPh_3 (1.44 g, 5.5 mmol, 1.1 eq), DIAD (1.1 mL, 1.11 g, 5.5 mmol, 1.1 eq) and THF (25 mL) gave the desired product (0.494 g, 30%) as a white solid.

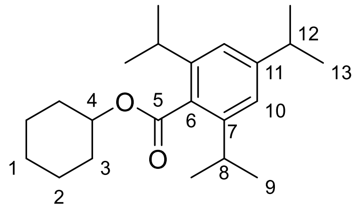

R_f = 0.34 (4% Et_2O in pentane, $KMnO_4$)

^{1}H NMR (400 MHz, $CDCl_3$): δ = 7.01 (s, 2H, **10**), 5.13 – 5.02 (m, 1H, **4**), 2.91 (hept, J = 6.8 Hz, 2H, **8**), 2.89 (hept, J = 6.9 Hz, 1H, **12**), 2.06 – 1.96 (m, 2H, **3**), 1.84 – 1.72 (m, 2H, **2**), 1.64 – 1.55 (m, 1H, **1**), 1.55 – 1.46 (m, 2H, **3**), 1.46 – 1.37 (m, 2H, **2**), 1.32 – 1.29 (m, 1H, **1**), 1.26 (d, J = 6.8 Hz, 12H, **9**), 1.25 (d, J = 6.9 Hz, 6H, **13**).

^{13}C NMR (101 MHz, $CDCl_3$): δ = 170.3 (**5**), 149.9 (**7/11**), 144.6 (**7/11**), 131.0 (**6**), 120.8 (**10**), 73.5 (**4**), 34.5 (**12**), 31.8 (**3**), 31.3 (**8**), 25.4 (**1**), 24.1 (**9/13**), 24.0 (**9/13/2**), 23.9 (**9/13/2**).

HRMS-ESI$^+$ (m/z): $[M + H]^+$ calculated for $C_{22}H_{35}O_2$, 331.2632; found, 331.2632.

IR (ν_{max}/cm^{-1}, neat): 2957.5, 2932.6, 2863.8, 1714.9, 1608.5, 1250.9, 1077.2.

Melting point: 50 – 51 °C (pentane).

Return to contents

2-Cb Cyclobutyl carbamate

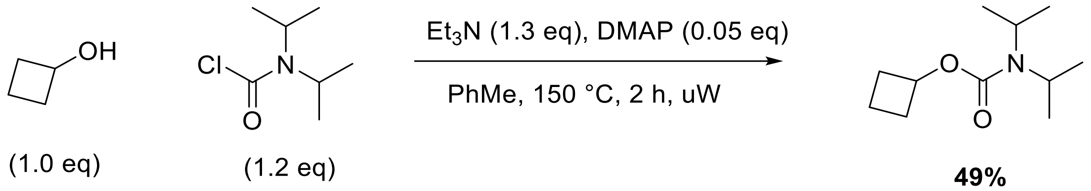

N,N-diisopropylcarbamoyl chloride (1.31 g, 8 mmol, 1.2 eq), DMAP (40.7 mg, 0.3 mmol, 5 mol%), NEt_3 (1.2 mL, 8.7 mmol, 1.3 equiv) and cyclobutanol (0.52 mL, 6.7 mmol, 1 eq) were added to a microwave vial and dissolved in toluene (5 mL, 1.33 mmol mL^{-1}). The mixture was stirred to complete dissolution and heated in a microwave reactor for 2 hours at 150 °C. The white precipitate was filtered off using a silica plug and the resulting crude mixture was concentrated in vacuo. Purification by column chromatography (5% to 20% Et2O in petrol) gave the desired product (781 mg, 49%) as a pale-yellow oil.

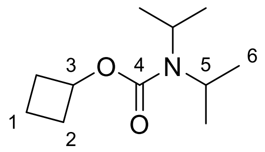

***R**$_f$* = 0.44 (1:1 petrol:Et_2O, anisaldehyde/PMA)

^{1}H NMR (400 MHz, $CDCl_3$): δ = 4.97 (p, J = 7.5 Hz, 1H, **3**), 3.89 (brs, 2H, **5**), 2.38 – 2.29 (m, 2H, **2**), 2.13 – 2.00 (m, 2H, **2**), 1.82 – 1.71 (m, 1H, **1**), 1.66 – 1.52 (m, 1H, **1**), 1.20 (d, J = 6.8 Hz, 12H, **6**).

^{13}C NMR (101 MHz, $CDCl_3$): δ = 155.14 (**4**), 68.48 (**3**), 30.67(**2**), 21.02 (**6**), 13.44 (**1**).

HRMS-ESI+ (m/z): $[M+Na]^+$ calculated for $C_{11}H_{21}NO_2Na$, 222.1471; found, 222.1467.

IR (ν_{max} / cm^-1, neat): 2970, 1686, 1434, 1307, 1290, 1134, 1088.

3-Cb Cyclopentyl diisopropylcarbamate

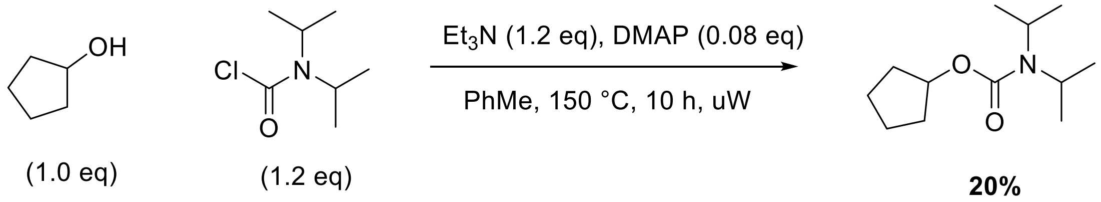

Following a similar procedure as for cyclobutyl carbamate (2-Cb) using carbomyl chloride (1.57 g, 9.6 mmol, 1.2 eq), cyclopentanol (0.73 mL, 0.689 g, 8.0 mmol, 1.0

eq), Et_3N (1.34 mL, 0.971 g, 9.6 mmol, 1.2 eq), DMAP (0.078 g, 0.64 mmol, 0.08 eq) and toluene (8 mL), with heating for 10 h, gave the desired product (0.656 g, 20%) as a colourless oil.

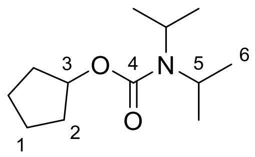

R_f = 0.4 (10% Et_2O in pentane, PMA)
^{1}H NMR (400 MHz, $CDCl_3$): δ = 5.14 (tt, J = 5.6, 2.5 Hz, 1H, **3**), 4.59 – 3.17 (brs, 2H, **5**), 1.87 – 1.77 (m, 2H, **2**), 1.77 – 1.67 (m, 4H, **2 & 1**), 1.64 – 1.54 (m, 2H, **1**), 1.17 (d, J = 6.9 Hz, 12H, **6**).

^{13}C NMR (101 MHz, $CDCl_3$): δ = 155.9 (**4**), 77.2 (**3**), 45.5 (**5**, br), 33.0 (**2**), 23.9 (**1**), 21.2 (**6**, br).

HRMS-ESI$^+$ (m/z): $[M + H]^+$ calculated for $C_{12}H_{24}NO_2$, 214.1802; found, 214.1807.

IR (ν_{max}/cm^{-1}, neat): 2966.1, 2873.4, 1683.0, 1287.4, 1049.1, 772.2.

[Return to contents](#)

3 Preparation of boronic ester starting materials

3.1 Commercially available Boronic Esters:

The following boronic esters were purchased from suppliers:

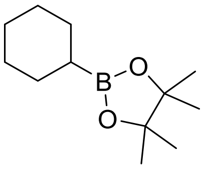

Alfa Aesar

CAS = 87100-15-0

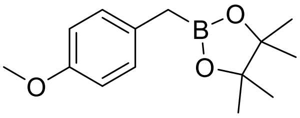

TCI

CAS = 475250-52-3

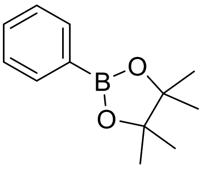

Merck

CAS = 24388-23-6

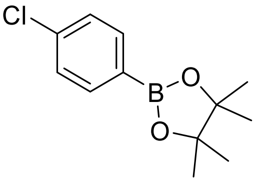

Merck

CAS = 195062-61-4

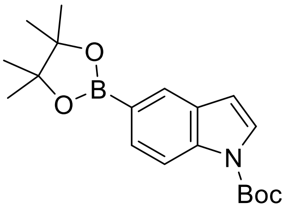

Fluorochem

CAS = 777061-36-6

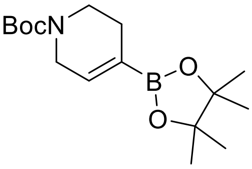

Fluorochem

CAS = 286961-14-6

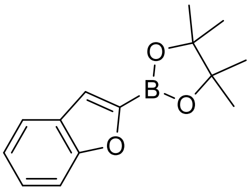

Fluorochem

CAS = 402503-13-3

3.2 Boronic esters synthesised using literature procedures

4,4,5,5-Tetramethyl-2-phenethyl-1,3,2-dioxaborolane

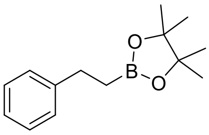

Synthesised following a literature procedure. *Org. Synth.*, **2017**, 94, 234-251.

2-(3-Azidopropyl)-4,4,5,5-tetramethyl-1,3,2-dioxaborolane

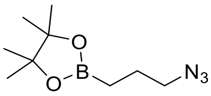

Synthesised following a literature procedure. *J. Am. Chem. Soc.* **2017**, 9519–9522

tert-Butyl(((3R,8R,9S,10S,13R,14S,17R)-10,13-dimethyl-17-((R)-4-(4,4,5,5-tetramethyl-1,3,2-dioxaborolan-2-yl)butan-2-yl)hexadecahydro-1H-cyclopenta[a]phenanthren-3-yl)oxy)dimethylsilane

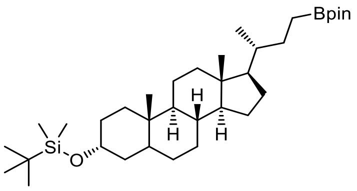

Synthesised following a literature procedure. *Science*, **2017**, *357*, 283–286.

***tert*-Butyl 3-(4,4,5,5-tetramethyl-1,3,2-dioxaborolan-2-yl)propanoate**

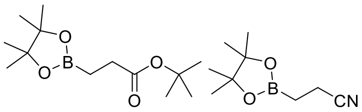

Synthesised following a literature procedure. *Chem. Commun.*, **2011**, *47*, 12592-12594

4,4,5,5-tetramethyl-2-(*trans*-2-phenylcyclopropyl)-1,3,2-dioxaborolane

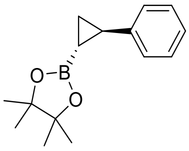

Synthesised following a literature procedure. *Science*, **2017**, *357*, 283–286.

2–((1R,2R,5R)–2–isopropyl–5–methylcyclohexyl)–5,5–dimethyl–1,3,2–dioxaborinane

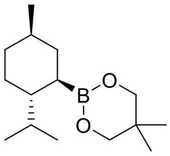

Synthesised following a literature procedure. *Angew. Chem. Int. Ed.*, **2017**, *56*, 9752–9756

Note: Synthesis requires 2 x precipitated CuCl and recrystalised B_2neo_2.

***tert*-Butyl 2-(4,4,5,5-tetramethyl-1,3,2-dioxaborolan-2-yl)pyrrolidine-1-carboxylate**

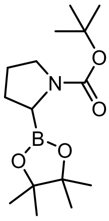

Synthesised following a literature procedure. *Angew. Chem. Int. Ed.*, **2017**, *56*, 2127–2131

(S)-4,4,5,5-tetramethyl-2-(1-phenylethyl)-1,3,2-dioxaborolane

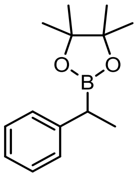

Synthesised following a literature procedure. e.e. determined as 96:4 after oxidation to the corresponding alcohol. *Angew. Chem. Int. Ed.* **2014**, *53*, 9846-9850

tert-butyl 4-(4,4,5,5-tetramethyl-1,3,2-dioxaborolan-2-yl)piperidine-1-carboxylate

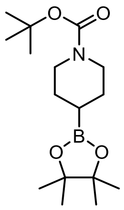

Synthesised following a literature procedure. *Science*, **2017**, *357*, 283–286.

(*R*)-2-(4-(4-methoxyphenyl)butan-2-yl)-4,4,5,5-tetramethyl-1,3,2-dioxaborolane

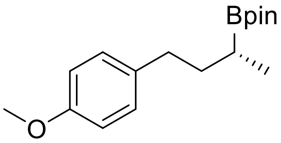

Synthesised following a literature procedure. e.e. determined as 96:4 after oxidation to the corresponding alcohol. *J. Am. Chem. Soc.* **2016**, 138, 9521–9532

***tert*-Butyl 2-(4,4,5,5-tetramethyl-1,3,2-dioxaborolan-2-yl)piperidine-1-carboxylate**

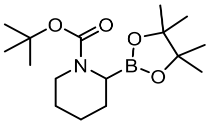

Synthesised following a literature procedure. *Angew. Chem. Int. Ed.*, **2017**, *56*, 2127–2131

4-Methoxyphenyl-4,4,5,5-tetramethyl-[1,3,2]-dioxaborolane

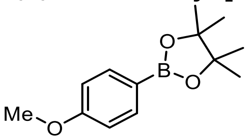

Synthesised following a literature procedure. *Angew. Chem. Int. Ed.*, **2020**, *59*, 1187-1191

(*E*)-4,4,5,5-tetramethyl-2-styryl-1,3,2-dioxaborolane

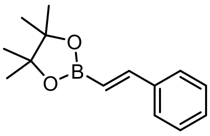

Synthesised following a literature procedure. *Angew. Chem. Int. Ed.*, **2020**, *59*, 1187-1191

(*Z*)-4-(4’,4’,5’,5’-tetramethyl-1’,3’,2’-dioxaboralanyl)-1-(di-*tert*butylmethylsilyloxy)-pent-3-ene

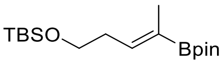

Synthesised following a literature procedure. *Angew. Chem. Int. Ed.*, **2012**, *51*, *12444–12448*.

4 Optimisation using in situ IR spectroscopy

4.1 Cyclobutyl benzoate

The lithiation borylation of cyclobutyl 2,4,6-triisopropylbenzoate **2** was investigated using in situ IR spectroscopy.

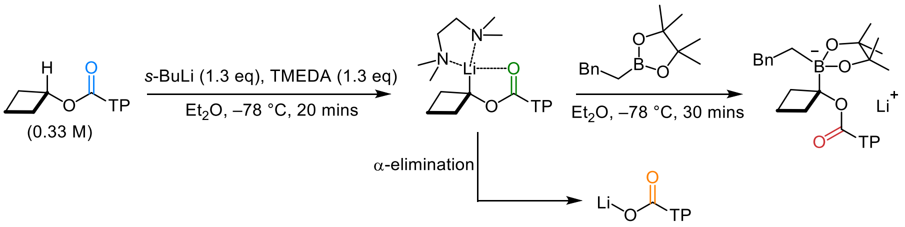

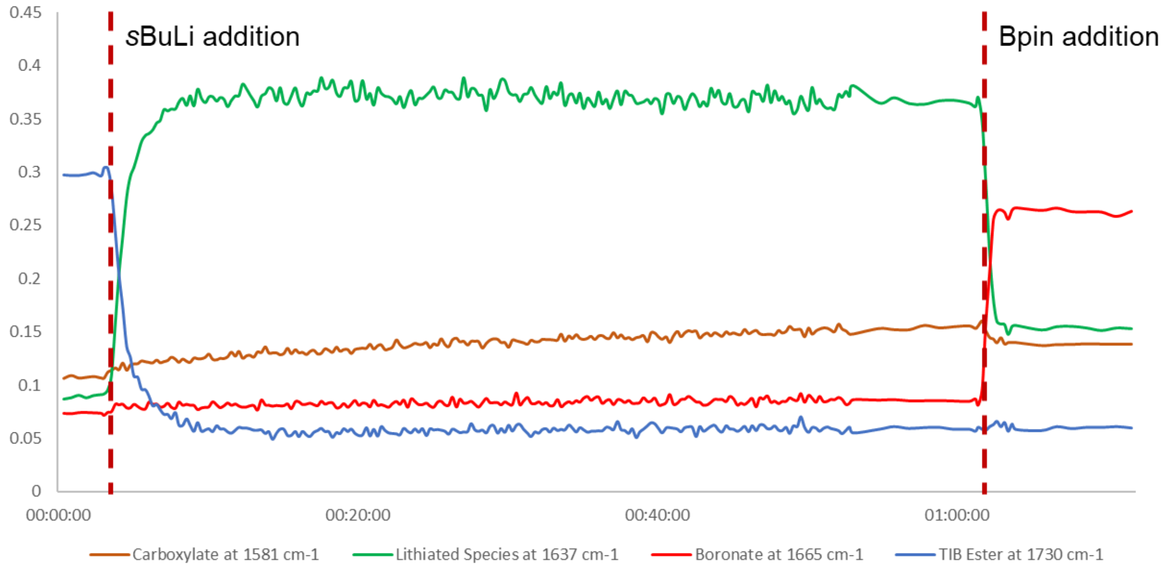

The above in situ IR trace at t = 0 shows an IR stretch at 1730 cm^{-1}, which is in-line with previous reports of similar benzoates. The line (blue) is flat which demonstrates the concentration of benzoate is not changing, as expected. Upon addition of *sec*-BuLi (1.2 eq based on substrate) the signal at 1727 cm^{-1} (blue line) decreases in intensity, with another signal appearing at 1637 cm^{-1} (green line) which is consistent with lithiated benzoates. The lithiation of this TIB ester proceeds without observable intermediates and is complete within *ca*. 10 minutes. Completion is defined as the point in time where the increasing band plateaus. Another broad signal is observed to increase in intensity at 1581 cm^{-1} (orange line) which is believed to be the lithium salt of the benzoate group, deriving from α-elimination of the lithiated species. The signal for the lithiated species at 1637 cm^{-1} continues to drop in intensity, demonstrating a reduction in concentration over time, which is consistent with its apparent instability. Phenethyl boronic acid pinacol ester (1.2 eq based on substrate, 1 M in Et_2O) is then added and the signal for the lithiated species immediately drops, with a concurrent

increase in a new signal of comparatively intermediate wavenumber 1665 cm^{-1} (red line), consistent with previous observations of boronate complexes in solution. Although the lithiated species is tertiary, the lack of a large diamine ligand on lithium still allows for rapid borylation.

Note: Due to the observation of TIB ester remaining at the end of the reaction, the use 1.5 eq of *s*-BuLi and RBpin was taken forward to optimum conditions. The lithiated species can be left for up to 30 mins at −78 °C without a noticeable drop in yield.

4.2 Cyclobutyl Carbamate

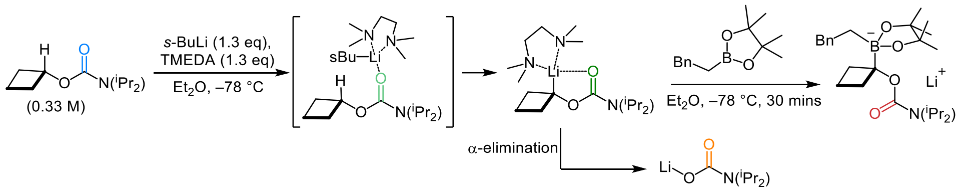

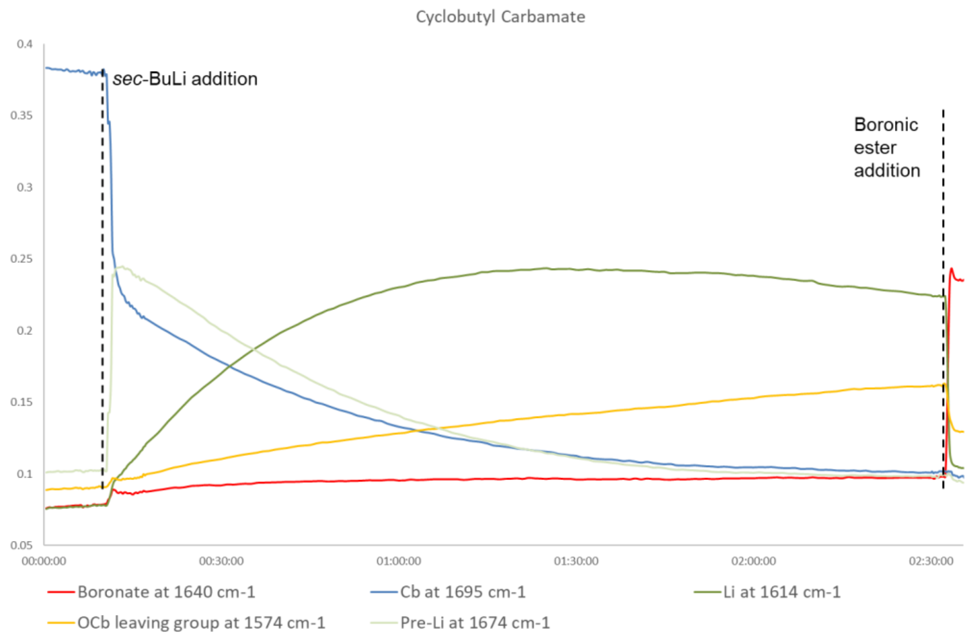

The cyclobutyl carbamate proceeds as for the benzoate, however with the signals shifted to lower wavenumber, in-line with the donation of the nitrogen lone pair into the carbonyl. The carbamate substrate also shows another, transient, peak at 1674 cm^{-1} which is expected to be a “pre-lithiation complex”, in-line with previous reports.[2,3] Some of these complexes are unable to undergo lithiation, and so lead to the observed increased lithiation time (vs. the cyclobutyl benzoate). The borylation of the lithiated species with phenethyl boronic acid pinacol ester (added as a 1 M solution in Et_2O) again occurs essentially instantaneously.

4.3 Attempts to use teritary boronic esters with cyclobutyl benzoate

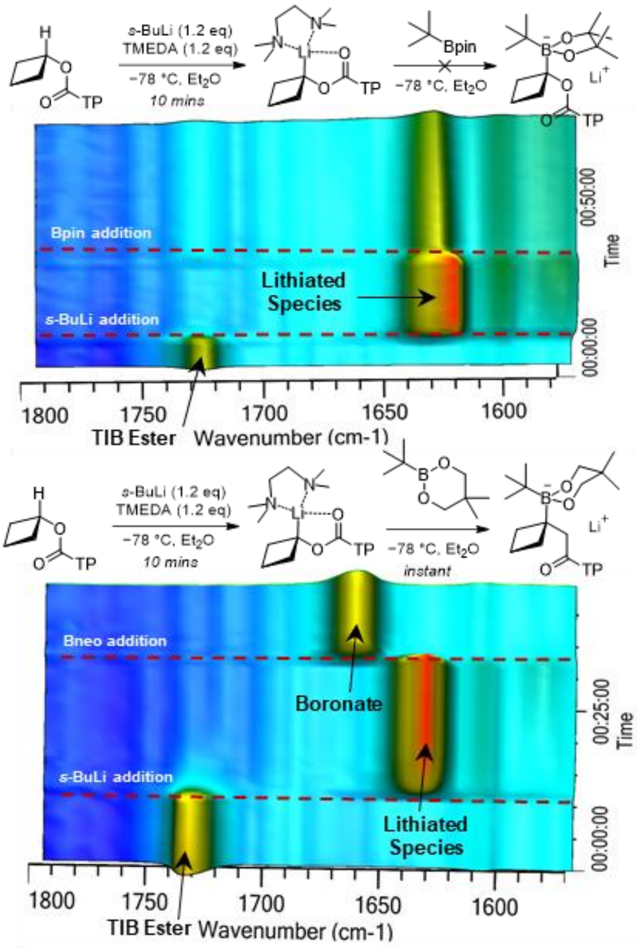

Top scheme: The TIB ester is observed at the usual wavenumber of 1730 cm^{-1}. Addition of s-BuLi leads to the lithiated species at 1640 cm^{-1}. However, addition of *tert*-butyl boronic acid pinacol ester leads to no observable boronate complex. The drop in intensity for the lithiated species is due to dilution. No product was observed after completion of the reaction.

Bottom scheme: The lithiation proceeds as above. Addition of *tert*-butyl boronic acid neopentyl glycol ester leads to instantaneous formation of the boronate complex at 1666 cm^{-1}. Unfortunately, upon warming and attempting 1,2-migratrion under our optimised conditions (solvent switch the $CHCl_3$, 60 °C) no product was observed. Presumably the hindered boronate complex reverses to starting materials which then decompose, rather than undergoing the desired transformation.

Return to contents

4.4 Cyclopropyl benzoate

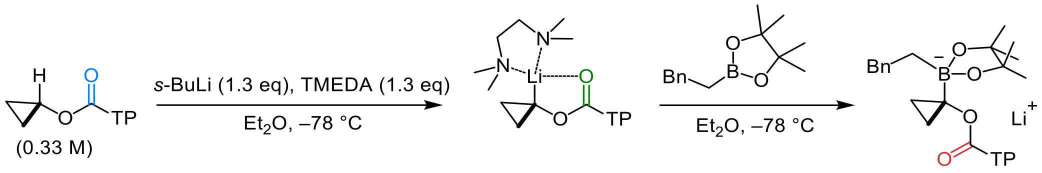

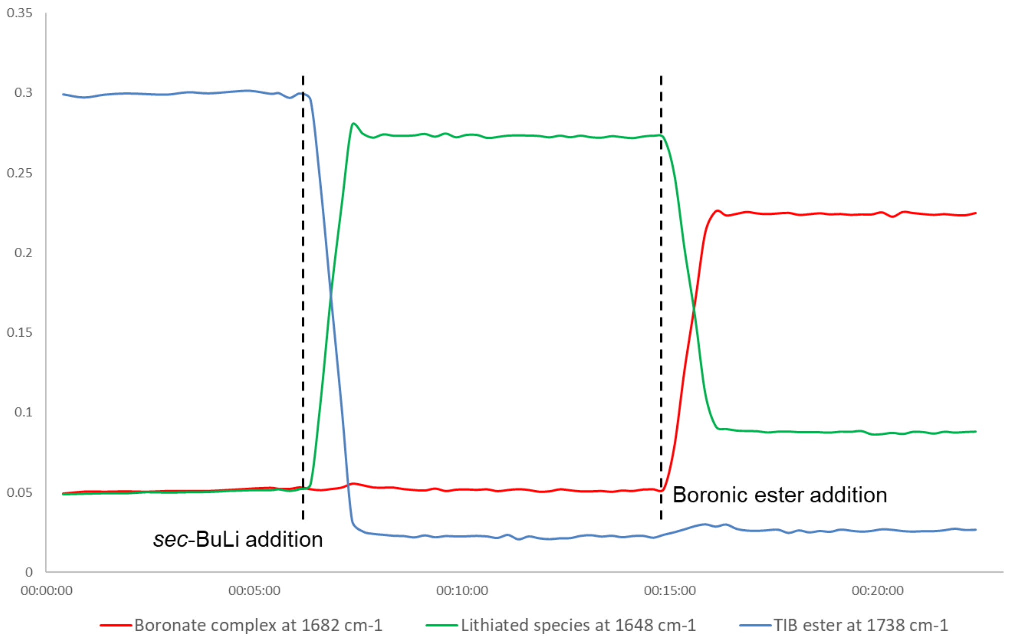

The cyclopropyl benzoate can be observed at 1738 cm^{-1} in Et_2O. Upon addition of *sec*-BuLi (1.3 eq based on substrate) this signal drops in intensity with concurrent increase in another signal at lower wavenumber of 1648 cm^{-1}, expected to be the lithiated benzoate. With the in situ IR machine scanning every 15 s the lithiation is as quick as the addition of *sec*-BuLi, in-line with the increased acidity generated by the cyclopropyl ring. The lithiated species generated is chemically stable at −78 °C, as indicated by the intensity of the line not changing with time, and the lack of a broad signal at ~1581 cm^{-1} for the carboxylate group. Phenethyl boronic acid pinacol ester is then added (1 M solution in Et_2O) leading to a decrease in the peak for the lithiated species and a new peak at 1682 cm^{-1}, consistent with the formed boronate complex. The lithium–boron exchange occurs as quickly as the boronic ester is added, with the in situ IR machine scanning every 15 s.

4.4.1 Failed 1,2-migration attempts.

Unfortunately, despite several attempts, the boronate complex would not undergo 1,2-migration (see table **S1**) presumably due to the strain generated in the transition state by the cyclopropyl ring.

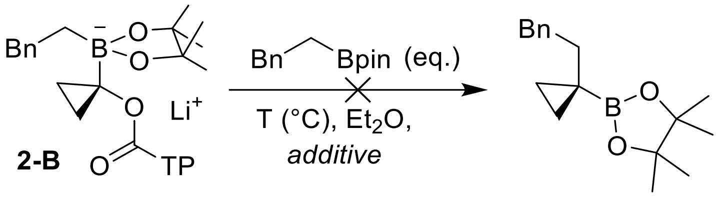

Entry	Additive	Temperature (°C)	Time (h)	Yield
1	None	40	16	0%
2	$CHCl_3$ (solvent swap)	60	16	0%
3	$MgBr_2$•Et_2O	40	16	<5%
4	$MgBr_2$/MeOH	50	16	0%
5	$Mg(ClO_4)_2$/CF_3CH_2OH	50	16	0%

Table **S1**: Attempted 1,2-migration of the cyclopropyl boronate complex **1-B** was unsuccessful.

Return to contents

4.5 Cyclopentyl benzoate

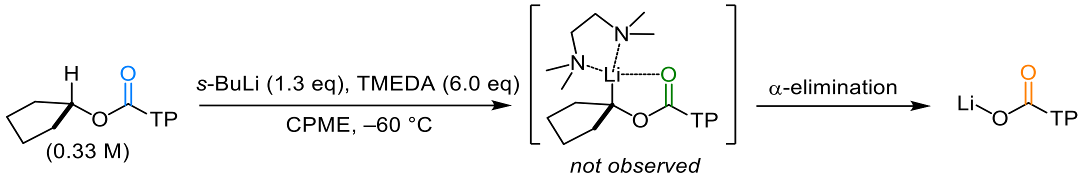

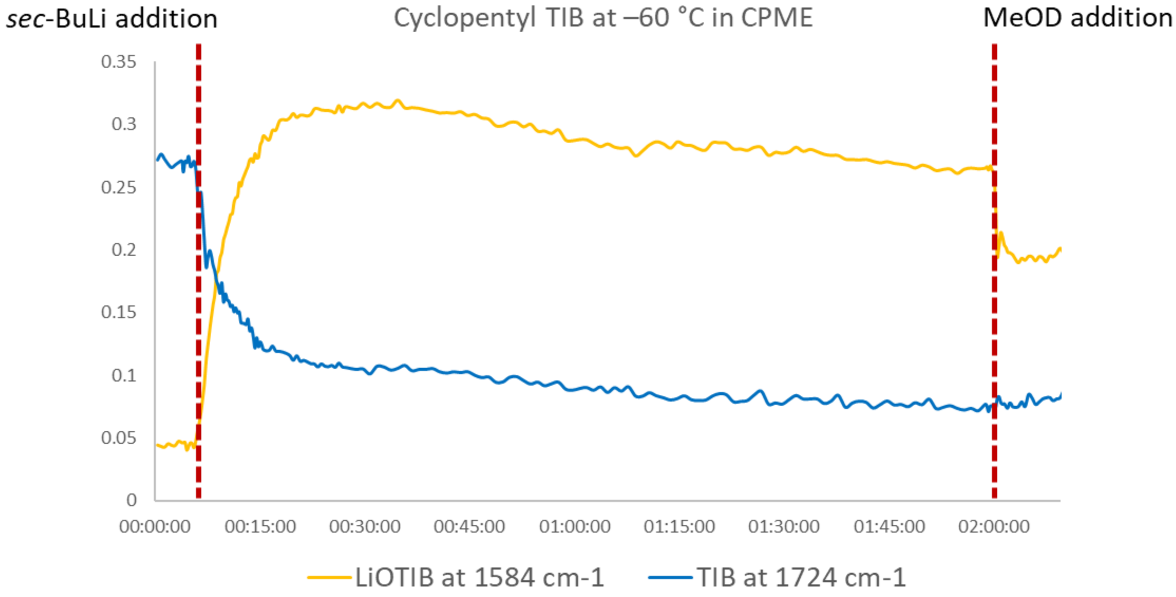

The lithiation half-life, defined as the time taken for half the final concentration of lithiated species to be formed, is $t_{½}$ = 2 minutes. The term half-life does not refer to the (pseudo)molecularity of the process.

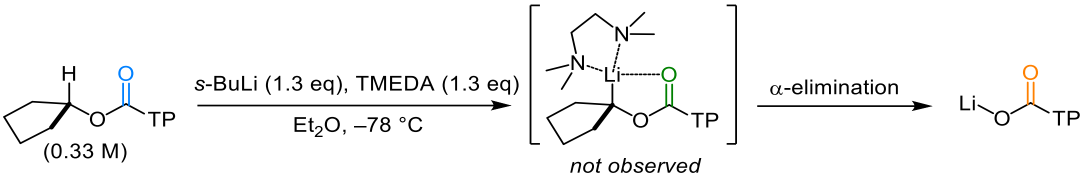

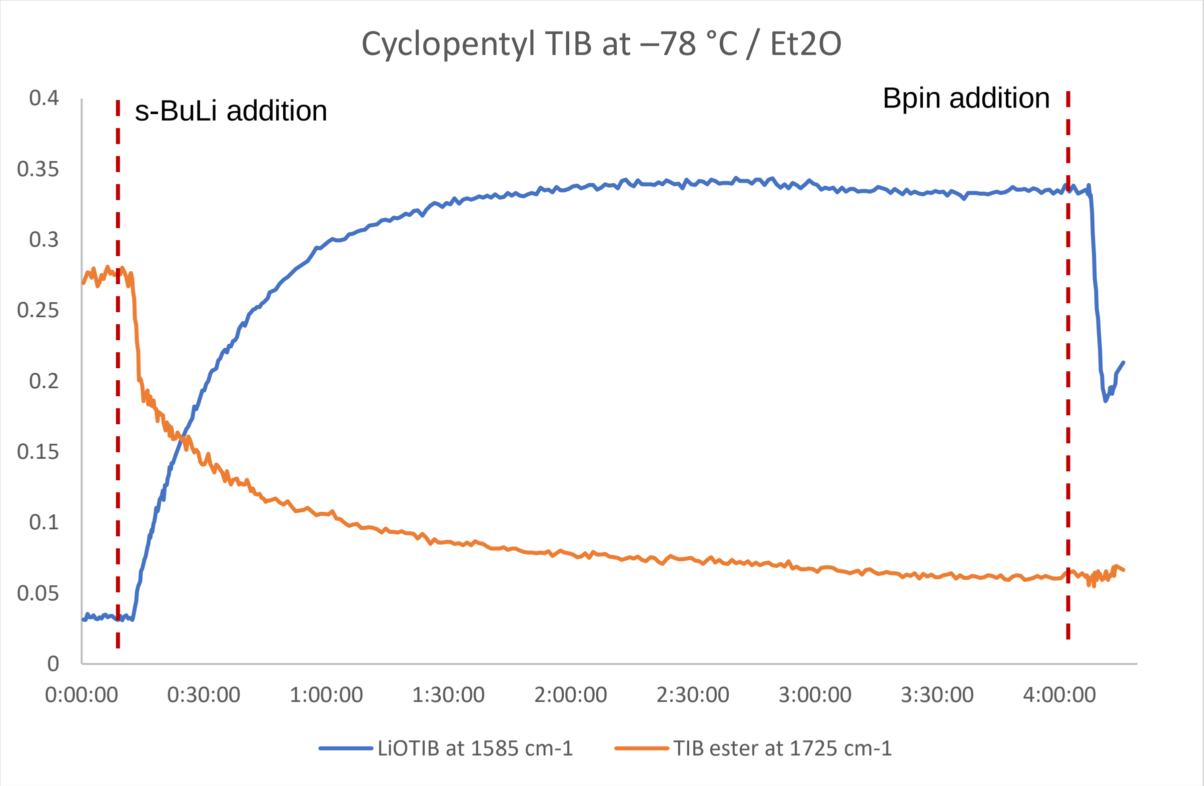

The lithiation half-life, defined as the time taken for half the final concentration of lithiated species to be formed, is $t_{½}$ = 17 minutes. The term half-life does not refer to the (pseudo)molecularity of the process. For comparison, the half-life of lithiation of iso-butyl benzoate under the same conditions is also $t_{½}$ = 17 minutes.

4.6 Cyclopentyl carbamate

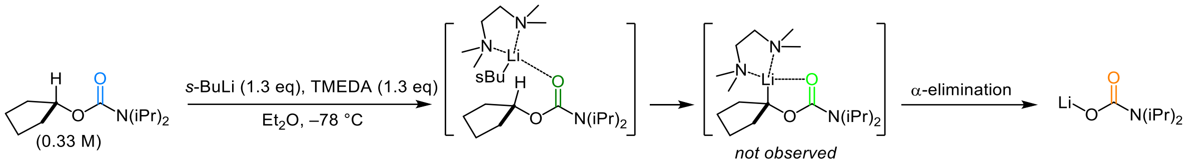

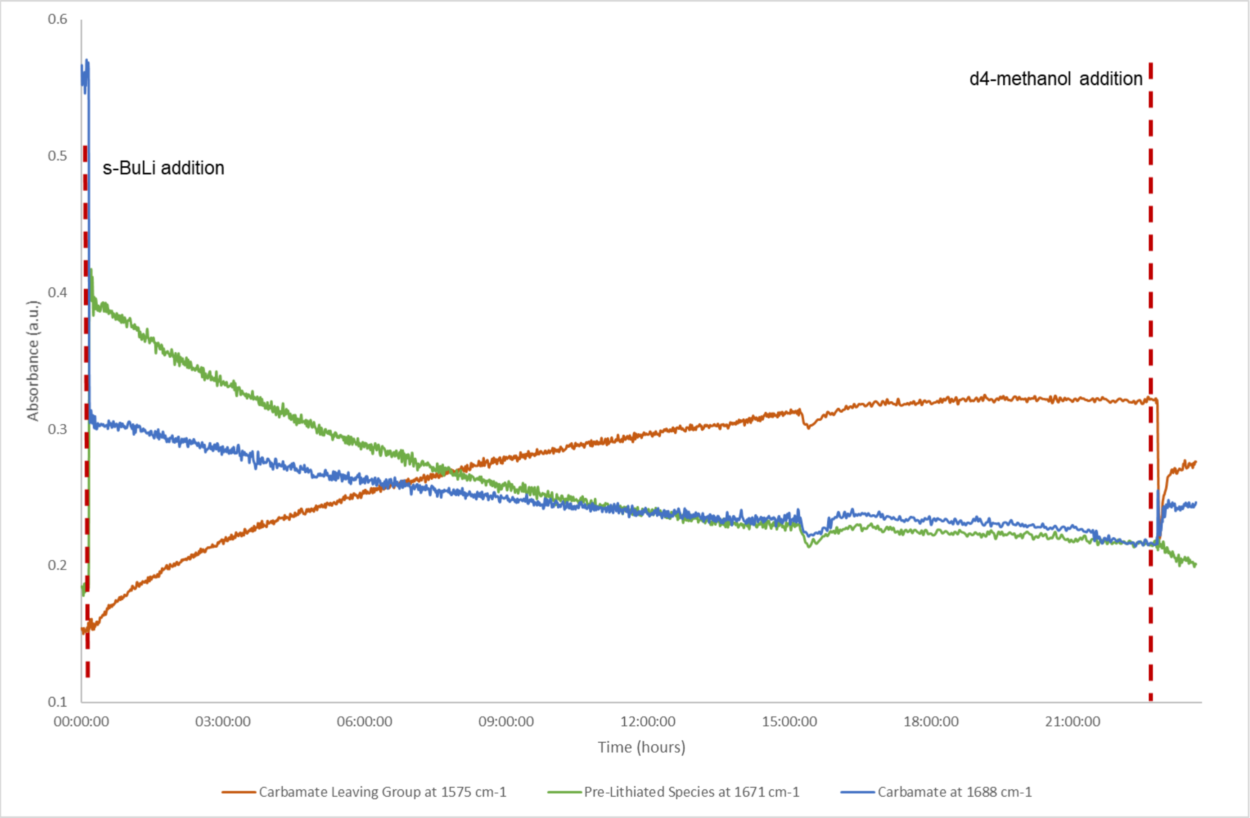

Trying to stabilise the cyclopentyl lithium carbenoid using the diisopropyl carbamate directing group (a worse leaving group than the TIB ester) failed. The "pre-lithiated complex" was observed, however this then decomposed to the carbamate anion without observation of the lithiated species. Addition of d_4-methanol led to poor recovery (32%) of non-deuterated (0% D incorporation) material.

Return to contents

4.7 Cyclohexyl TIB ester

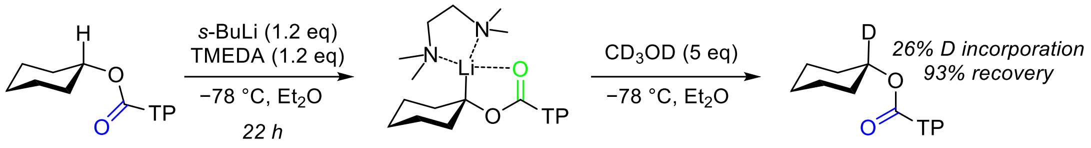

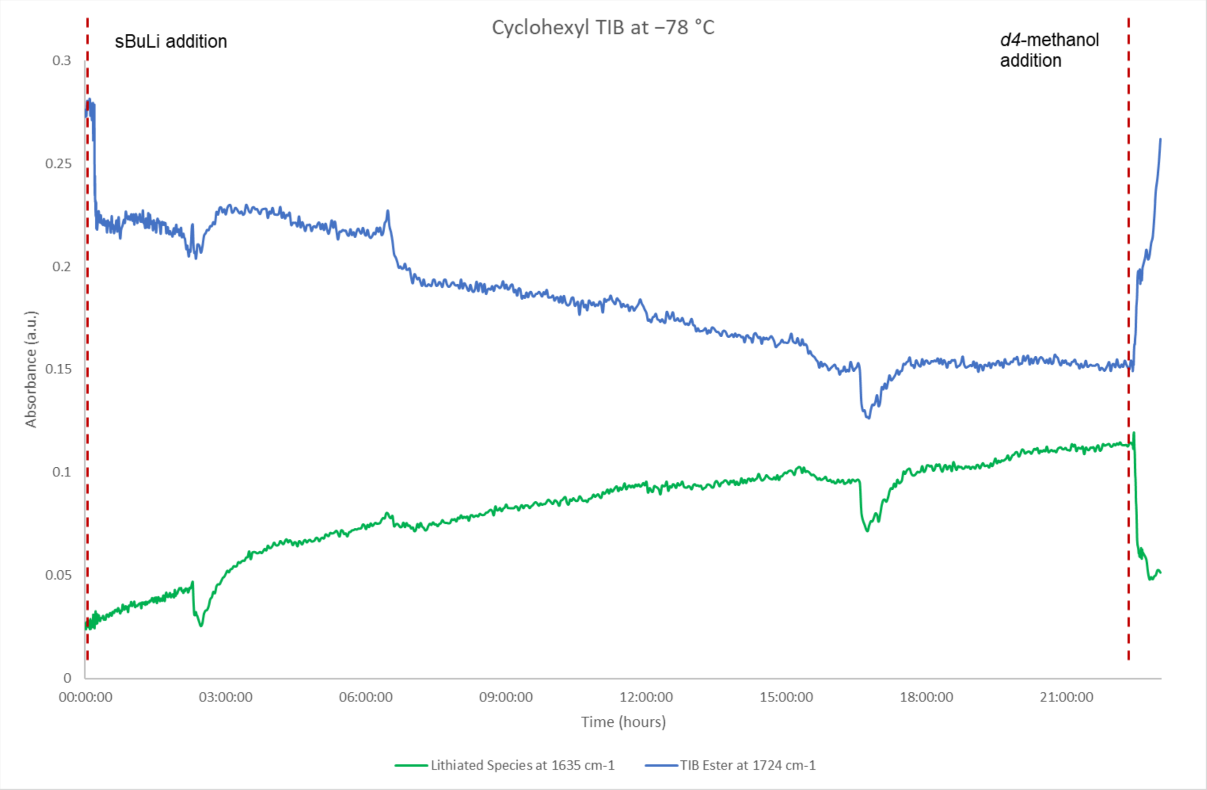

Lithiation of the cyclohexyl TIB ester is slow (22 h) and does not go to completion. Because of this, a boronic ester was not used and the reaction was simply quenched with *d*$_4$-methanol which gave 93% recovery with 26% D incorporation.

Return to contents

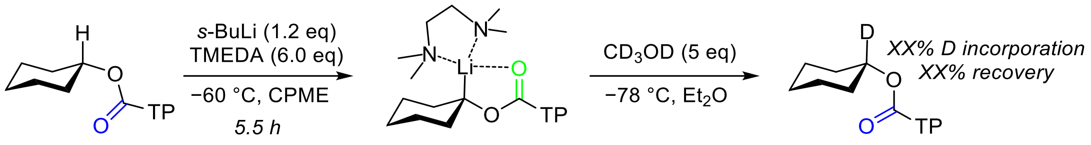

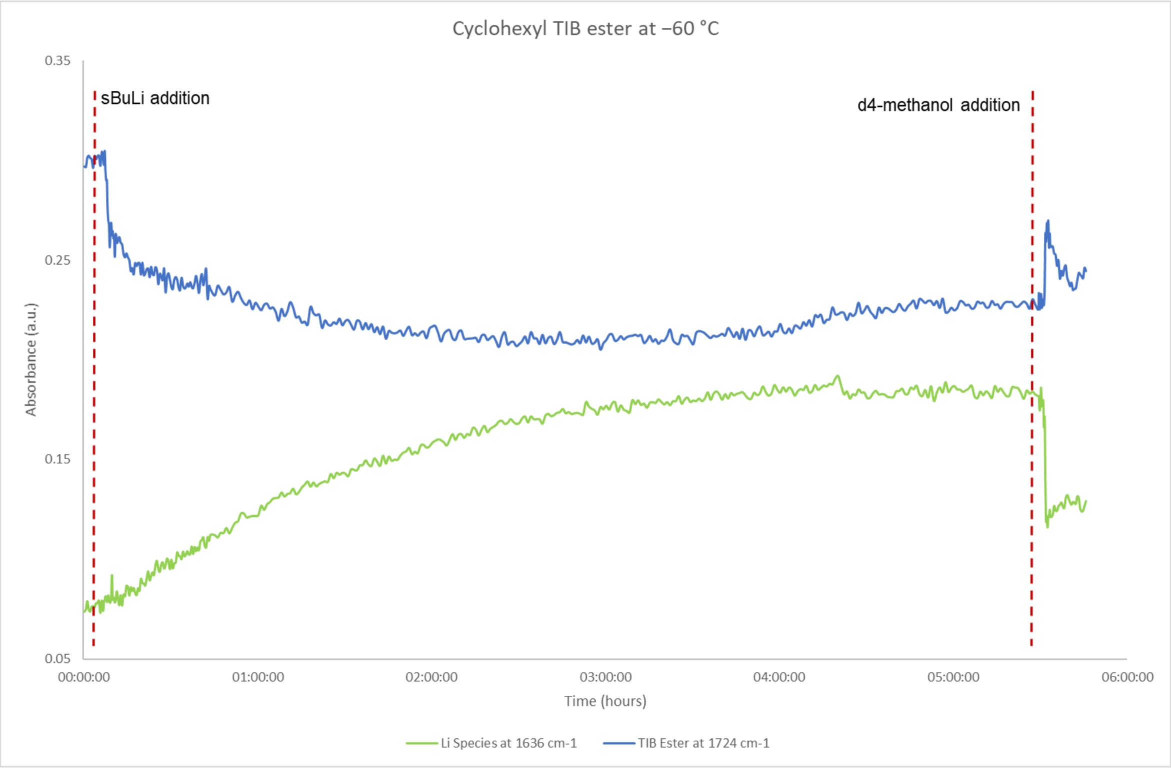

Trying to push the lithiation using conditions developed by Pulis *et al* for acyclic dialkyl benzoates (TMEDA (6.0 eq), −60 °C, CPME)[4] did not give any increase in deuterated material (95% recovery with 21% deuterium incorporation). The lithiation time was reduced to ~5 h.

4.8 In Situ IR spectroscopy 3D traces

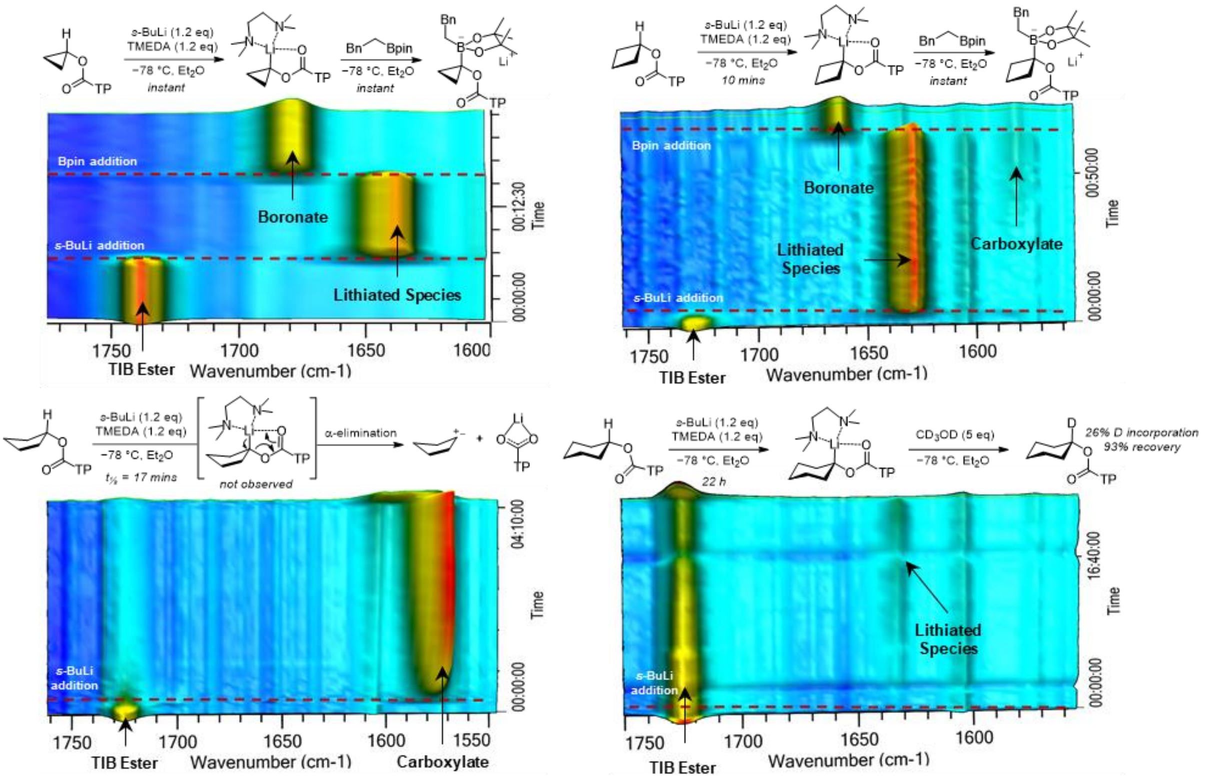

5 Attempts to use chloro/bromocyclobutane

All reactions performed on 0.25 mmol scale with respect to limiting reagent.

To a solution of halocyclobutane (1.3 eq, 0.33 mmol) and phenethylboronic acid pinacol ester (1.0 eq, 0.25 mmol) in the specified solvent (Et_2O or THF, ~0.3 M) at the specified temperature was added LiTMP/LDA (1.3 eq, 0.33 mmol, ~0.3 M) dropwise and the reaction stirred for the specified time (>1 h). After this the reaction was warmed to RT and stirred for >1 h at RT. Then the reaction mixture was filtered through a silica plug, the solvents removed under reduced pressure, and the crude mixture analysed using ^{1}H NMR.

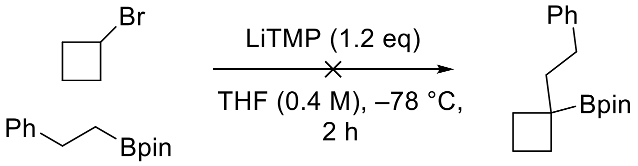

Bromocyclobutane not distilled. Recovered phenethylboronic acid pinacol ester 75%.

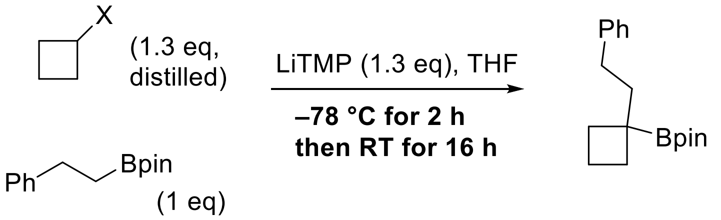

X = Br, 15% ^{1}H NMR yield
X = Cl, 7% ^{1}H NMR yield

Bromo/chlorocyclobutane distilled using Hickman flask (short path) immediately before use.

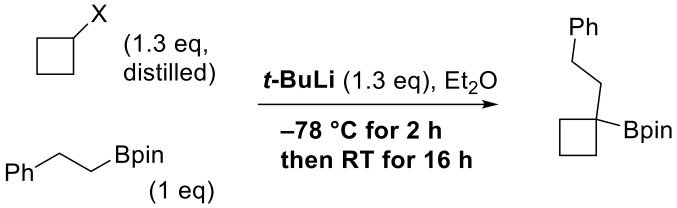

X = Cl/Br no product observed. White precipitate observed upon warming.

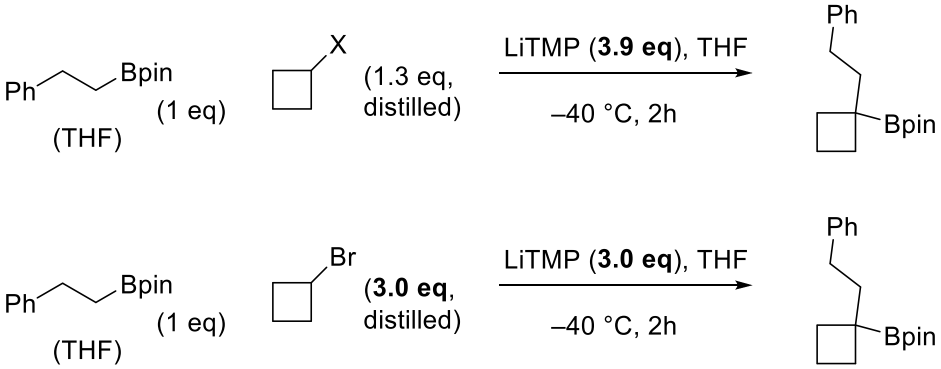

X = Br: ^{1}H NMR yield ~20%

X = Cl: ^{1}H NMR yield 9%

^{1}H NMR yield: 19%

Return to contents

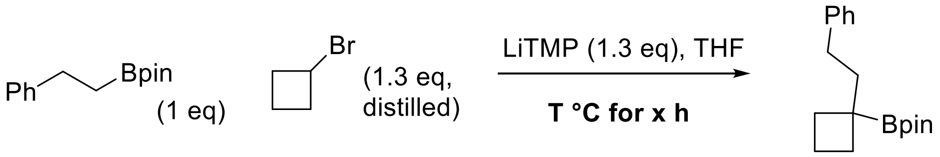

GC-MS all show both starting materials

T °C	x h	^{1}H NMR yield
–20	6	9%
–40	6	11%
–40	6	13%
–78	6	trace
0	6	9%
–20	16	15%

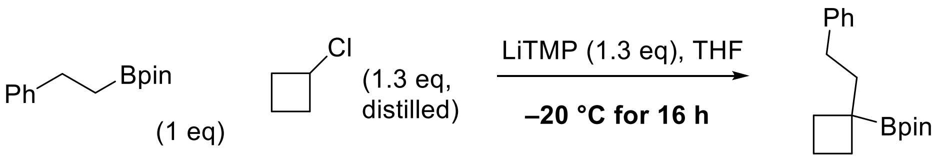

7% ^{1}H NMR yield

Due to consistently poor yields, this method was abandoned.

6 Lithiation-Borylation of cyclobutyl benzoate

6.1 General procedures

GP1

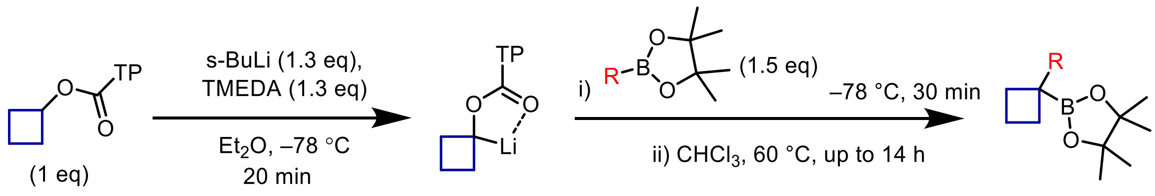

Under a nitrogen atmosphere, TMEDA (0.05 mL, 0.32 mmol, 1.3 eq), cyclobutyl 2,4,6-triisopropylbenzoate **2** (1.0 eq) and anhydrous Et_2O (0.2 M) were combined in a flame dried Schlenk tube. After cooling to −78 °C, *sec*-BuLi (1.3 M in cyclohexanes, 1.3 eq) was added dropwise and the brown/orange reaction stirred for 20 minutes. A solution of boronic ester (1.5 eq) in dry Et_2O (1 M), made up in a flame dried flask, was added dropwise to the reaction mixture. After ~30 minutes the reaction was warmed to room temperature and the solvent exchanged to $CHCl_3$ (~2.5 mL). The resultant mixture was heated to 60 °C for 16 hours or until full consumption of boronate complex was observed by ^{11}B NMR (typically 3 h). The crude material was filtered through a silica plug and washed with Et_2O, concentrated under reduced pressure, and purified by flash column chromatography using the gradient specified to afford the desired product.

GP2

Identical to GP1, however before column chromatography the boronic ester product can be oxidised for ease of purification:

The reaction mixture was cooled down to 0 °C and THF (0.50 mL) was added. Next, 2:1 NaOH (2 M):H_2O_2 (33% aq.) mixture (0 °C, 1 mL) was added dropwise. The reaction warmed to room temperature and stirred until complete by TLC analysis (typically 4–16 h). The reaction was dissolved in diethyl ether (10 mL) and washed with brine (5 mL). The aqueous phase was extracted twice with diethyl ether (5 mL). The combined organic phase was dried over $MgSO_4$ and evaporated to afford the crude alcohol, which was then purified by column chromatography.

Return to contents

6.2 Substrate Scope

Please use compound names to move between data and spectra.

6.2.1 Primary boronic esters

7 4,4,5,5-tetramethyl-2-(1-phenethylcyclobutyl)-1,3,2-dioxaborolane

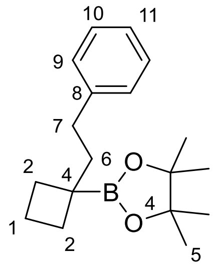

Compound **7** was synthesised following **GP1** using tert-butyl 4,4,5,5-tetramethyl-2-phenethyl-1,3,2-dioxaborolane (70 mg, 0.30 mmol, 1.50 eq) as the starting boronic ester. The crude mixture was purified using flash column chromatography (15-30% CH_2Cl_2/pentane) to afford the desired product (39 mg, 67%) as a colourless oil.

***R*f** = 0.30 (30% CH_2Cl_2/pentane, *p*-anisaldehyde);

^{1}H NMR (400 MHz, $CDCl_3$): *δ* = 7.31 – 7.13 (5H, m, **9 & 10 & 11**), 2.53 – 2.45 (2H, m, **7**), 2.21 – 2.12 (2H, m, **2**), 2.00 – 1.89 (2H, m, **1**), 1.89 – 1.82 (2H, m, **6**), 1.78 – 1.69 (2H, m, **2**), 1.29 (12H, s, **5**);

^{13}C NMR (101 MHz, $CDCl_3$): *δ* 143.4 (**8**), 128.5 (**9/10**), 128.4 (**9/10**), 125.6 (**11**), 83.2 (**4**), 42.2 (**6**), 33.4 (**7**), 30.6 (**2**), 24.9 (**5**), 18.4 (**1**);

Data in accordance with that reported in the literature.[5]

Return to contents

9 2-(1-(4-methoxybenzyl)cyclobutyl)-4,4,5,5-tetramethyl-1,3,2-dioxaborolane

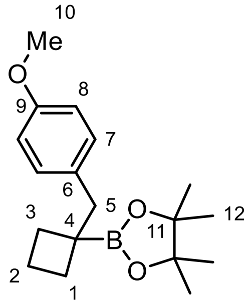

Compound **9** was synthesised following **GP1** using 2-(4-methoxybenzyl)-4,4,5,5-tetramethyl-1,3,2-dioxaborolane (74 mg, 0.30 mmol, 1.50 eq) as the starting boronic ester. The crude mixture was purified using flash column chromatography (5-10% Et_2O/petrol) to afford the desired product (41 mg, 67%) as a colourless oil.

***R**$_f$* = 0.30 (10% Et_2O/PE, *p*-anisaldehyde).

^{1}H NMR (400 MHz, $CDCl_3$): δ = 7.13 – 7.07 (2H, m, **9**), 6.79 – 6.75 (2H, m, **8**), 3.77 (3H, s, **11**), 2.80 (2H, s, **6**), 2.13 (2H, m, **2**), 1.96-1.82 (4H, m, **1 & 2**), 1.18 (12H, s, **5**).

^{13}C NMR (101 MHz, $CDCl_3$): δ = 157.8 (**10**), 133.5 (**7**), 130.1 (**9**), 113.4 (**8**), 83.2 (**4**), 55.4 (**11**), 44.2 (**6**), 30.4 (**2**), 24.8 (**5**), 18.3 (**1**).

HRMS (ESI+): $[M+Na]^+$ calcd for $C_{18}H_{27}BO_3Na$, 325.1949; found 325.1967.

IR (ν_{max}/cm^{-1}, neat): 2975, 2855, 1611, 1511, 1380, 1302, 1244, 1140, 1037, 836, 686, 631.

10 3-(1-(4,4,5,5-tetramethyl-1,3,2-dioxaborolan-2-yl)cyclobutyl)propanenitrile

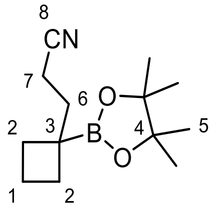

Compound **10** was synthesised following **GP1** 3-(4,4,5,5-tetramethyl-1,3,2-dioxaborolan-2-yl)propanenitrile (68 mg, 0.37 mmol, 1.50 eq) as the starting boronic ester. Purification via silica column chromatography (8-10% TBME/hexane), afforded

the desired product as a clear oil with minor impurities. NMR yield 69% (CH_2Br_2 as internal standard).

R*f* = 0.20 (8% TBME/hexane, $KMnO_4$)

^{1}H NMR (400 MHz, $CDCl_3$): *δ* = 2.25 (2H, m, **7**), 2.14 (2H, m, **2**), 2.02 (1H, m, **1**), 1.90 (3H, m, **1 & 6**), 1.72 (2H, m, **2**), 1.26 (12H, s, **5**).

^{13}C NMR (101 MHz, $CDCl_3$): *δ* = 120.4 (**8**), 83.4 (**4**), 35.2 (**6**), 29.9 (**2**), 24.7 (**5**), 17.9 (**1**), 14.2 (**7**).

HRMS-ESI (m/z): $[M – CH_3]^+$ calcd for $C_{12}H_{19}BNO_2$, 220.1503; found, 220.1503.

IR ($ν_{max}$/cm^{-1}, neat): 2977, 2930, 2858, 2245, 1388, 1314, 1214, 1143.

11 tert-Butyl 3-(1-(4,4,5,5-tetramethyl-1,3,2-dioxaborolan-2-yl)cyclobutyl)propanoate

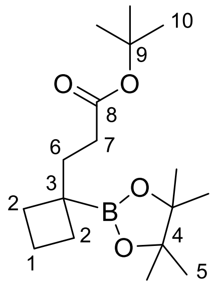

Compound **11** was synthesised following **GP1** using tert-butyl 3-(4,4,5,5-tetramethyl-1,3,2-dioxaborolan-2-yl)propanoate (96 mg, 0.37 mmol, 1.50 eq) as the starting boronic ester. Purification via silica column chromatography (10-15% Et_2O in pentane) afforded the desired product (49 mg, 68%) as a clear oil.

R*f* = 0.30 (10% Et_2O/pentane, $KMnO_4$);

^{1}H NMR (400 MHz, $CDCl_3$): 2.14 – 2.05 (m, 4H, **2 & 7**), 2.00 – 1.84 (m, 2H, **1**), 1.83 – 1.77 (m, 2H, **6**), 1.73 – 1.64 (m, 2H, **2**), 1.43 (9H, s, **10**), 1.25 (12H, s, **5**);

^{13}C NMR (101 MHz, $CDCl_3$): *δ* = 173.7 (**8**), 83.2 (**4**), 79.9 (**9**), 34.8 (**6**), 33.1 (**7**), 29.9 (**2**), 28.3 (**10**), 24.8 (**5**), 18.1 (**1**).

HRMS-EI (m/z): $[M - O^tBu]^+$ calcd for $C_{13}H_{22}O_3B$, 237.1662; found, 237.1657.

IR ($ν_{max}$/cm^{-1}, neat): 2976, 2932, 2858, 1728, 1386, 1368, 1307, 1140;

12 2-(1-(3-azidopropyl)cyclobutyl)-4,4,5,5-tetramethyl-1,3,2-dioxaborolane

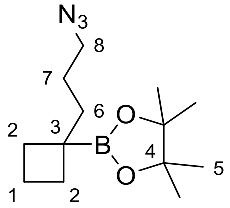

Compound **12** was synthesised following **GP2** using 2-(3-azidopropyl)-4,4,5,5-tetramethyl-1,3,2-dioxaborolane (59 mg, 0.30 mmol, 1.50 eq) as the starting boronic ester. The crude mixture was purified using flash column chromatography (20% to 30% CH_2Cl_2/pentane) to afford the desired product (13 mg, 25%) as a colourless oil.

R*f* = 0.27 (30% CH_2Cl_2/pentane);

^{1}H NMR (400 MHz, $CDCl_3$): *δ* = 3.22 (2H, t, *J* = 6.8 Hz, **8**), 2.17 – 2.07 (m, 2H, **2**), 2.01 – 1.81 (m, 2H, **2**), 1.68 (dt, *J* = 11.0, 8.9 Hz, 2H, **1**), 1.62 – 1.55 (m, 2H, **6**), 1.54 – 1.45 (m, 2H, **7**), 1.26 (s, 12H, **5**).

^{13}C HMR (101 MHz, $CDCl_3$): *δ* = 83.2 (**4**), 51.9 (**8**), 36.9 (**6**), 30.3 (**2**), 26.3 (**7**), 24.8 (**5**), 18.2 (**1**).

HRMS-ESI (m/z): $[M + Na]^+$ calcd for $C_{13}H_{24}BNaN_3O_2$, 288.1854; found, 288.1855.

IR (v_{max}/cm^{-1}, neat): 2976, 2093, 1387, 1309, 1143;

13 1-((4R)-4-((3R,8R,9S,10S,13R,14S,17R)-3-((tert-butyldimethylsilyl)oxy)-10,13-dimethylhexadecahydro-1H-cyclopenta[a]phenanthren-17-yl)pentyl)cyclobutan-1-ol (Lithocholic acid derivative)

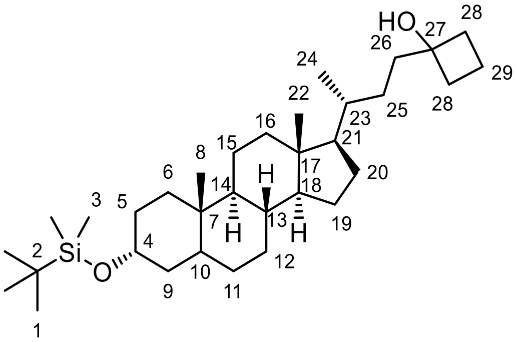

Compound **13** was synthesised following **GP2** using tert-butyl(((3R,8R,9S,10S,13R,14S,17R)-10,13-dimethyl-17-((R)-5-(4,4,5,5-tetramethyl-1,3,2-dioxaborolan-2-yl)pentan-2-yl)hexadecahydro-1H-cyclopenta[a]phenanthren-3-

yl)oxy)dimethylsilane (117 mg, 0.37 mmol, 1.50 eq) as the starting boronic ester. Purification via silica column chromatography (15-20% Et_2O/pentane) gave the desired product (81 mg, 63%) as a clear oil.

R*f* = 0.25 (15% Et_2O/pentane, $KMnO_4$).

^{1}H NMR (400 MHz, $CDCl_3$): *δ* = 3.57 (ddd, J = 15.3, 10.5, 4.5 Hz, 1H, **4**), 2.08 – 0.99 (m, 35H), 0.93 (d, J = 6.4 Hz, 3H, **24**), 0.90 (s, 3H, **8/22**), 0.89 (s, 9H, **1**), 0.64 (s, 3H, **8/22**), 0.05 (s, 6H, **3**).

^{13}C NMR (101 MHz, $CDCl_3$): *δ* = 75.7 (**27**), 73.0 (**4**), 56.6 (**CH**), 56.2 (**21**), 42.8 (**CH**), 42.3 (**CH**), 40.4 (**CH_2**), 40.3 (**CH**), 37.1 (**CH_2**), 36.1 & 36.0 & 36.0 & 36.0 & 36.0 & 35.9 (**3xCH_2 & 2xCH & C**), 34.7 (**C**), 31.2 (**CH_2**), 29.3 (**CH_2**), 28.4 (**CH_2**), 27.5 (**CH_2**), 26.6 (**CH_2**), 26.1 (**1**), 24.4 (**CH_2**), 23.6 (**8/22**), 20.8 (**CH_2**), 19.0 (**24**), 18.5 (**2**) 12.3 (**CH_2**), 12.2 (**8/22**), −4.4 (**3**).

HRMS-MALDI (m/z): $[M + Na]^+$ calcd for $C_{33}H_{60}NaO_2Si$, 539.4260; found, 539.4269.

IR (ν_{max}/ cm^{-1}, neat): 3358, 2927, 2856, 1462, 1448, 1373, 1251, 1094, 1077, 834, 733;

Return to contents

6.2.2 Secondary boronic esters

14 2-(1-cyclohexylcyclobutyl)-4,4,5,5-tetramethyl-1,3,2-dioxaborolane

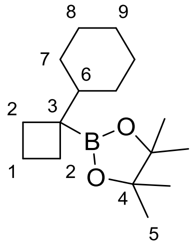

Compound **14** was synthesised following GP1 using 2-cyclohexyl-4,4,5,5-tetramethyl-1,3,2-dioxaborolane (104 mg, 0.50 mmol, 1.50 eq) as the starting boronic ester. The crude mixture was purified using flash column chromatography (10% – 50 % CH_2Cl_2 /pentane) to afford the desired product (49 mg, 60%) as a colourless oil.

R_f = 0.25 (20% CH_2Cl_2/pentane).

^{1}H NMR (400 MHz, $CDCl_3$): δ = 2.12 – 2.01 (m, 2H, **2**), 1.88 – 1.59 (m, 10H, **7$_{eq}$ & 2 & 9 & 8**), 1.27 (s, 13H, **5 & 6**), 1.23 – 1.03 (m, 2H, **8**), 0.91 (qd, *J* = 13.0, 3.6 Hz, 1H, **7$_{ax}$**).

^{13}C NMR (101 MHz, $CDCl_3$): δ = 82.8 (**4**), 48.2 (**6**), 29.4 (**2**), 29.0 (**7**), 26.9 (**9**), 26.8 (**8**), 24.8 (**5**), 18.0 (**1**).

HRMS-ESI (m/z): $[M + Na]^+$ calcd for $C_{16}H_{29}BNaO_2$, 287.2153; found, 287.2157.

IR (ν_{max}/cm^{-1}, neat) ; 2976, 2922, 2851, 1446, 1381, 1141.

15 1-(1-phenylethyl)cyclobutan-1-ol

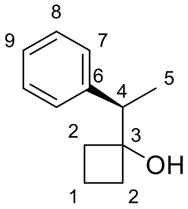

Compound **15** was synthesised following **GP2** using 4,4,5,5-tetramethyl-2-(1-phenylethyl)-1,3,2-dioxaborolane (70 mg, 0.30 mmol, 1.50 eq.). The crude mixture was purified using flash column chromatography (5-10% Et_2O/ pentane) to afford the desired product (19 mg, 56%) as a colourless oil.

R_f = 0.20 (10% Et_2O/pentane).

^{1}H NMR (400 MHz, $CDCl_3$) δ = 7.35 – 7.27 (4H, m, **7 & 8**), 7.25 – 7.21 (1H, m, **9**), 2.93 (1H, q, J = 7.2 Hz, **4**), 2.29 – 2.20 (m, 1H, **1/2**), 2.18 – 2.10 (m, 1H, 1/2), 2.07 – 1.98 (m, 1H, **1/2**), 1.86 (dddd, J = 14.2, 10.2, 9.5, 4.9 Hz, 1H, **1/2**), 1.80 – 1.71 (m, 1H, **1/2**), 1.67 – 1.51 (m, 1H, **1/2**), 1.32 (d, J = 7.2 Hz, 3H, **5**).

^{13}C NMR (101 MHz, $CDCl_3$) δ = 142.7 (**6**), 128.7 (**7/8/9**), 128.4 (**7/8/9**), 126.7 (**7/8/9**), 78.1 (**3**), 47.2 (**4**), 34.6 (**2**), 34.3 (**2**), 14.4 (**5**), 12.6 (**1**).

HRMS-ESI (m/z): [M + Na]+ calcd. for $C_{12}H_{16}NaO_2$, 199.1099; found, 199.1090.

IR ν_{max}/cm^{-1} 2968 (broad), 1455, 1260, 1922, 799, 702.

Chiral HPLC (IB with guard, 1% *i*PrOH/hexane, 1.2 mL min^{-1}, RT) T_R 6.01 min (major), 6.27 (minor).

$[\alpha]_D^{24}$ +12.2 (*c* 0.49, $CHCl_3$).

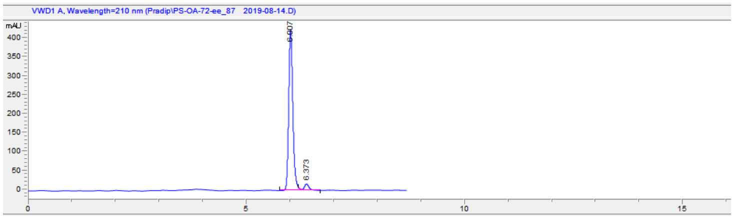

#	Time	Type	Area	Height	Width	Area%	Symmetry
1	6.007	BV R	2821.6	424.7	0.1015	95.591	0.794
2	6.373	VB E	130.1	17.4	0.1121	4.409	0.778

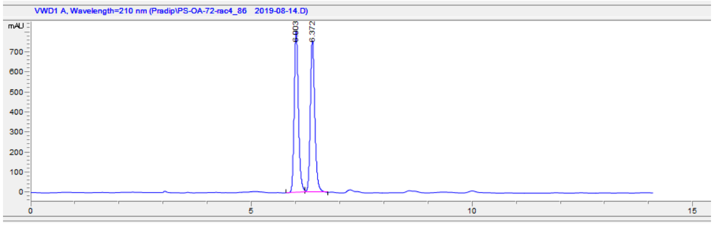

#	Time	Type	Area	Height	Width	Area%	Symmetry
1	6.003	BV	5440.4	811.9	0.1026	50.073	0.794
2	6.372	VB	5424.6	764.8	0.109	49.927	0.801

Return to contents

16 (*R*)-2-(1-(4-(4-methoxyphenyl)butan-2-yl)cyclobutyl)-4,4,5,5-tetramethyl-1,3,2-dioxaborolane

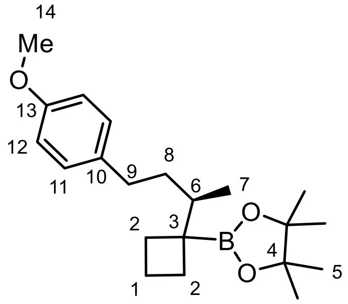

Compound **16** was synthesised following **GP1** using (*R*)-2-(4-(4-methoxyphenyl)butan-2-yl)-4,4,5,5-tetramethyl-1,3,2-dioxaborolane (143.7 mg, 0.5 mmol, 1.5 eq) followed by column chromatography purification (3% to 8% Et_2O in petrol) to obtain a colourless oil (61 mg, 54% yield).

R_f = 0.38 (10% Et_2O/PE, *p*-anisaldehyde);

^{1}H NMR (400 MHz, $CDCl_3$): δ = 7.13 – 7.05 (2H, m, **11**), 6.85 – 6.78 (2H, m, **12**), 3.79 (3H, s, **15**), 2.64 (1H, ddd, J = 14.8, 10.2, 5.0 Hz, **9**), 2.42 (1H, ddd, J = 13.8, 10.2, 6.8 Hz, **9**), 2.08 (2H, dt, J = 7.8, 5.3 Hz, **2**), 1.85 – 1.76 (1H, m, **1**), 1.76 – 1.61 (m, 4H, **2 & 1 & 8**, 1.52 – 1.43 (m, 1H, **5**), 1.36 – 1.28 (m, 1H, **8**), 1.25 (12H, s, **5**), 0.89 (3H, d, J = 6.7 Hz, **7**);

^{13}C NMR (101 MHz, $CDCl_3$): δ = 157.7 (**13**), 135.5 (**10**), 129.4 (**11**), 113.8 (**12**), 83.0 (**4**), 55.4 (**14**), 43.2 (**6**), 35.7 (**8**), 33.6 (**9**), 30.7 (**2**), 30.2 (**2**), 24.9 (**5**), 17.9 (**1**), 15.6 (**7**);

HRMS (ESI+): calcd. for $C_{21}H_{33}BO_3Na$ $[M+Na]^+$: 367.2420; found 367.2433;

IR (ν_{max}/ cm^{-1}, neat): 2956, 1512, 1386, 1201, 1244, 1143, 1038, 843;

HPLC & optical rotation analysis performed after oxidation following GP2.

Chiral HPLC (IB with guard, 3% *i*PrOH/hexane, 0.7 ml/min, RT) T_R 16.84 min (major), 18.65 (minor);

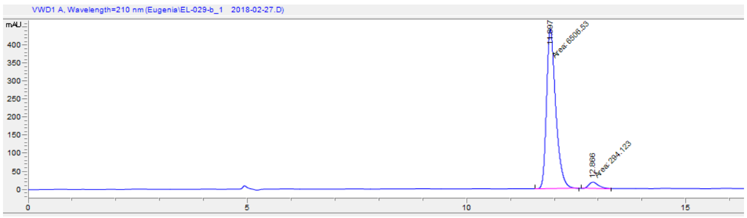

#	Time	Type	Area	Height	Width	Area%	Symmetry
1	11.897	MM	6506.5	444.7	0.2439	95.675	0.675
2	12.866	MM	294.1	18.4	0.2663	4.325	0.718

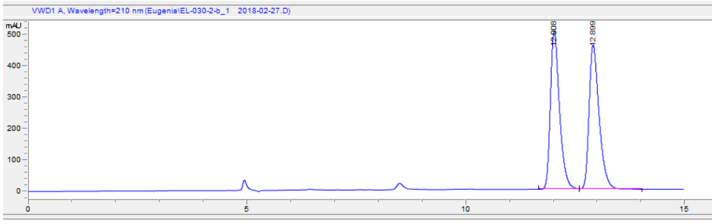

#	Time	Type	Area	Height	Width	Area%	Symmetry
1	12.008	BV	7332.3	506.8	0.2176	49.897	0.656
2	12.899	VV R	7362.6	460.8	0.2407	50.103	0.627

$[\alpha]_D^{20}$ +2.34 (*c* 1.9, $CHCl_3$).

17 4,4,5,5-tetramethyl-2-(*trans*-2-phenylcyclopropyl)cyclobutyl)-1,3,2-dioxaborolane

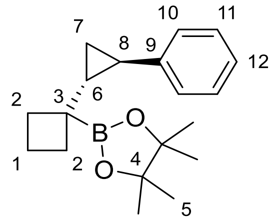

(+/–)

Compound **17** was synthesised following **GP1** using 4,4,5,5-tetramethyl-2-(*trans*-2-phenylcyclopropyl)-1,3,2-dioxaborolane (73 mg, 0.30 mmol, 1.50 equiv.) as the starting boronic ester. The crude mixture was purified using flash column chromatography (5-10% CH_2Cl_2/pentane), to afford the desired product (38 mg, 65%) as a colourless oil.

R*f* = 0.45 (30% CH_2Cl_2/pentane, *p*-anisaldehyde).

^{1}H NMR (400 MHz, $CDCl_3$): *δ* 7.25–7.20 (2H, m, **11**), 7.14–7.07 (3H, m, **10 & 12**), 2.11–2.02 (2H, m, **2**), 1.91–1.73 (4H, m, **2 & 1**), 1.29-1.22 (2H, m, **6 & 8**), 1.26 (6H, s, **5**), 1.25 (6H, s, **5**), 0.97 (1H, ddd, *J* = 8.8, 6.0, 4.8 Hz, **7**), 0.85 (1H, dt, *J* = 8.8, 5.1 Hz, **7**).

^{13}C NMR (101 MHz, $CDCl_3$): *δ* 144.2 (**9**), 128.2 (**11**), 126.2 (**12**), 125.1 (**10**), 83.3 (**4**), 29.5 (**8**), 28.1 (**2**), 27.9 (**2**), 24.8 (**5**), 19.7 (**1**), 18.3 (**6**), 12.5 (**7**).

HRMS-ESI (m/z): [M + Na]+ calcd. for $C_{19}H_{27}BNaO_2$, 322.1996; found, 322.2007.

IR (ν_{max}/cm^{-1}, neat): 2975, 1371, 1308, 1144, 1127, 696.

18 tert-butyl 2-(1-(4,4,5,5-tetramethyl-1,3,2-dioxaborolan-2-yl)cyclobutyl)pyrrolidine-1-carboxylate

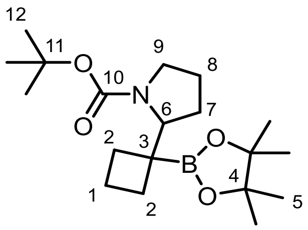

Compound **18** was synthesised following **GP1** using tert-butyl 2-(4,4,5,5-tetramethyl-1,3,2-dioxaborolan-2-yl)pyrrolidine-1-carboxylate (111 mg, 0.30 mmol, 1.50 equiv.). The crude mixture was purified using flash column chromatography (5-15% Et_2O/pentane), to afford the desired product (38 mg, 42%) as a colourless oil.

R_f = 0.13 (10% Et_2O/pentane);

^{1}H NMR (500 MHz, $CDCl_3$): δ = 3.89 (br s, 1H, **6**), 3.60 & 3.48 (2×br s, 1H, **9**), 3.24 (br s, 1H, **9**), 2.15 – 1.73 (m, 7H, **7, 8 & 2**), 1.70 – 1.54 (m, 3H, **2 & 1**), 1.45 (s, 9H, **12**), 1.24 (s, 12H, **5**).

^{13}C NMR (126 MHz, $CDCl_3$): δ = 155.8 & 155.5 (**10**), 83.0 & 82.5 (**4**), 79.2 & 79.0 (**11**), 63.2 & 62.3 (**6**), 47.9 (**9**), 30.7 & 30.2 (**2/7/8**), 28.5 (**12**), 26.2 (**2/7/8**), 24.8 (**5**), 24.3 & 23.9 (**2/7/8**), 18.3 &18.1 (**1**).

Compound exists as 1:1 mixture of rotamers at room temperature.

HRMS-ESI (m/z): $[M + H]^+$ calcd. for $C_{19}H_{35}BNO_4$, 352.2654; found 352.2653.

IR (ν_{max}/cm^{-1}, neat): 3675, 2973, 1687, 1381, 1260, 1066, 868, 764,

Return to contents

19 tert-butyl 4-(1-(4,4,5,5-tetramethyl-1,3,2-dioxaborolan-2-yl)cyclobutyl)piperidine-1-carboxylate

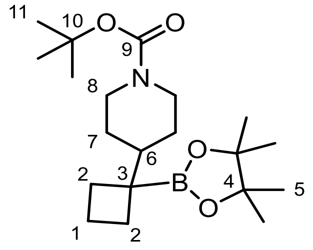

Compound **19** was synthesised following **GP1** using tert-butyl 4-(4,4,5,5-tetramethyl-1,3,2-dioxaborolan-2-yl)piperidine-1-carboxylate (111 mg, 0.36 mmol, 1.5 equiv.) as the starting boronic ester. Purification via silica column chromatography, eluting with 5-15% Et_2O/ pentane, afforded the title compound (42 mg, 48%) as a clear oil.

$\boldsymbol{R_f}$ = 0.64 (15% Et_2O/pentane, anisaldehyde);

^{1}H NMR (400 MHz, $CDCl_3$): δ = 4.17 (br s, 2H, **8**), 2.58 (t, J = 11.1 Hz, 2H, **8**), 2.08 (t, J = 7.7 Hz, 2H, **2**), 1.93-1.81 (m, 1H, **1**), 1.81 – 1.69 (m, 3H, **1 & 2**), 1.56 (br d, J = 13.7 Hz, 2H, **7**), 1.44 (s, 9H, **12**), 1.44 (m, 1H, **6**), 1.24 (s, 12H, **5**), 1.17 (m, 2H, **7**) ppm;

^{13}C NMR (101 MHz, $CDCl_3$): δ = 155.2 (**10**), 83.2 (**4**), 79.2 (**5**), 46.4 (**6**), 44.9 (**8**), 29.1 (**2**), 28.6 (**11**), 28.2 (**7**), 24.9 (**5**), 18.1 (**2**). **C8** – *Carbon broadened by nitrogen quadrupolar relaxation/presence of rotamers;*

HRMS (ESI+): $[M+H]^+$ calcd. for $C_{20}H_{37}BNO_4$, 366.2810; found, 366.2804.

IR (ν_{max}/cm^{-1}, neat): 3675, 2975, 2930, 1693, 1466, 1364, 1275, 1142, 966, 868, 764;

20 2-(1-((1*R*,2*S*,5*R*)-2-isopropyl-5-methylcyclohexyl)cyclobutyl)-5,5-dimethyl-1,3,2-dioxaborinane

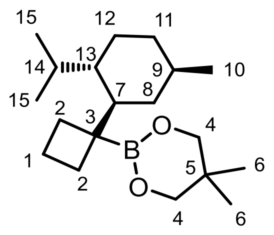

Compound **20** was synthesised following GP1 using 2-((2R,5R)-2-isopropyl-5-methylcyclohexyl)-5,5-dimethyl-1,3,2-dioxaborinane (100 mg, 0.40 mmol, 1.50 equiv.) as the starting boronic ester. The crude mixture was purified using flash column

chromatography (0% – 15% CH_2Cl_2/pentane) to afford the title compound as an amorphous white solid (46 mg, 58%).

R*f* = 0.6 (20% CH_2Cl_2/pentane);

^{1}H NMR (400 MHz, $CDCl_3$) *δ* ppm; 3.63 (s, 4H, **4**), 2.21 – 2.13 (m, 1H, **C*H*H**), 2.07 – 1.92 (m, 2H, **14 & C*H*H**), 1.85 (q, J = 9.7 Hz, 1H, **C*H*H**), 1.77 – 1.58 (m, 5H, **8 & CH₂ & 2×C*H*H**), 1.57 – 1.49 (m, 1H, **11**), 1.32 (td, J = 11.4, 10.8, 2.7 Hz, 1H, **7**), 1.27 – 1.16 (m, 1H, **9**), 0.99 (s, 6H, **6**), 0.96 – 0.88 (m, 2H, **13 & 11**), 0.85 (d, J = 9.8 Hz, 3H, **10**), 0.83 (d, J = 10.1 Hz, 3H, **15**), 0.79 – 0.74 (m, 1H, **C*H*H**), 0.72 (d, J = 6.8 Hz, 3H, **15**), 0.68 (q, J = 12.4 Hz, 1H, **8**).

^{13}C NMR (101 MHz, $CDCl_3$) *δ* ppm; 72.1 (**4**), 51.6 (**7**), 49.1 (**13**), 38.2 (**8**), 35.9 (**CH_2**), 35.0 (**CH_2**), 33.6 (**9**), 31.8 (**CH_2**), 31.6 (**5**), 27.3 (**14**), 25.1 (**11**), 23.1 (**10**), 22.2 (**6/15**), 22.2 (**6/15**), 19.2 (**CH_2**), 16.3 (**15**).

HRMS-APCI (m/z): $[M+H]^+$ calcd for $C_{19}H_{35}O_2B$, 307.2803; found, 307.2806.

IR (v_{max}/cm^{-1}, neat): 2952, 2940, 2918, 2884, 1409, 1350, 1229, 1165, 1090.

21 tert-butyl 2-(1-(4,4,5,5-tetramethyl-1,3,2-dioxaborolan-2-yl)cyclobutyl)piperidine-1-carboxylate

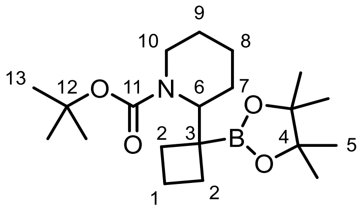

Compound **21** was synthesised following **GP1** using *tert*-butyl 2-(4,4,5,5-tetramethyl-1,3,2-dioxaborolan-2-yl)piperidine-1-carboxylate (115 mg, 0.37 mmol, 1.5 eq) as the starting boronic ester, with a borylation time of 30 minutes. Purification via silica column chromatography, eluting with 10-15% Et_2O in pentane, afforded the title compound (42 mg, 46%) as a clear oil.

***R*f** = 0.38 (10% Et_2O/PE, *p*-anisaldehyde);

^{1}H NMR (400 MHz, $CDCl_3$): *δ* = 4.03 (br s, 1H, **6/10**), 3.79 (br s, 1H, **6/10**), 3.09 (br s, 1H, **6/10**), 2.11 – 1.93 (m, 3H, **2 & 7/8/9**), 1.90 – 1.70 (m, 3H, **2 & 1**), 1.69 – 1.52 (m, 2H, **7/8/9**), 1.43 (s, 9H, **13**), 1.47 – 1.39 (m, 2H, **7/8/9**) 1.24 (s, 12H, **5**).

^{13}C NMR (101 MHz, $CDCl_3$): δ = 155.9 (**11**), 82.9 (**4**), 79.3 (**12**), 29.9 (**2**), 29.2 (**7/8/9**), 28.6 (**13**), 25.8 (**7/8/9**), 24.9 (**5 & 7/8/9**), 20.9 (**6/10**), 18.4 (**1**).

HRMS (ESI+): calcd. for $C_{20}H_{37}BNO_4Na$ $[M+H]^+$: 366.2810; found, 366.2812;

IR (ν_{max}/cm^{-1}, neat): 2979, 2924, 2830, 1683, 1368, 1232, 1143, 1025, 867.

Return to contents

6.2.3 sp^2 boronic esters

22 4,4,5,5-tetramethyl-2-(1-phenylcyclobutyl)-1,3,2-dioxaborolane

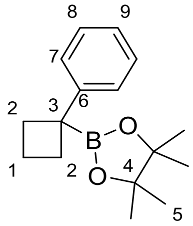

Compound **22** was synthesised following **GP1** using 4,4,5,5-tetramethyl-2-phenyl-1,3,2-dioxaborolane (77 mg, 0.30 mmol, 1.50 equiv.) as the starting boronic ester. The crude mixture was purified using flash column chromatography with excess silica (40% CH_2Cl_2/pentane) to afford the title compound as a colourless oil (34 mg, 64%).

R*f* = 0.2 (40% CH_2Cl_2/pentane).

^{1}H NMR (400 MHz, $CDCl_3$): *δ* 7.26 (2H, m, **8**), 7.23-7.10 (3H, m, **7 & 9**), 2.54 (tt, *J* = 9.0, 2.7, Hz, **2**), 2.28 (2H, m, **2**), 2.04 (1H, m, **1**), 1.84 (1H, dtt, *J* = 10.2, 8.5, 2.7 Hz, **1**), 1.19 (12H, s, **5**).

^{13}C NMR (101 MHz, $CDCl_3$): *δ* 149.1 (**6**), 128.1 (**8**), 125.7 (**7**), 124.5 (**9**), 83.6 (**4**), 32.1 (**2**), 24.6 (**5**), 19.0 (**1**).

HRMS-ESI (m/z): $[M + Na]^+$ calcd for $C_{16}H_{23}BNaO_2$, 281.1686; found, 281.1694.

IR (ν_{max}/cm^{-1}, neat): 2976, 1722, 1686, 1598, 1515, 1351, 1145, 698.

23 1-(4-methoxyphenyl)cyclobutan-1-ol

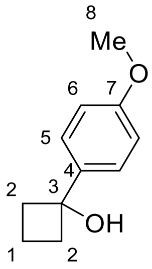

Compound **23** was synthesised following **GP2** using 2-(4-methoxyphenyl)-4,4,5,5-tetramethyl-1,3,2-dioxaborolane (88 mg, 0.3 mmol, 1.5 equiv.) as the starting boronic ester. The crude mixture was purified using flash column chromatography (10-20% EtOAc/pentane) to afford **23** as a amorphous white solid (26 mg, 66%).

Rf = 0.25 (% Et_2O/pentane).

^{1}H NMR (400 MHz, $CDCl_3$) δ = 7.42 (d, J = 8.8 Hz, 2H, **5**), 6.90 (d, J = 8.8 Hz, 2H, **6**), 3.81 (s, 3H, **8**), 2.59 – 2.48 (m, 2H, **2**), 2.41 – 2.30 (m, 2H, **2**), 1.97 (dtt, J = 11.1, 9.6, 5.0 Hz, 1H, **1**), 1.72 – 1.56 (m, 1H, **1**).

^{13}C NMR (101 MHz, $CDCl_3$) δ = 158.8 (**7**), 138.6 (**4**), 126.5 (**5**), 113.8 (**6**), 76.8 (**3**), 55.4 (**8**), 37.0 (**2**), 13.0 (**1**).

Spectroscopic information matched with literature.[6]

24 2-(1-(4-chlorophenyl)cyclobutyl)-4,4,5,5-tetramethyl-1,3,2-dioxaborolane

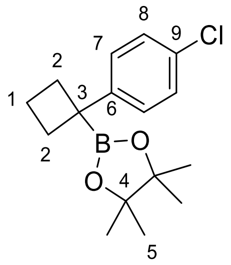

Compound **24** was synthesised following **GP1** using 2-(4-chlorophenyl)-4,4,5,5-tetramethyl-1,3,2-dioxaborolane (77.4 mg, 0.325 mmol, 1.3 equiv.) as the starting boronic ester. Purification via silica column chromatography, eluting with 30% CH_2Cl_2 in pentane, afforded the title compound (37 mg, 52%) as a colourless oil.

***R*$_f$** = 0.30 (30% CH_2Cl_2 in pentane, anisaldehyde).

^{1}H NMR (400 MHz, $CDCl_3$): δ = 7.21 (d, J = 8.6 Hz, 2H, **8**), 7.03 (d, J = 8.7 Hz, 2H, **7**), 2.53 (tt, J = 8.3, 2.6 Hz, 2H, **2**), 2.27 – 2.17 (m, 2H, **2**), 2.10 – 1.97 (m, 1H, **1**), 1.88 – 1.79 (m, 1H, **1**), 1.19 (s, 12H, **5**).

^{13}C NMR (101 MHz, $CDCl_3$): δ = 147.5 (**6**), 130.1 (**9**), 128.0 (**8**), 127.0 (**7**), 83.5 (**4**), 32.0 (**2**), 24.5 (**5**), 18.7 (**1**).

HRMS-ESI (m/z): $[M+Na]^+$ calcd for $C_{16}H_{22}BClNaO_2$, 315.1300; found, 315.1297.

IR (v_{max}/ cm^{-1}, neat): 2976.3, 2933.7, 2869.0, 1350.3, 1314.2, 1119.6, 1091.3, 844.6.

25 *tert*-butyl 4-(1-(4,4,5,5-tetramethyl-1,3,2-dioxaborolan-2-yl)cyclobutyl)-3,6-dihydropyridine-1(2*H*)-carboxylate

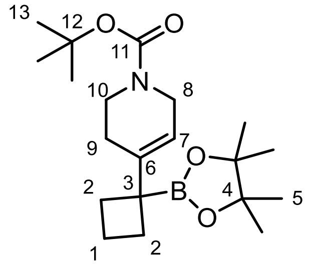

Compound **25** was synthesised following **GP1** using tert-butyl 4-(4,4,5,5-tetramethyl-1,3,2-dioxaborolan-2-yl)-3,6-dihydropyridine-1(2H)-carboxylate (117 mg, 0.38 mmol, 1.5 equiv.) as the starting boronic ester. Purification via silica column chromatography, eluting with 5-10% EtOAc/ pentane, afforded the title compound (58 mg, 62%) as a clear oil.

***R*f** = 0.38 (30% Et_2O/ pentane, anisaldehyde).

^{1}H NMR (500 MHz, $CDCl_3$): δ = 5.21 (br s, 1H, **7**), 3.88 (br s, 2H, **8/10**), 3.43 (br t, J = 5.9 Hz, 2H, **8/10**), 2.18 (tt, J = 7.2, 2.5 Hz, 2H, **2**), 2.03 – 1.94 (m, 4H, **2 & 9**), 1.94 – 1.85 (m, 1H, **1**), 1.75 – 1.68 (m, 1H, **1**), 1.46 (s, 9H, **13**), 1.24 (s, 12H, **5**).

^{13}C NMR (126 MHz, $CDCl_3$): δ = 155.1 (**11**), 142.0 (**6**), 114.6 & 114.0 (**7**), 83.3 (**4**), 79.2 (**12**), 43.8 & 43.2 (**8/10**), 41.2 & 40.0 (**8/10**), 29.6 (**2**), 28.5 (**13**), 26.0 (**9**), 24.6 (**5**), 18.0 (**1**). *Some peaks are doubled due to the presence of rotamers.*

HRMS-ESI (m/z): [M+Na]+ calcd for $C_{20}H_{34}BNNaO_4$, 386.2477; found, 386.2476.

IR (ν_{max}/ cm^{-1}, neat): 3675, 2973, 2901, 1693, 1394, 1260, 1066.

26 2-(1-(benzofuran-2-yl)cyclobutyl)-4,4,5,5-tetramethyl-1,3,2-dioxaborolane

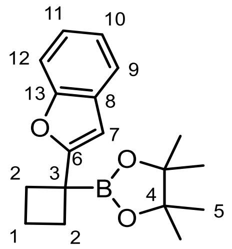

Compound **26** was synthesised following **GP1** using 2-(benzofuran-2-yl)-4,4,5,5-tetramethyl-1,3,2-dioxaborolane (92 mg, 0.38 mmol, 1.50 equiv.) as the starting

boronic ester. Purification via silica column chromatography, eluting with 5% to 10% Et_2O/pentane, afforded the title compound (39 mg, 53%) as a colourless oil.

***R*f** = 0.50 (10% Et_2O/pentane, *p*-anisaldehyde).

^{1}H NMR (400 MHz, $CDCl_3$): *δ* = 7.49 – 7.46 (m, 1H, **9**), 7.43 – 7.39 (m, 1H, **12**), 7.20 – 7.13 (m, 2H, **10 & 11**), 6.44 (d, J = 0.9 Hz, 1H, **7**), 2.55 – 2.42 (m, 4H, **2**), 2.17 – 2.00 (m, 2H, **1**), 1.30 (s, 12H, **5**).

^{13}C NMR (101 MHz, $CDCl_3$): *δ* = 164.1 (**6**), 155.0 (**13**), 129.5 (**8**), 122.9 (**10/11**), 122.3 (**10/11**), 120.3 (**9**), 110.9 (**12**), 100.4 (**7**), 84.0 (**4**), 30.1 (**2**), 24.8 (**5**), 18.9 (**1**).

HRMS-ESI/EI/APCI (m/z): mass ion not observed.

IR (v_{max}/ cm^{-1}, neat): 3675, 2987, 2901, 1614, 1325, 1262, 1067.

27 *tert*-butyl 5-(1-hydroxycyclobutyl)-1*H*-indole-1-carboxylate

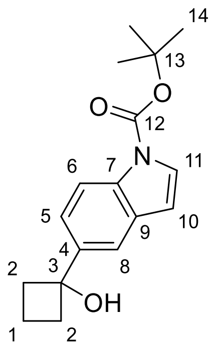

Compound **27** was synthesised following **GP2** using tert-butyl 5-(4,4,5,5-tetramethyl-1,3,2-dioxaborolan-2-yl)-1H-indole-1-carboxylate (104 mg, 0.30 mmol, 1.50 equiv.) as the starting boronic ester. The crude mixture was purified using flash column chromatography (5% to 10% Et_2O in pentane), to afford the title compound as a colourless oil (39 mg, 64%).

R*f* = 0.25 (20% Et_2O/pentane).

^{1}H NMR (400 MHz, $CDCl_3$): *δ* = 8.13 (1H, d, *J* = 8.7 Hz, **6**), 7.68 (1H, d, *J* = 1.9 Hz, **8**), 7.60 (1H, d, *J* = 3.8 Hz, **11**), 7.47 (1H, dd, *J* = 8.7, 1.9 Hz, **5**), 6.57 (1H, d, *J* = 3.8 Hz, **10**), 2.63 (2H, m, **2**), 2.42 (2H, m, **2**), 2.03 (1H, m, **1**), 1.70 (1H, m, **1**), 1.68 (9H, s, **14**), 1.26 (1H, s, **OH**).

Return to contents

^{13}C NMR (101 MHz, $CDCl_3$): δ = 149.8 (**12**), 140.8 (**7**), 134.4 (**9**), 130.5 (**4**), 126.4 (**11**), 121.8 (**5**), 117.2 (**8**), 115.2 (**6**), 107.5 (**10**), 83.7 (**13**), 77.3 (**3**), 37.0 (**2**), 28.2 (**14**), 13.1 (**1**).

HRMS-ESI (m/z): $[M+Na]^+$ calcd for $C_{17}H_{21}NNaO_3$, 310.1414; found, 310.1400.

IR (v_{max}/cm^{-1}, neat) 3350, 2982, 2935, 1731, 1368, 1138, 1022, 725.

28 (*E*)-1-(4-((tert-butyldimethylsilyl)oxy)but-2-en-2-yl)cyclobutan-1-ol

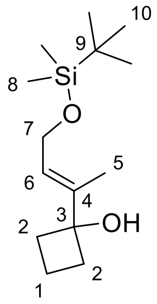

Compound **28** was synthesised following **GP2** using (Z)-tert-butyldimethyl((3-(4,4,5,5-tetramethyl-1,3,2-dioxaborolan-2-yl)but-2-en-1-yl)oxy)silane (116.9 mg, 0.37 mmol, 1.5 eq) as the starting boronic ester. NMR yield of crude product was 88% prior to oxidation. Purification via silica column chromatography, eluting with 20-30% Et_2O in pentane, afforded the title compound as a clear oil with minor impurities and so clean fractions were taken for analysis. NMR yield 49% (using CH_2Br_2 as internal standard).

R_f = 0.30 (30% Et_2O/ pentane).

^{1}H NMR (400 MHz, $CDCl_3$): δ 5.49 (1H, tq, *J* = 5.9, 1.1 Hz, **6**), 4.18 (2H, d, *J* = 5.9 Hz, **7**), 2.28-2.19 (2H, m, **2**), 2.00-1.90 (2H, m, **2**), 1.88-1.77 (1H, m, **1**), 1.57 (3H, d, *J* = 1.1 Hz **5**), 1.51-1.42 (1H, m, **1**), 0.83 (9H, s, **10**), 0.00 (6H, s, **8**).

^{13}C NMR (101 MHz, $CDCl_3$): δ 138.4 (**4**), 124.1 (**6**), 78.8 (**3**), 60.5 (**7**), 33.9 (**2**), 26.0 (**10**), 18.4 (**9**), 13.0 (**1**), 11.6 (**5**), −5.08 (**8**).

HRMS-ESI (m/z): $[M+H-H_2O]^+$ calcd for $C_{14}H_{27}OSi$, 239.1826; found, 239.1829.

IR (v_{max}/cm^{-1}, neat) 3358, 2953, 2930, 2357, 1472, 1253, 1063, 834;

29 (*E*)-4,4,5,5-tetramethyl-2-(1-styrylcyclobutyl)-1,3,2-dioxaborolane

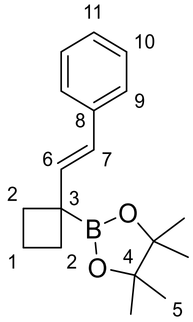

Compound **29** was synthesised following GP1 using (E)-4,4,5,5-tetramethyl-2-styryl-1,3,2-dioxaborolane (69 mg, 0.30 mmol, 1.50 equiv.) as the starting boronic ester. The crude mixture was purified using flash column chromatography (5–0% CH_2Cl_2/pentane), to afford the title compound as a colourless oil (39 mg, 69%).

R*f* = 0.5 (30% CH_2Cl_2/pentane);

^{1}H NMR (400 MHz, $CDCl_3$): *δ* = 7.35 – 7.29 (m, 2H, **10**), 7.25 – 7.21 (m, 2H, **9**), 7.13 (tt, *J* = 6.6, 1.3 Hz, 1H, **11**), 6.42 (1H, d, *J* = 16.0 Hz, **6**), 6.24 (1H, d, *J* = 16.0 Hz, **7**), 2.28 (2H, m, **2**), 2.06 (2H, ddd (app. q), *J* = 9.0 Hz, **2**), 1.96 (1H, m, **1**), 1.85 (1H, m, **1**), 1.23 (12H, s, **5**).

^{13}C NMR (101 MHz, $CDCl_3$): *δ* = 138.4 (**8**), 137.0 (**6**), 128.5 (**9**), 126.6 (**11**), 126.12(**7**), 126.1 (**10**), 83.5 (**4**), 30.81 (**2**), 24.8 (**5**), 18.7 (**1**).

HRMS-ESI (m/z): $[M+Na]^+$ calcd for $C_{18}H_{25}BNaO_2$, 307.1843; found, 307.1857.

IR (ν_{max}/cm^{-1}, neat): 2977, 1357, 1313, 1144, 1112, 965, 745, 694.

30 (*E*)-4,4,5,5-tetramethyl-2-(1-(3-methylbut-1-en-1-yl)cyclobutyl)-1,3,2-dioxaborolane

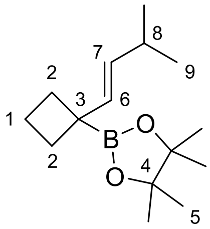

Compound **30** was synthesised following GP1 using (*E*)-4,4,5,5-tetramethyl-2-(3-methylbut-1-en-1-yl)-1,3,2-dioxaborolane (73.5 mg, 0.375 mmol, 1.50 equiv.) as the starting boronic ester. The crude mixture was purified using flash column

chromatography (25% CH_2Cl_2/pentane), to afford the title compound as a colourless oil (40 mg, 64%).

R_f = 0.30 (25% CH_2Cl_2/pentane);

^{1}H NMR (400 MHz, $CDCl_3$): δ = 5.56 (dd, *J* = 15.6, 1.3 Hz, 1H, **6**), 5.26 (dd, *J* = 15.6, 6.7 Hz, 1H, **7**), 2.25 (octd, *J* = 6.7, 1.3 Hz, 1H, **8**), 2.22 – 2.17 (m, 2H, **2**), 1.97 – 1.86 (m, 2H, **2**), 1.87 – 1.77 (m, 2H, **1**), 1.25 (s, 12H, **5**), 0.96 (d, *J* = 6.7 Hz, 6H, **9**).

^{13}C NMR (101 MHz, $CDCl_3$): δ = 134.3 (**7**), 133.3 (**6**), 83.3 (**4**), 31.2 (**8**), 31.0 (**2**), 24.7 (**5**), 23.0 (**9**), 18.7 (**1**).

HRMS-ESI (m/z): no mass ion found.

IR (ν_{max}/cm^{-1}, neat): 2990, 1354, 1314, 1155, 1088, 850.

6.3 Boronic ester functionalisation using boronic ester 19

6.3.1 Zweifel Olefination

31 *tert*-butyl 4-(1-(prop-1-en-2-yl)cyclobutyl)piperidine-1-carboxylate

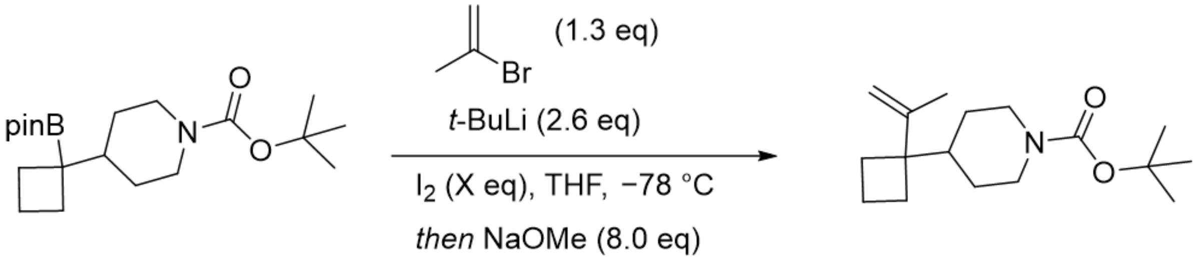

Freshly distilled 2-bromopropene (21 mg, 0.18 mmol, 1.3 equiv.) was dissolved in THF (2 mL). The solution was cooled down to −78 °C and a freshly titrated pentane *tert*-butyllithium solution (1.9 M, 0.29 mL, 0.54 mmol, 2.6 equiv.) was added dropwise. After stirring at −78 °C for 1 h, a solution of boronic ester **19** (50 mg, 0.14 mmol in 1.4 mL of THF, 1.0 equiv.) was added dropwise at the same temperature. The reaction was stirred 5 minutes at −78 °C, then warmed up to room temperature and stirred for 1 h. The reaction was cooled down to −78 °C and a solution of iodine (106 mg in 0.7 mL THF) was added dropwise. The solution was stirred 15 minutes at −78 °C, followed by further 15 minutes at room temperature. The solution was cooled down again to −78 °C and NaOMe (59 mg, 1.12 mmol, 8.0 equiv.) was added under nitrogen flow, followed by anhydrous methanol (1 mL). The reaction was stirred 10 minutes at −78 °C followed by 1 h at room temperature. The reaction was diluted with pentane (15 mL), washed with a sat. $Na_2S_2O_3$ solution (5 mL) and brine (5 mL). The organic phase was dried over magnesium sulfate and solvents evaporated. The residue was passed through an SiO_2 pipette column (6 cm) using pure pentane as eluent. Solvents were evaporated to afford the title compound **31** as a colourless oil (30 mg, 80%).

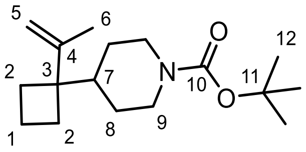

Rf = 0.42 (5% Et_2O/pentane, *p*-anisaldehyde);

^{1}H NMR (400 MHz, $CDCl_3$): δ = 4.87 (quin, *J* = 1.5 Hz, 1H, **5**), 4.63 (br s, 1H , **5**), 4.17 (br s, 2H, **9**), 2.60 (t, *J* = 12.2 Hz, 2H, **9**), 2.10 – 1.88 (m, 4H, **2**), 1.76 – 1.65 (quintet, *J* = 8.3, 7.8 Hz, **1**), 1.65 – 1.57 (m, 5H, **6 & 8**), 1.48 (tt, *J* = 12.0, 3.2 Hz, 1H, **7**), 1.45 (s, 9H, **12**), 1.16 (qd, *J* = 12.6, 4.5 Hz, 2H, **8**) ppm;

^{13}C NMR (101 MHz, $CDCl_3$): δ = 155.0 (**10**), 150.3 (**4**), 110.9 (**7**), 79.3 (**11**), 50.6 (**4**), 44.7 (br, **9**), 41.8 (**7**), 28.6 (**12**), 28.5 (**2**), 27.0 (**8**), 19.03 (**6**), 15.2 (**1**) ppm;

HRMS-ESI$^+$ (*m/z*): [M + Na]+ calculated for $C_{17}H_{29}NNaO_2$, 302.2091; found, 302.2089;

IR (ν_{max}/ cm^{-1}, neat): 3675, 2973, 2901, 1692, 1451, 1415, 1276, 1066, 890, 764;

6.3.2 Matteson Homologation

32 *tert*-butyl 4-(1-((4,4,5,5-tetramethyl-1,3,2-dioxaborolan-2-yl)methyl)cyclobutyl)piperidine-1-carboxylate

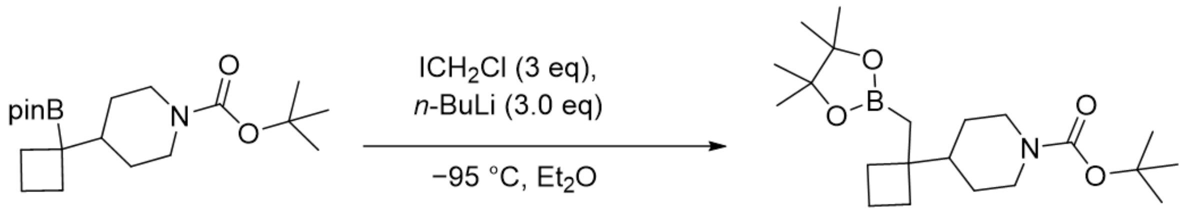

According to a literature procedure,[7] boronic ester **19** (50 mg, 0.14 mmol, 1.0 equiv.) and iodochloromethane (30 µL, 0.41 mmol, 3.0 equiv.) were dissolved in diethyl ether (1 mL) and cooled to −95 °C (methanol/liquid nitrogen bath). *n*-Butyllithium (0.39 mL, 0.62 mmol, 1.6 M solution in *n*-hexane, 2.95 equiv.) was added dropwise and the solution was stirred 10 minutes at −95 °C, followed by additional 1 h at room temperature. The reaction mixture was then filtered through a silica plug, eluting with Et_2O. The solvents were evaporated under reduced pressure to afford crude product, which was purified by silica column chromatography, eluting with 5-15% EtOAc/ pentane, afforded the title compound **32** (33 mg, 63%) as a colourless oil.

R_f = 0. (10% EtOAc/pentane, *p*-anisaldehyde);

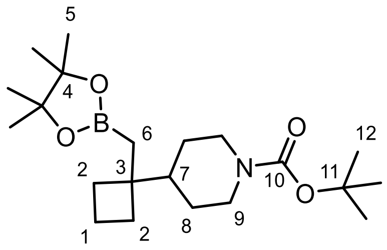

^{1}H NMR (400 MHz, $CDCl_3$): δ = 4.15 (br d, J = 13.3 Hz, 2H, **9**), 2.58 (td, J = 12.8, 2.5 Hz, 2H, **9**), 1.92 – 1.78 (m, 5H, **1 & 2**), 1.78 – 1.66 (m, 2H, **1**), 1.57 (br d, J = 12.8 Hz, 2H, **8**), 1.44 (s, 9H, **12**), 1.40 (tt, J = 12.0, 3.2 Hz, 1H, **7**), 1.24 (s, 12H, **5**), 1.10 (qd, J = 12.4, 3.8 Hz, 1H, **8**), 0.89 (s, 2H, **6**);

^{13}C NMR (101 MHz, $CDCl_3$): δ = 155.0 (**10**), 83.0 (**4**), 79.2 (**11**), 46.5 (**7**), 44.7 (**9**), 42.6 (**3**), 31.2 (**2**), 28.6 (**12**), 27.0 (**7**), 26.4 (**8**), 25.1 (**5**), 19.7 (br, **6**), 14.7 (**1**);

HRMS-ESI$^+$ (*m/z*): [M + Na]+ calculated for $C_{21}H_{39}BNO_4$, 380.2976; found, 380.2978;

IR (ν_{max}/ cm^{-1}, neat): 3675, 2974, 1693, 1410, 1364, 1275, 1144, 1057, 891, 764;

6.3.3 Amination

33 tert-butyl 4-(1-((tert-butoxycarbonyl)amino)cyclobutyl)piperidine-1-carboxylate

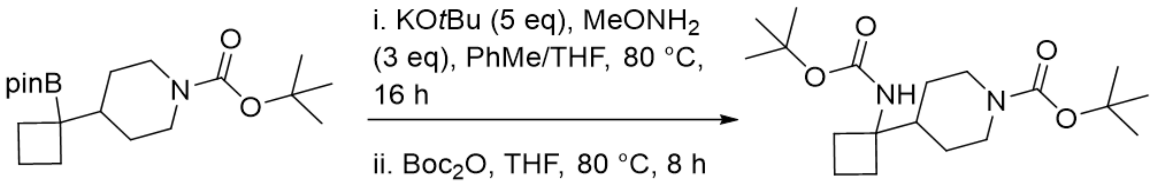

According to a literature procedure, boronic ester **19** (50 mg, 0.14 mmol, 1.00 equiv.), potassium *tert*-butoxide (77 mg, 0.70 mmol, 5.00 equiv.) and *O*-methylhydroxylamine (0.19 mL, 19 mg, 0.42 mmol, 3.00 equiv.) in anhydrous toluene (0.61 mL) and anhydrous THF (0.07 mL) were heated to 80 °C for 16 h. Following this, the reaction was cooled to ambient temperature and di-*tert*-butyl dicarbonate (0.08 mL, 0.35 mmol, 2.50 equiv.) in THF (0.50 mL) was added concurrently with a solution of saturated aqueous $NaHCO_3$ (0.50 mL). The reaction was then re-heated to 80 °C and stirred for a further 8 h before cooling to ambient temperature, adding water (10 mL) and extracting with ethyl acetate (3 × 10 mL). The combined organic fractions were then dried ($MgSO_4$), filtered and concentrated *in vacuo* to give a crude residue, which was purified by silica column chromatography (0-20% EtOAc/ pentane) to afford the desired amine **33** (29 mg, 60%) as a colourless oil.

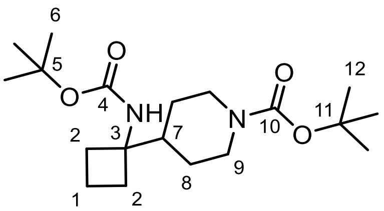

R_f = 0.59 (20% EtOAc/pentane, ninhydrin);

^{1}H NMR (400 MHz, $CDCl_3$): δ = 4.56 (br s, 1H, **NH**), 4.19 (d, J = 13.1 Hz, 2H, **9**), 2.64 (td, J = 13.0, 2.5 Hz, 2H, **9**), 2.13 (dd, J = 8.8, 6.4 Hz, 4H, **2**), 2.03 – 1.79 (m, 1H, **1**),

1.91 – 1.80 (m, 1H, **7**), 1.74 – 1.58 (m, 3H, **1 & 8**), 1.45 (s, 9H, **6/12**), 1.43 (s, 9H, **6/12**), 1.17 (qd, J = 12.7, 4.4 Hz, 2H, **8**).

^{13}C NMR (101 MHz, $CDCl_3$): δ = 159.9 (**4/10**), 155.0 (**4/10**), 79.5 (**5 & 11**), 59.3 (**3**), 44.3 (**9**), 42.7 (**7**), 29.8 (**2**), 28.6 (**6/12**), 28.5 (**6/12**), 26.2 (**8**), 14.9 (**1**) ppm;

HRMS-ESI$^+$ (*m/z*): [M + Na]+ calculated for $C_{19}H_{34}N_2O_4Na$, 377.2411; found, 377.2411;

IR (ν_{max}/ cm^{-1}, neat): 3674, 3346, 2973, 1694, 1408, 1394, 1275, 1066, 869, 750;

6.3.4 Alkynalation

34 *tert*-butyl 4-(1-ethynylcyclobutyl)piperidine-1-carboxylate

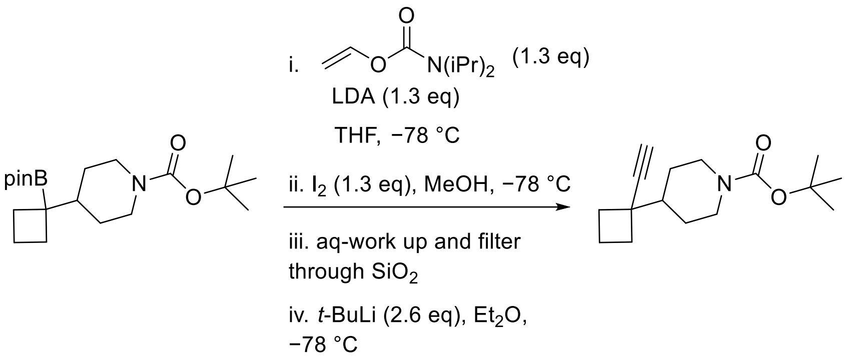

According to a literature procedure,[8] to a stirred solution of vinyl diisopropylcarbamate (49 mg, 0.18 mmol, 1.3 equiv.) and boronic ester **19** (50 mg, 0.14 mmol, 1.0 equiv.) in THF (0.5 mL) under N_2 at –78 °C was added freshly prepared LDA (0.86 M in THF, 0.21 mL, 0.18 mmol, 1.3 equiv.) dropwise at a rate of approximately 10 µL every 10 s. The resulting solution was stirred for 1 h at –78 °C before the addition of a solution of I_2 (45 mg, 0.18 mmol, 1.3 equiv.) in MeOH (0.5 mL) dropwise over 5 min. The reaction was stirred for 5 min at –78 °C before warming to r.t. and stirred for 1 h. The reaction was quenched by the addition of 20% $Na_2S_2O_3$ (5 mL) and the aqueous phase extracted with Et_2O (2 × 7.5 mL). The combined organic phases were washed with water (7.5 mL), dried over $MgSO_4$, filtered through a short pad of silica gel (1 cm) and concentrated *in vacuo*. The crude product was then re-dissolved in Et_2O (1.5 ml) cooled to –78 °C and *t*BuLi (0.21 mL, 1.7 M, 0.36 mmol, 2.55 eq) was added. The reaction was then transferred to a 0 °C bath and stirred for 0.5 hbefore the addition of saturated $NH_4Cl_{(aq.)}$ (5 mL). The aqueous phase was extracted with Et_2O (2 × 7.5 mL) and the combined organic phases dried over $MgSO_4$, filtered, and concentrated *in vacuo*. The crude product was purified by flash column chromatography on silica gel

(0 – 10% Et_2O/ pentane) to give the desired product **34** as a colourless oil (30.6 mg, 83%).

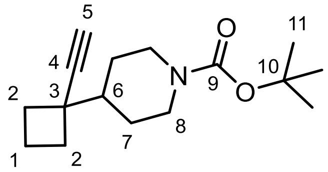

R*f* = 0.53 (10% Et_2O/pentane, $KMnO_4$);

^{1}H NMR (400 MHz, $CDCl_3$): δ = 4.16 (br d, J = 13.3 Hz, 2H, **8**), 2.61 (td, J = 13.4, 2.6 Hz, 2H, **8**), 2.23 – 2.07 (m, 4H, **1, 2 & 5**), 2.06 – 1.96 (m, 2H, **2**), 1.88 – 1.73 (m, 1H, **1**), 1.58 (br d, *J* = 11.7 Hz, 2H, **7**), 1.47 (tt, *J* = 11.6, 3.3 Hz, 1H, **6**), 1.44 (s, 9H, **11**), 1.31 (qd, *J* = 12.5, 4.5 Hz, 2H, **7**).

^{13}C NMR (101 MHz, $CDCl_3$): δ 155.0 (**9**), 89.2 (**4**), 79.4 (**10**), 70.5 (**5**), 45.3 (**6**), 44.2 (br, **8**), 40.6 (**3**), 33.1 (**2**), 28.6 (**11**), 26.5 (**7**), 16.4 (**1**).

HRMS-ESI$^+$ (*m/z*): $[M + Na]^+$ calculated for $C_{16}H_{25}NNaO_2$, 286.1778; found, 286.1786.

IR (ν_{max}/ cm^{-1}, neat): 3675, 3305, 2973, 2106, 1688, 1412, 1276, 1066, 868, 764.

Return to contents

7 Spectra

1 Cyclopropyl 2,4,6-triisopropylbenzoate

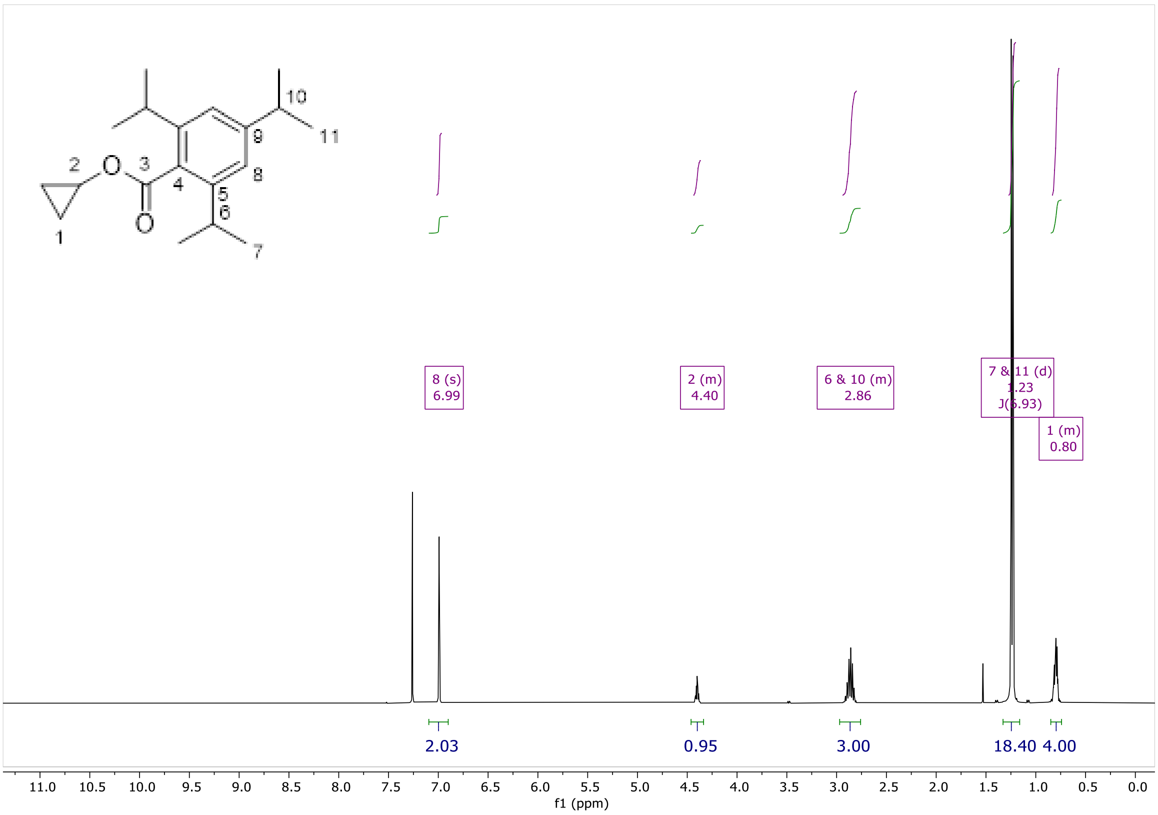

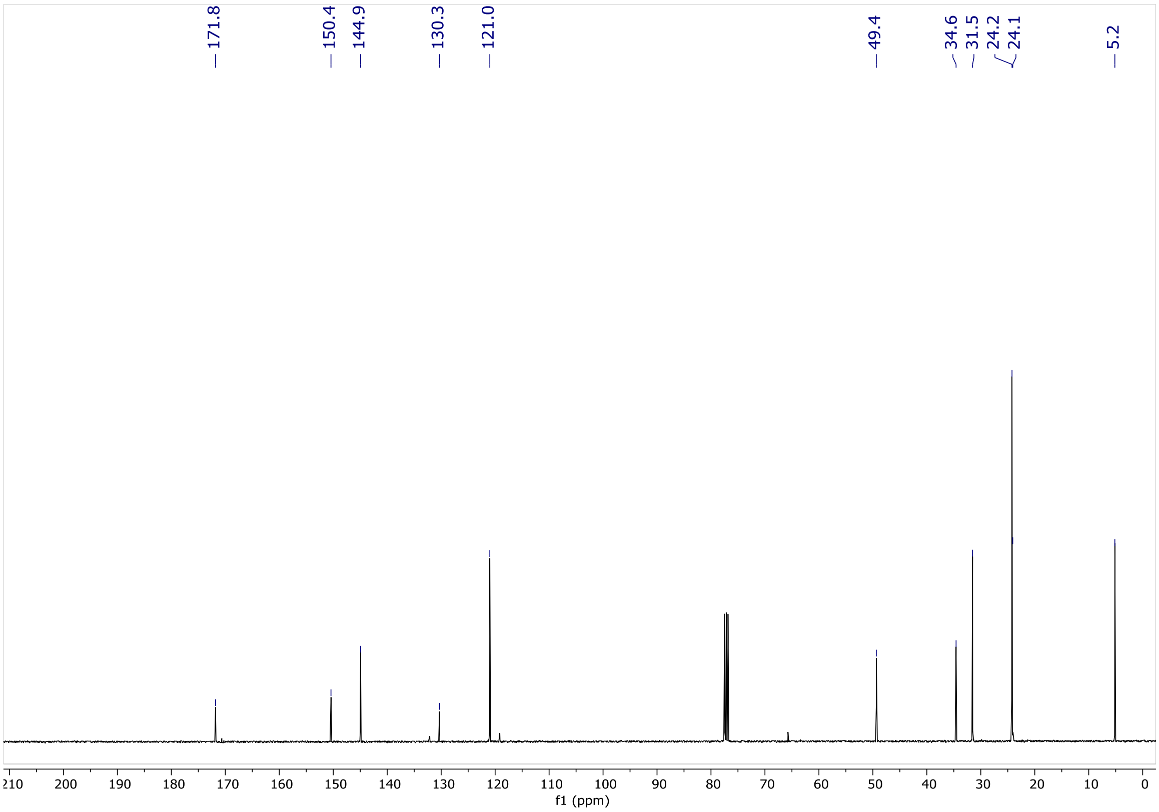

Return to contents

2 Cyclobutyl 2,4,6-triisopropylbenzoate

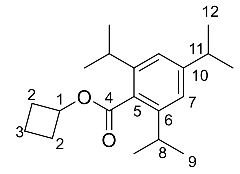

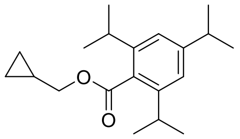

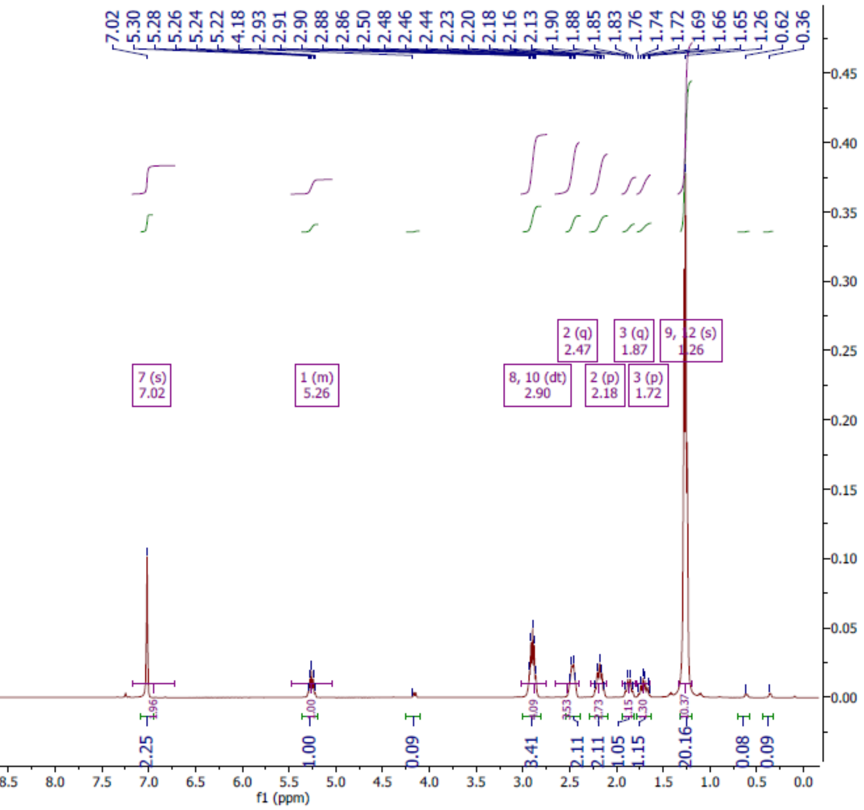

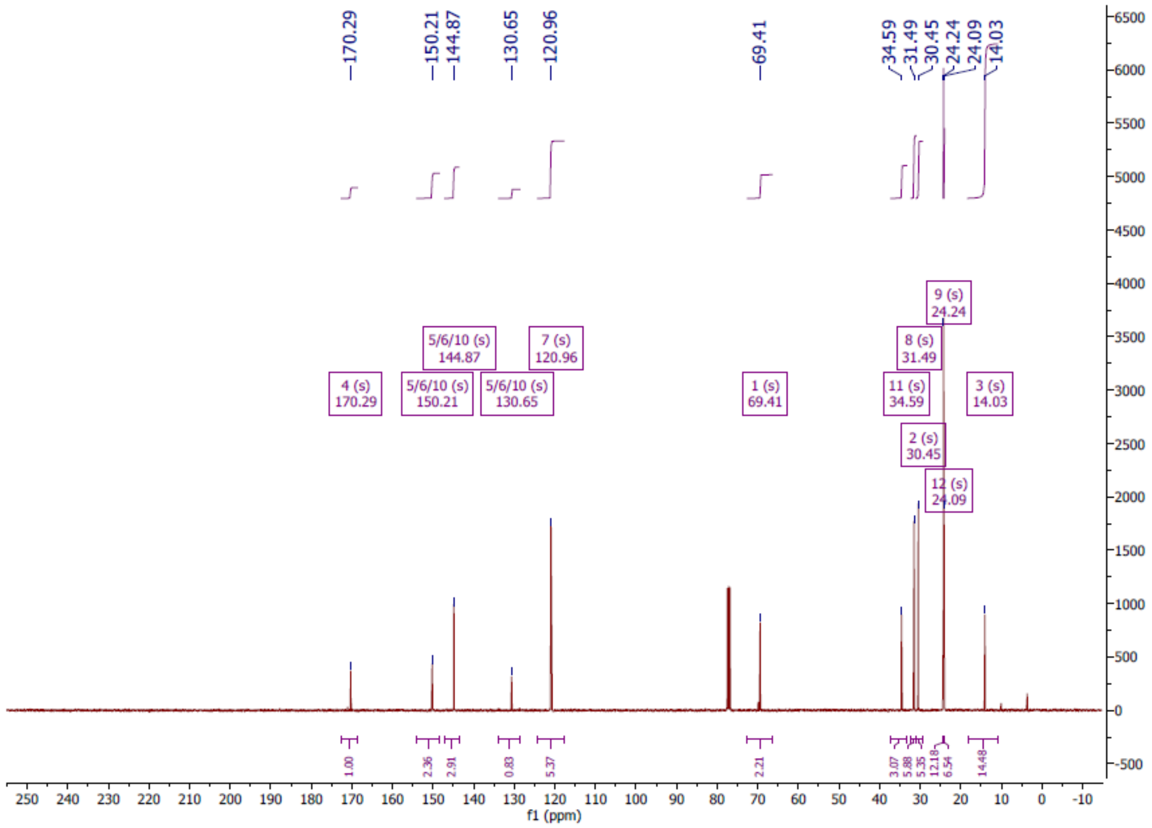

[Return to contents](#)

3 Cyclopentyl 2,4,6-triisopropylbenzoate

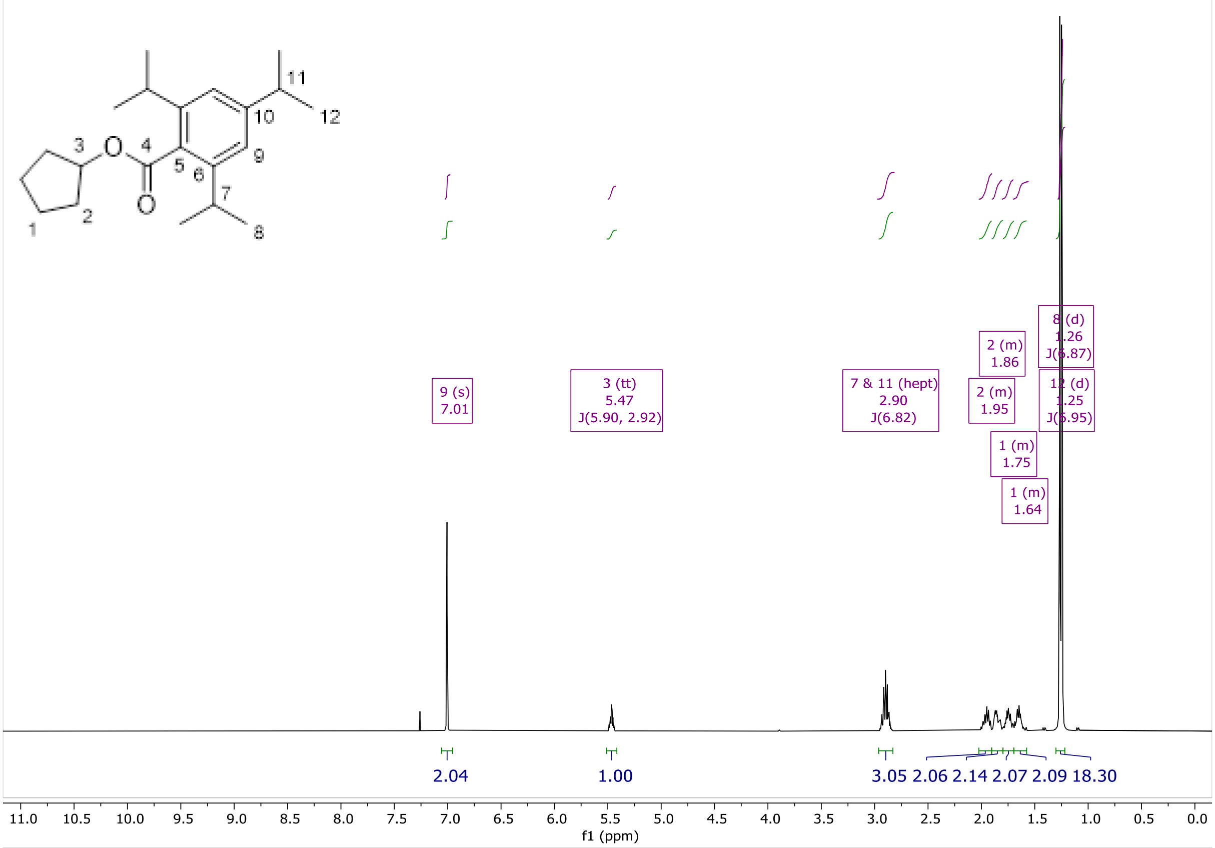

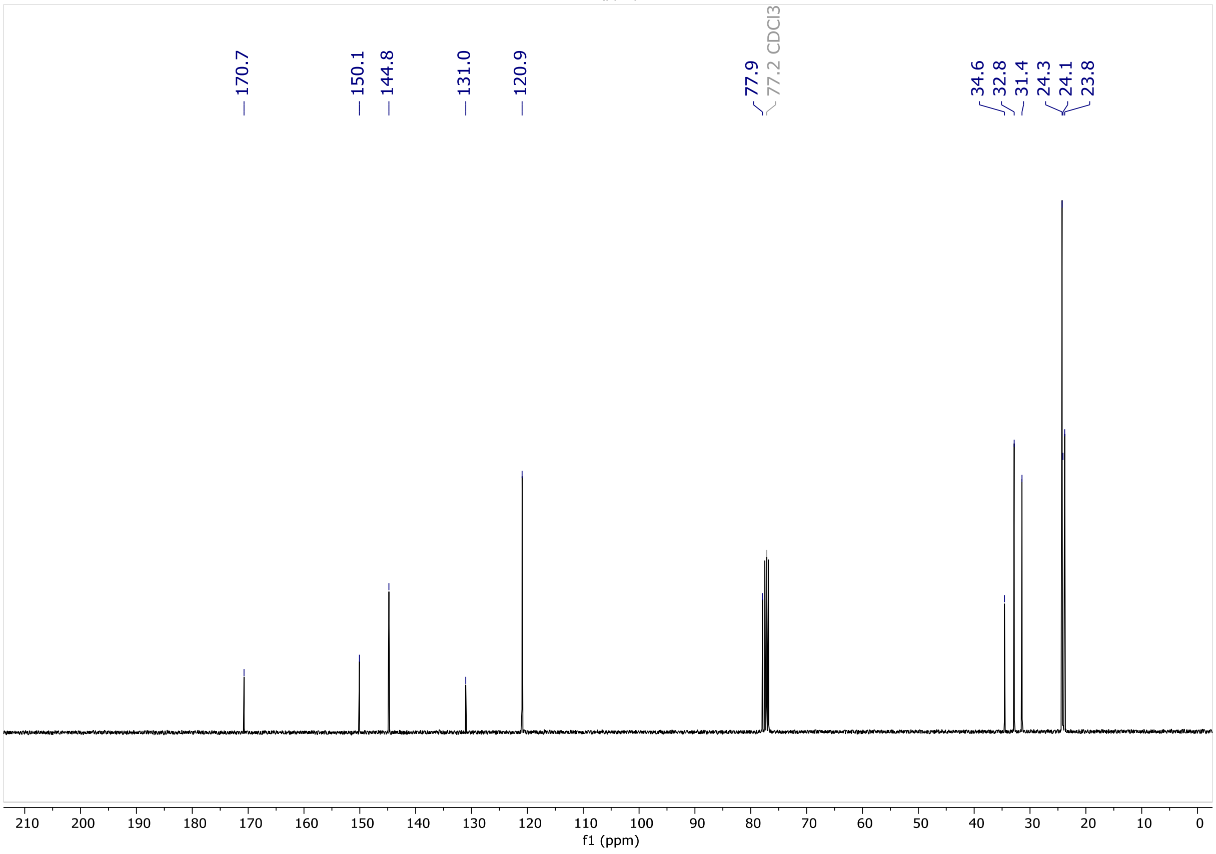

Return to contents

4 Cyclohexyl 2,4,6-triisopropylbenzoate

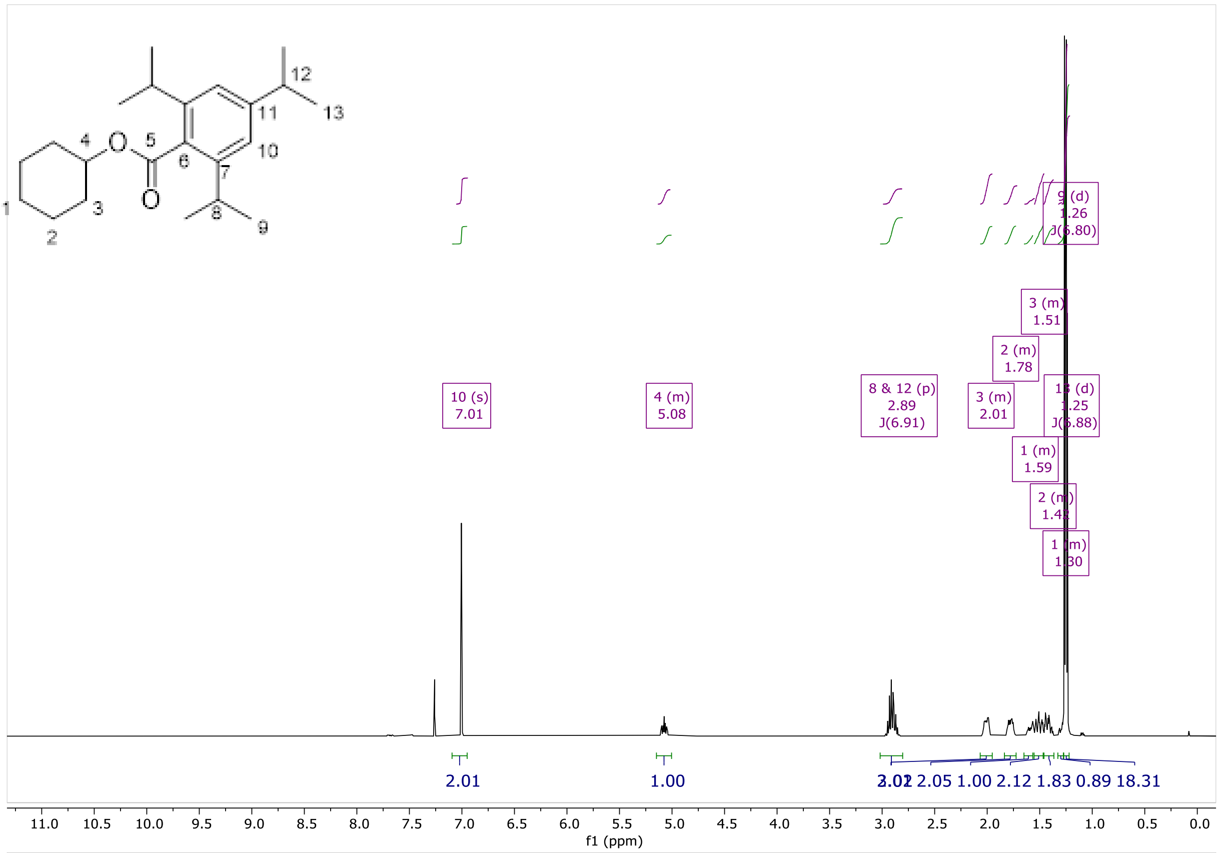

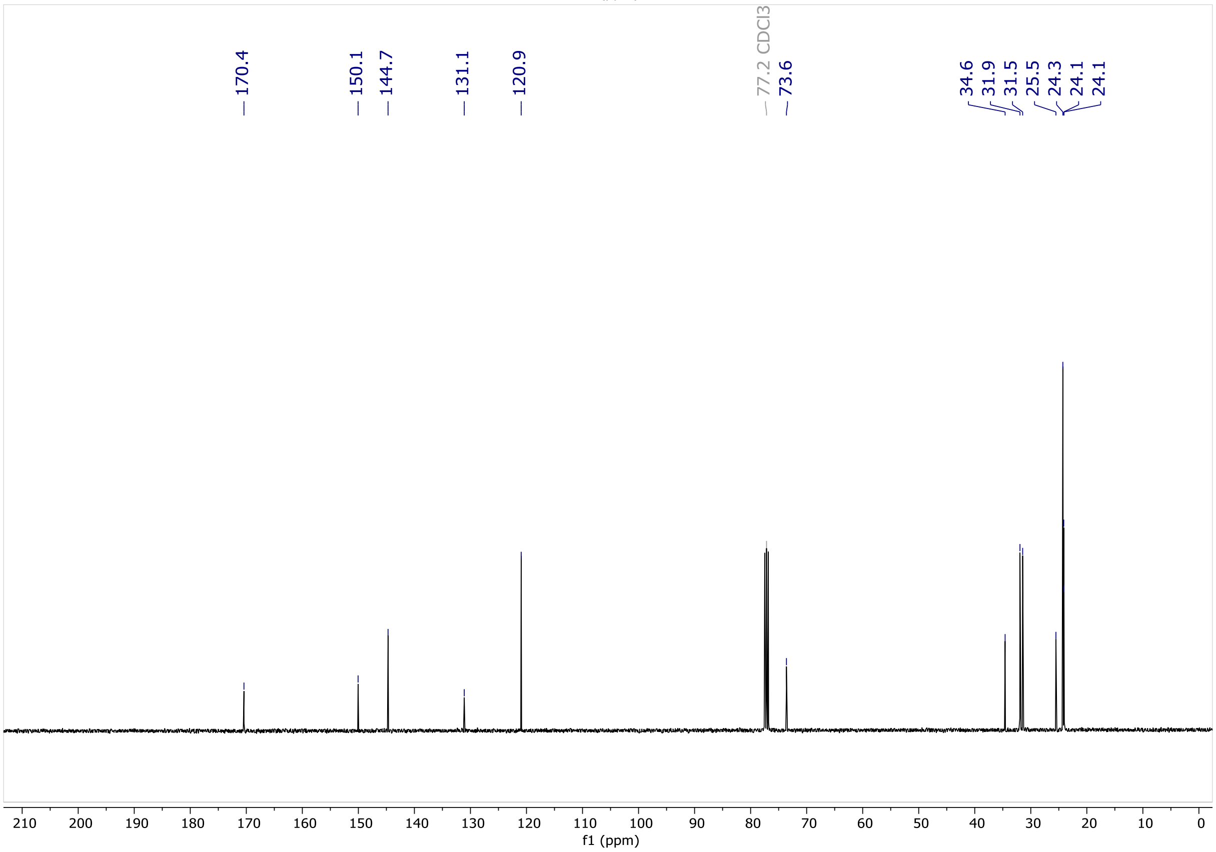

Return to contents

2-Cb Cyclobutyl carbamate

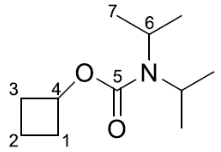

74

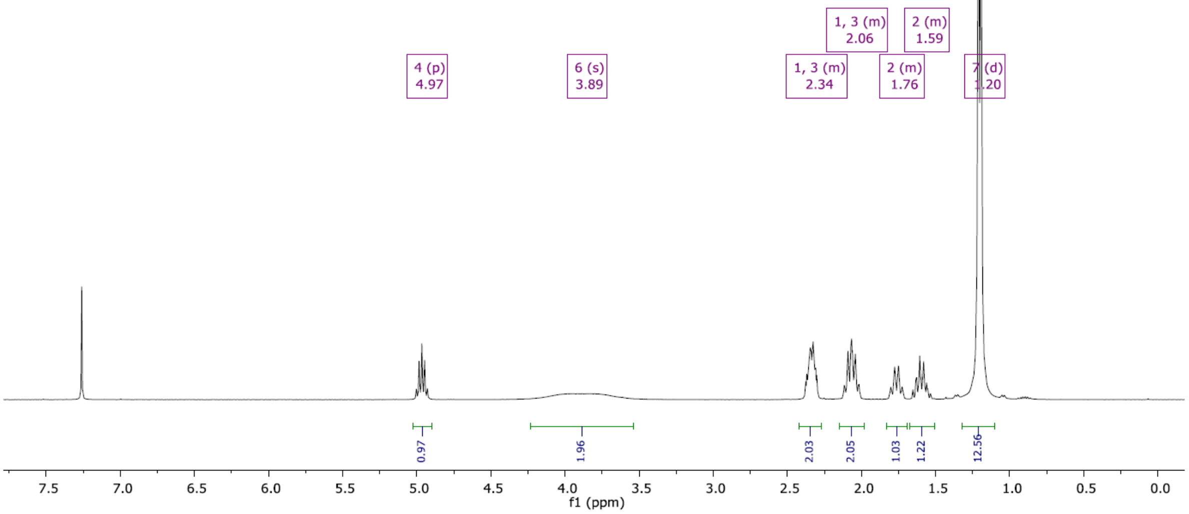

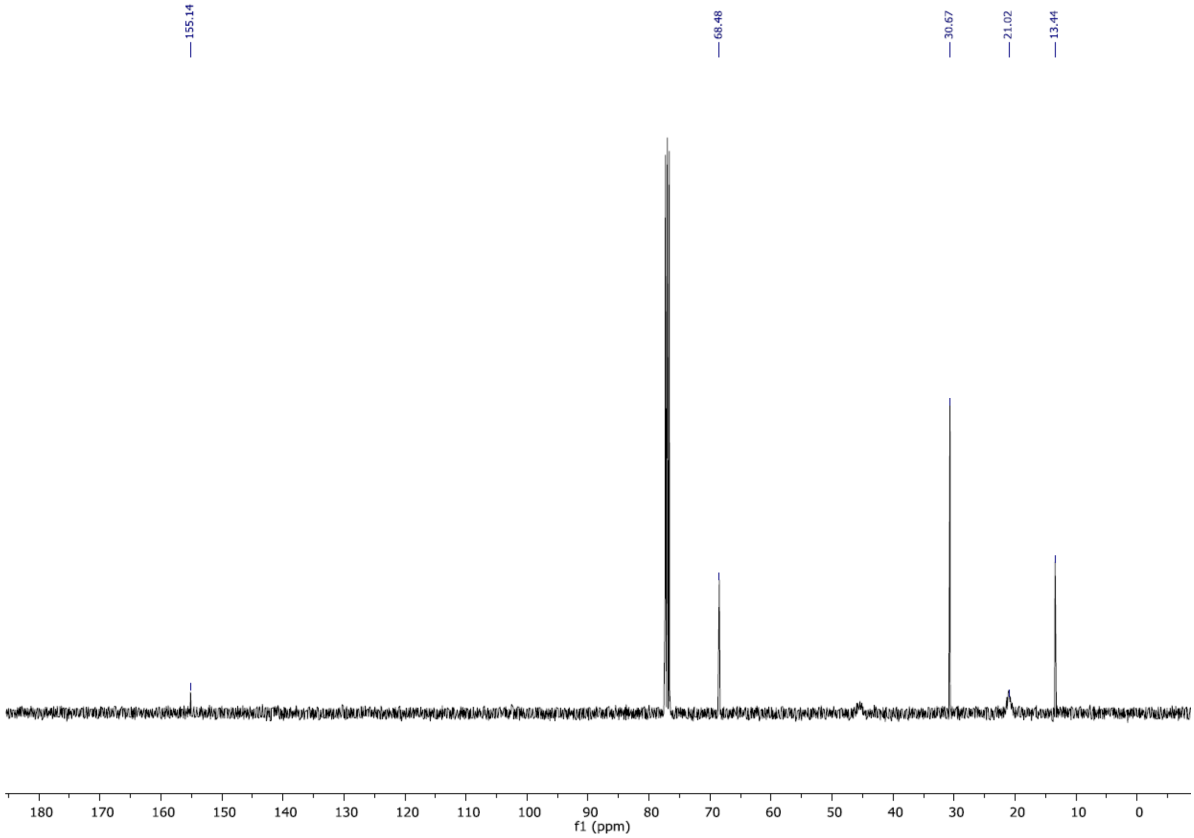

Return to contents

3-Cb Cyclopentyl Carbamate

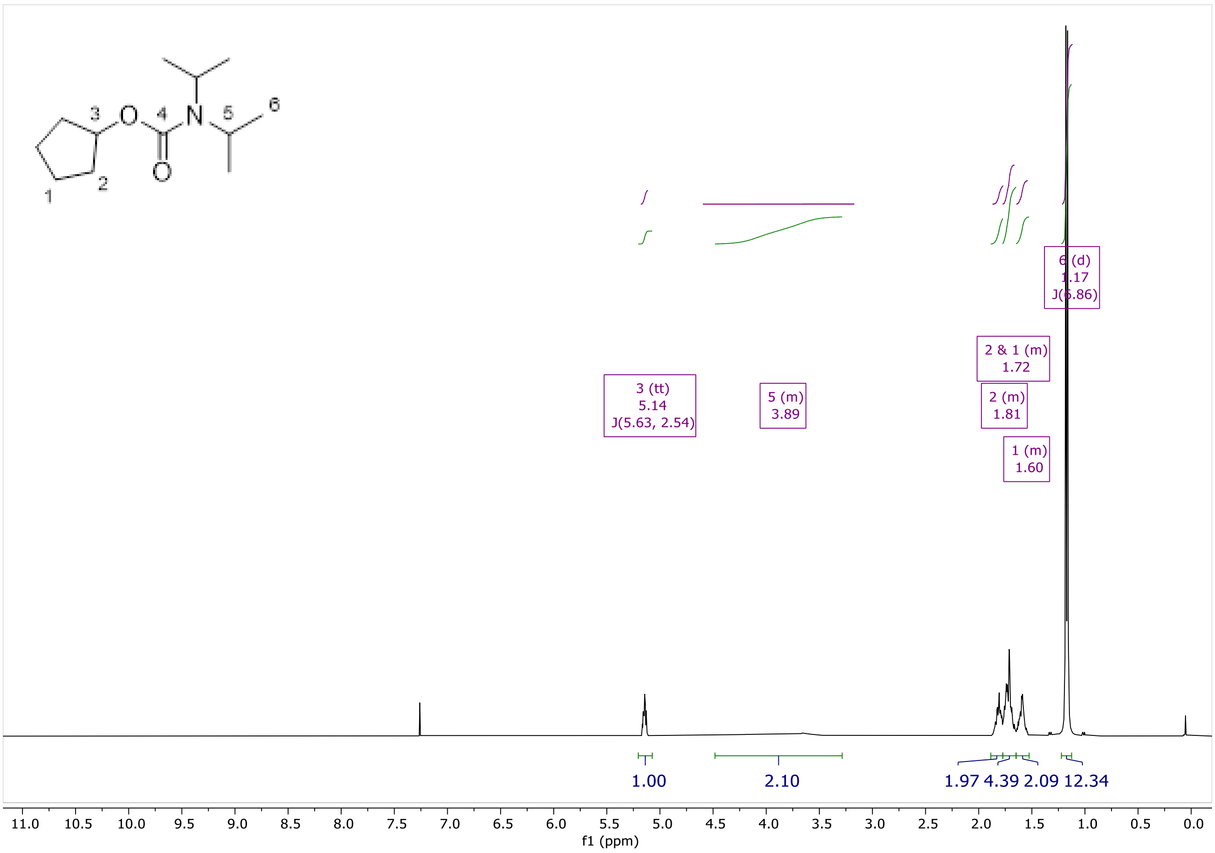

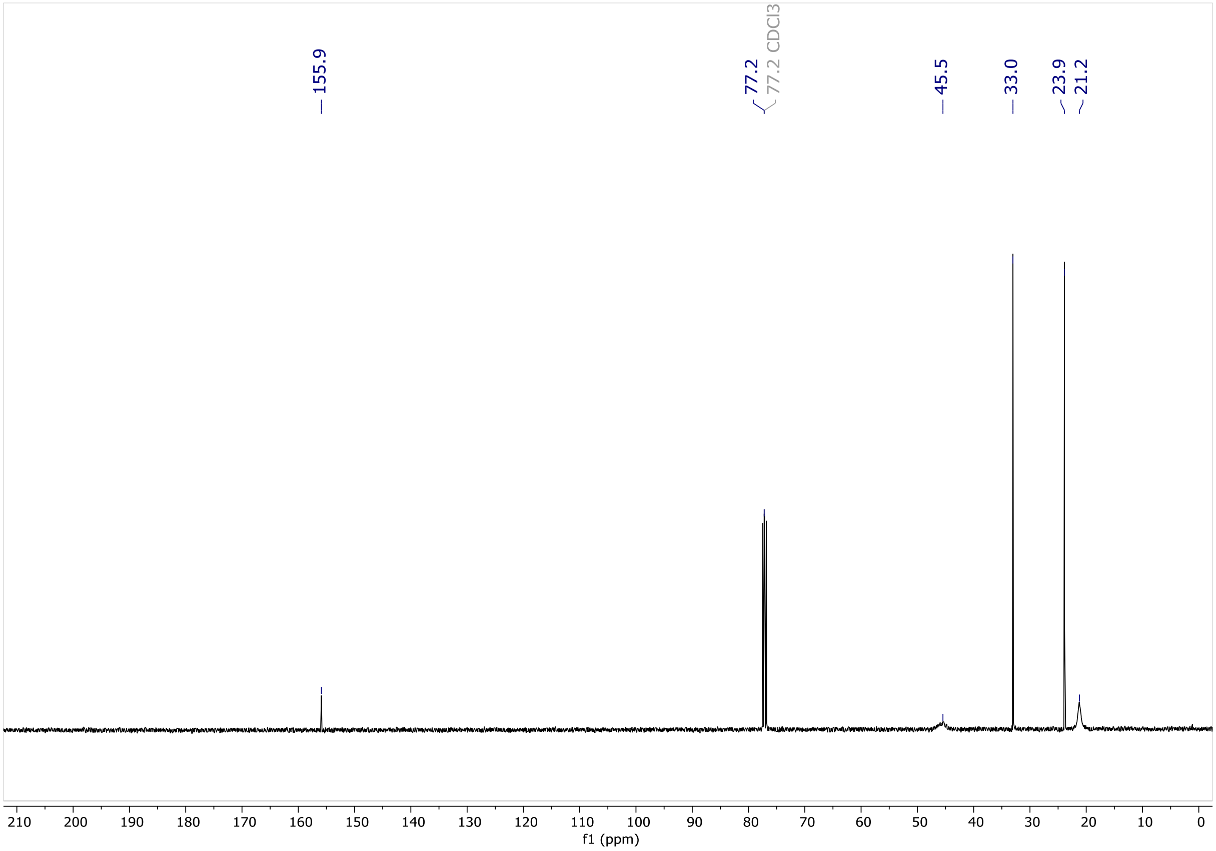

Return to contents

7 4,4,5,5-tetramethyl-2-(1-phenethylcyclobutyl)-1,3,2-dioxaborolane

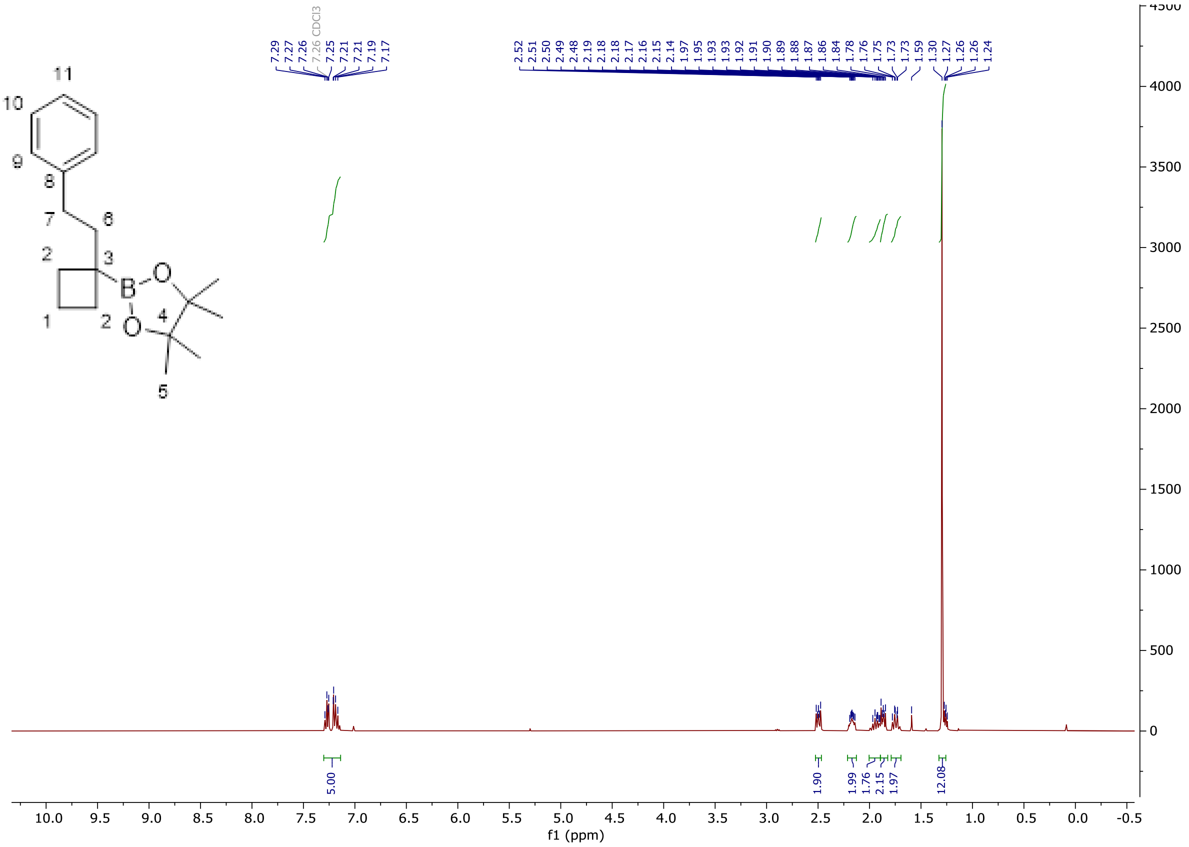

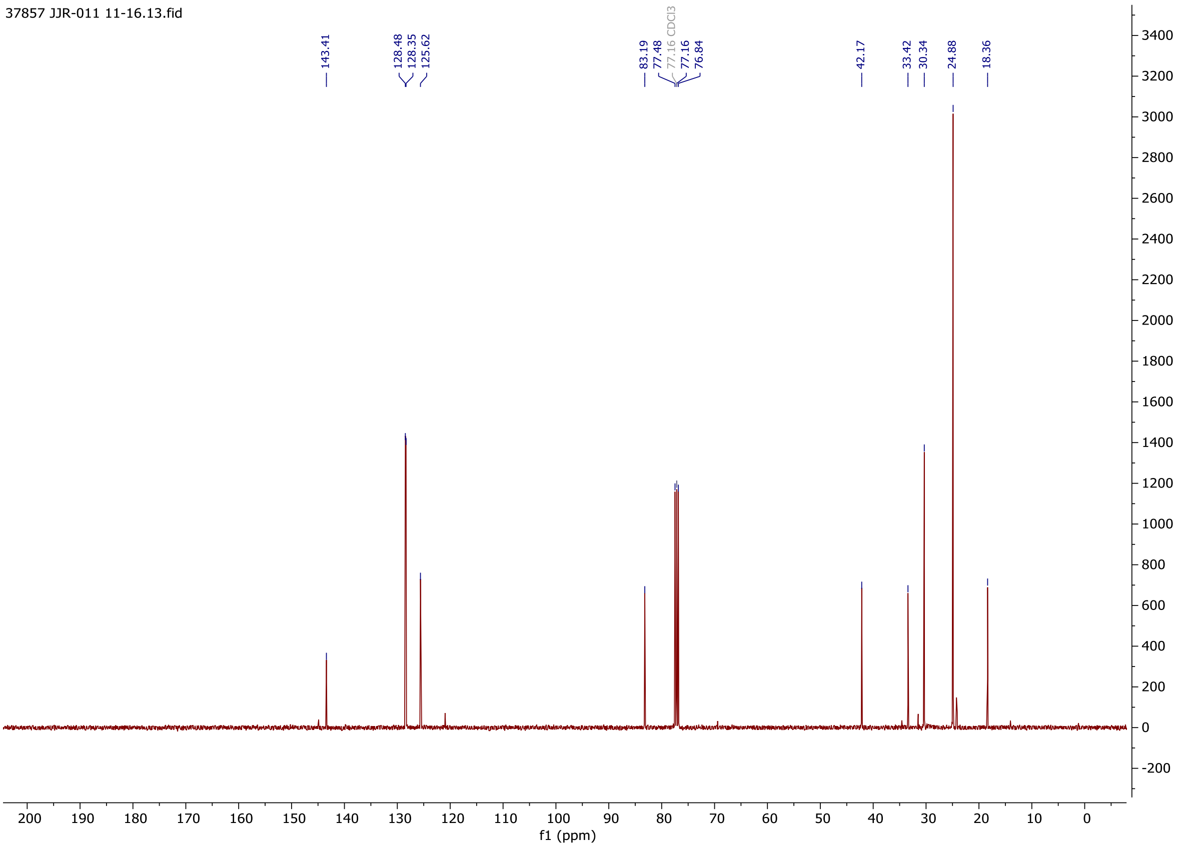

Return to contents

9 2-(1-(4-methoxybenzyl)cyclobutyl)-4,4,5,5-tetramethyl-1,3,2-dioxaborolane

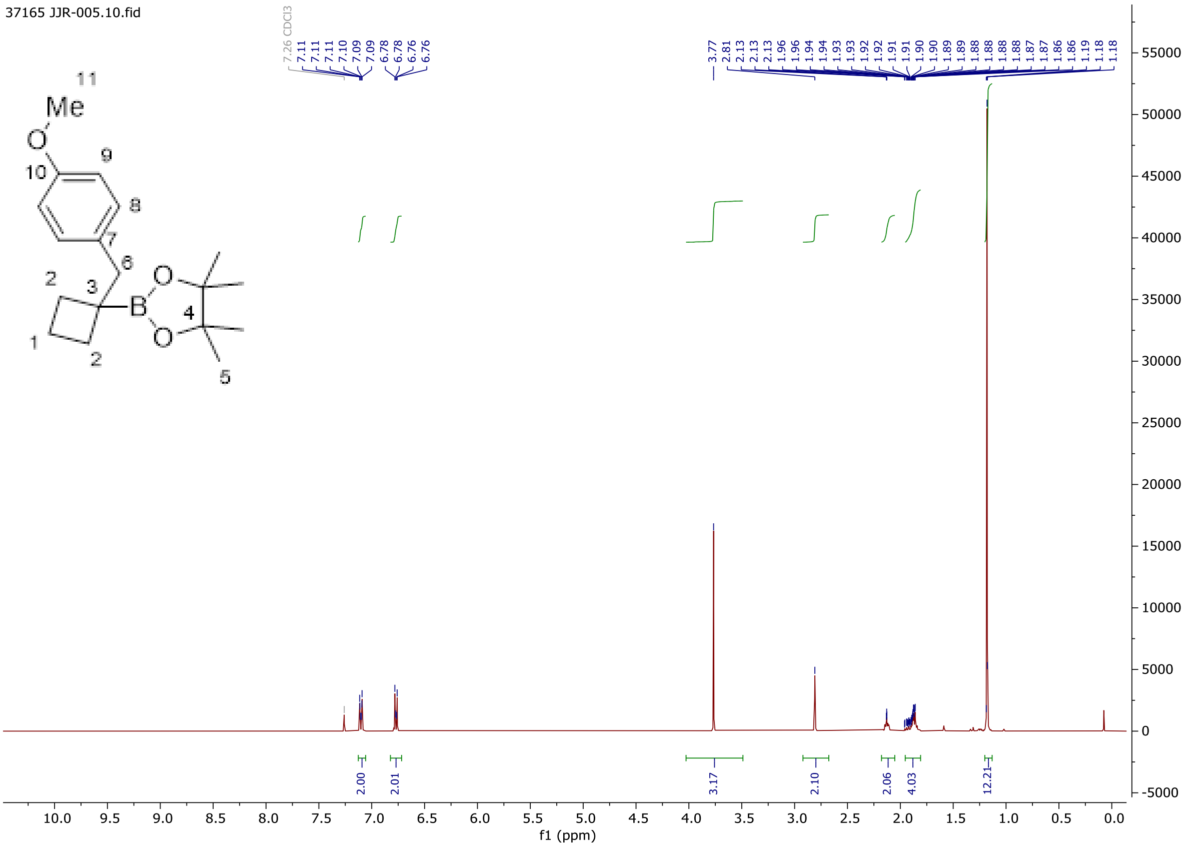

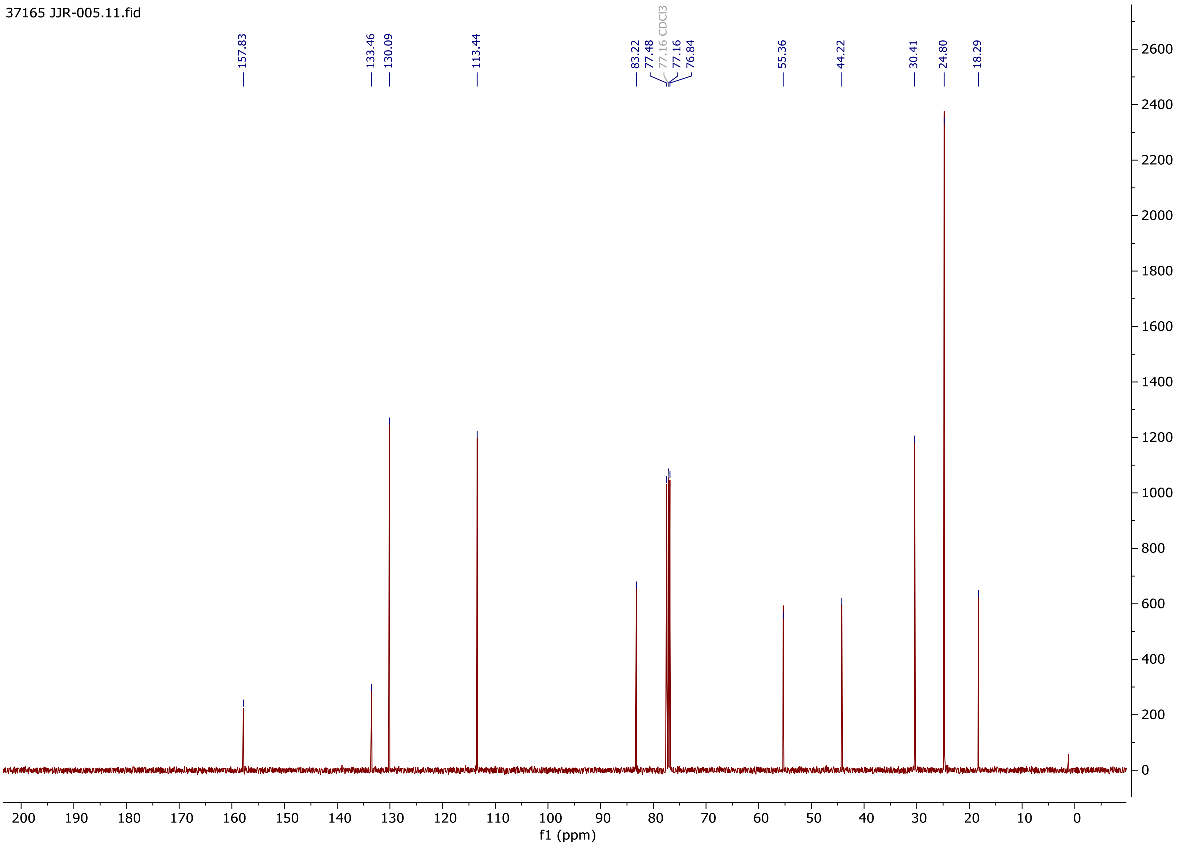

Return to contents

10 3-(1-(4,4,5,5-tetramethyl-1,3,2-dioxaborolan-2-yl)cyclobutyl)propanenitrile

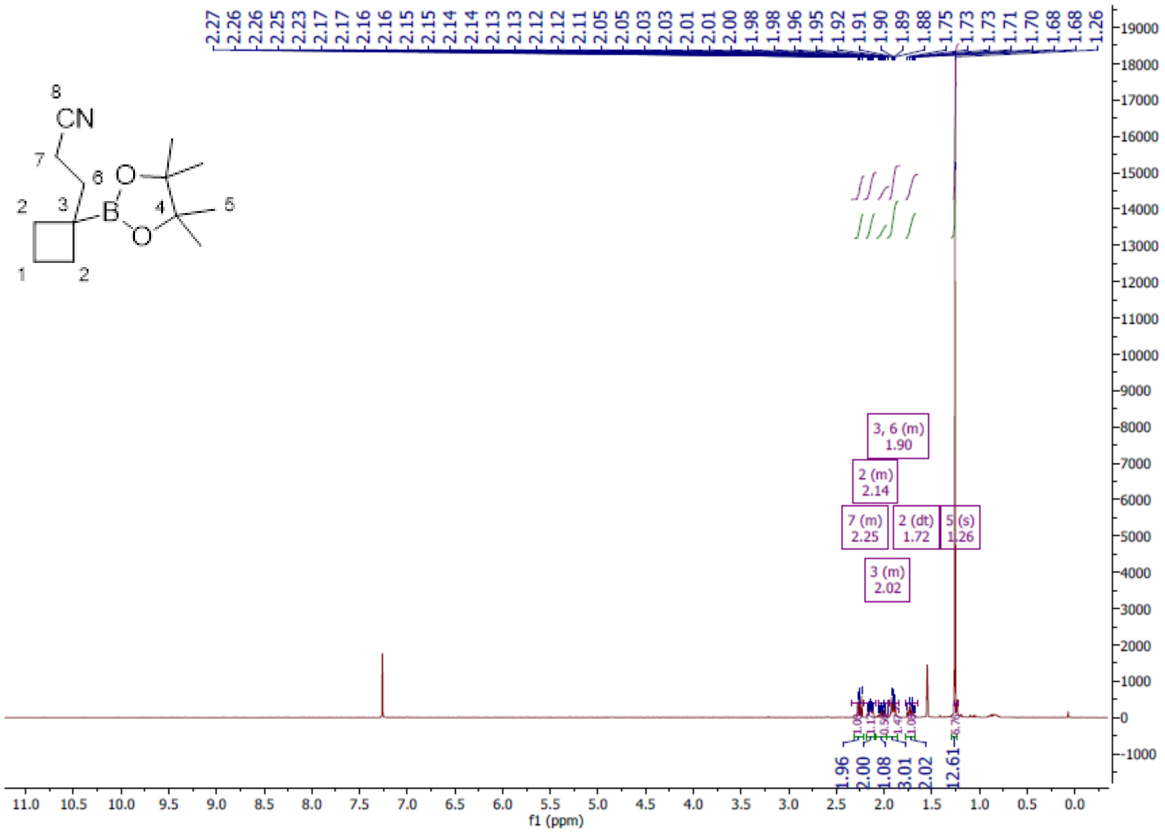

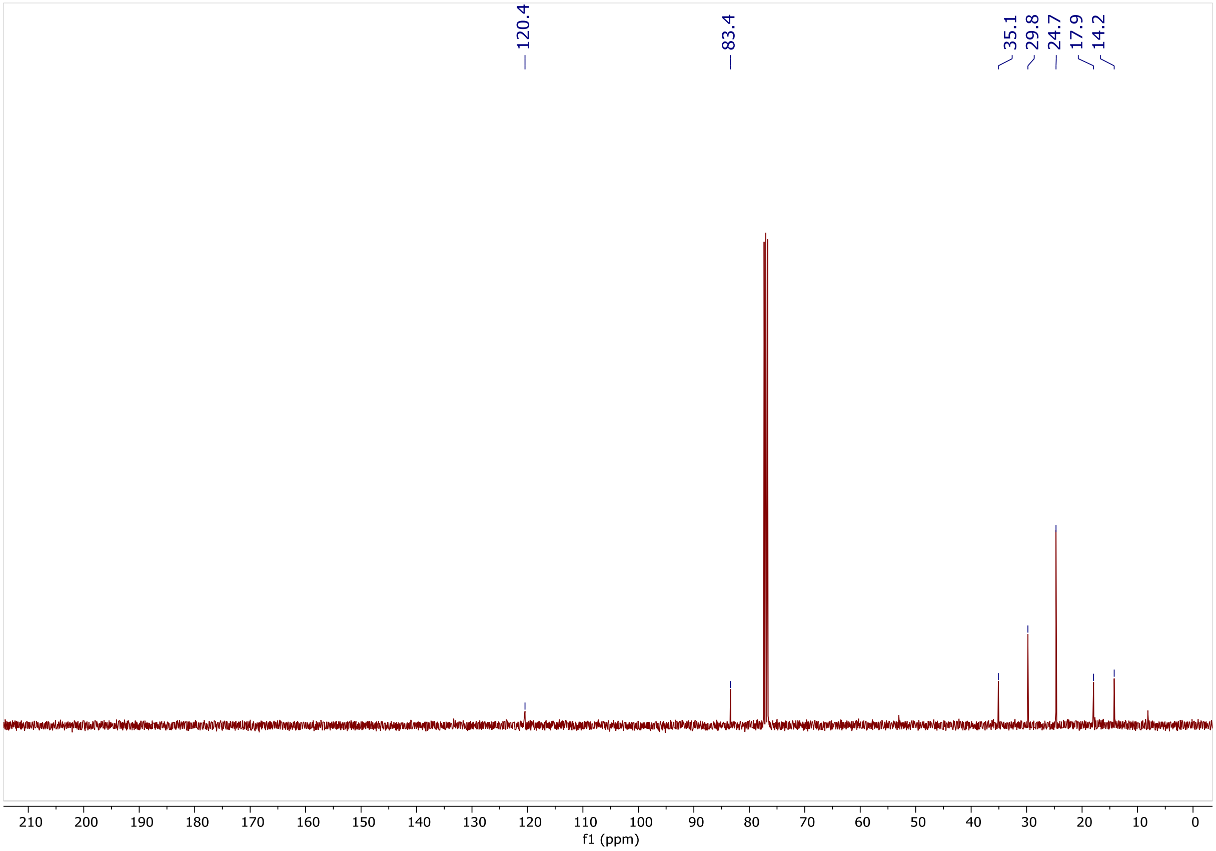

Return to contents

11 tert-Butyl 3-(1-(4,4,5,5-tetramethyl-1,3,2-dioxaborolan-2-yl)cyclobutyl)propanoate

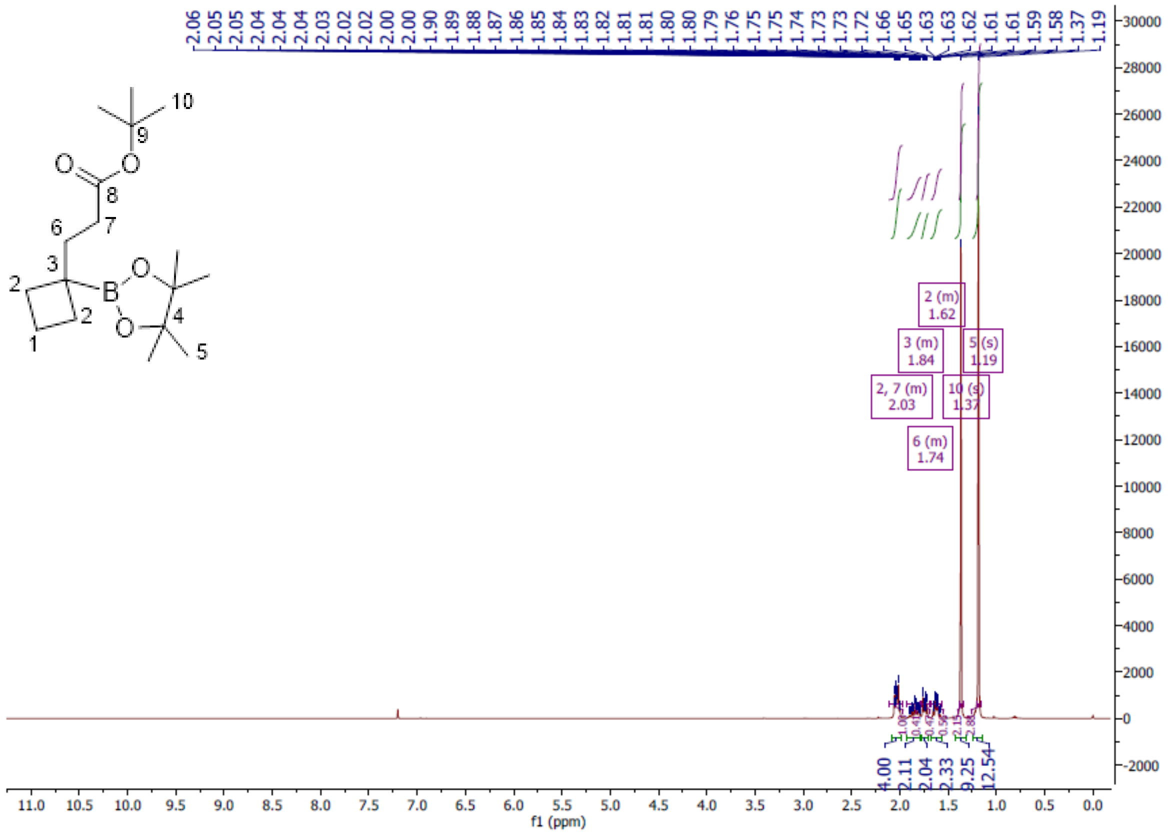

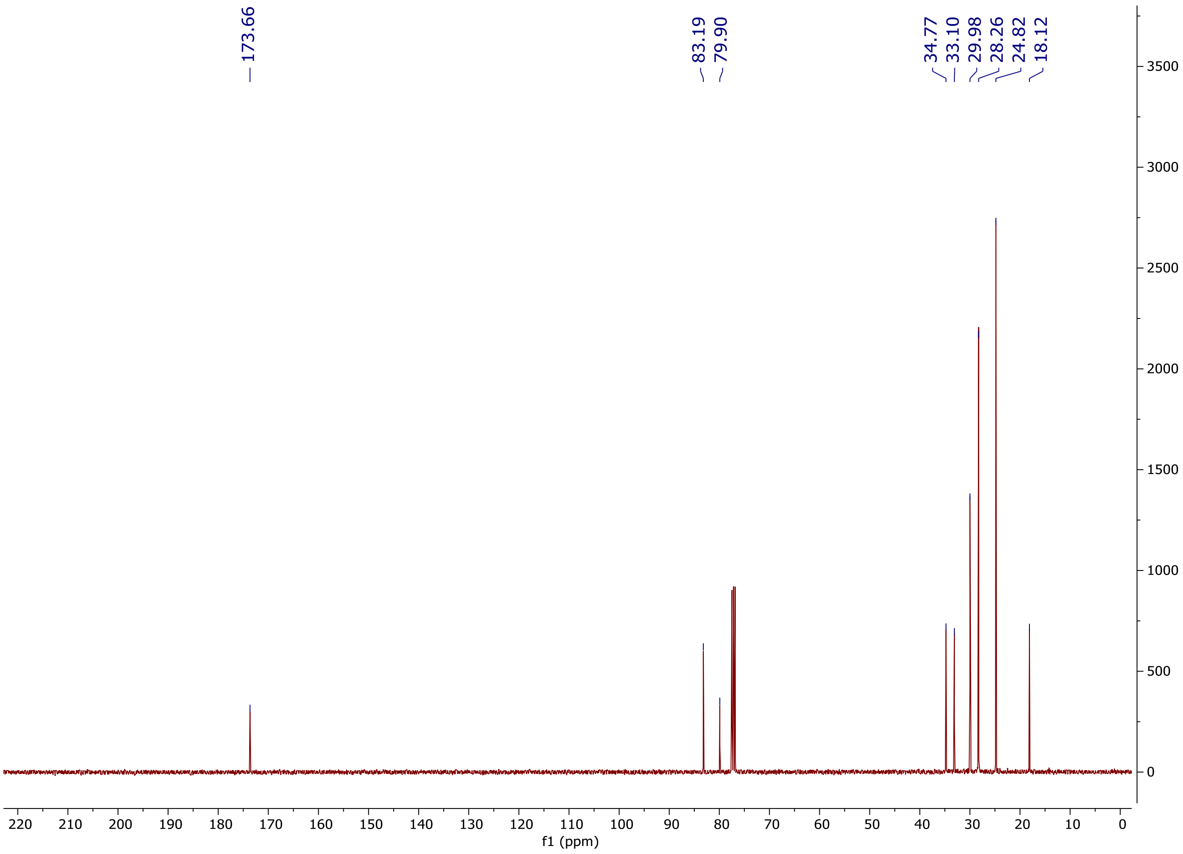

Return to contents

12 2-(1-(3-azidopropyl)cyclobutyl)-4,4,5,5-tetramethyl-1,3,2-dioxaborolane

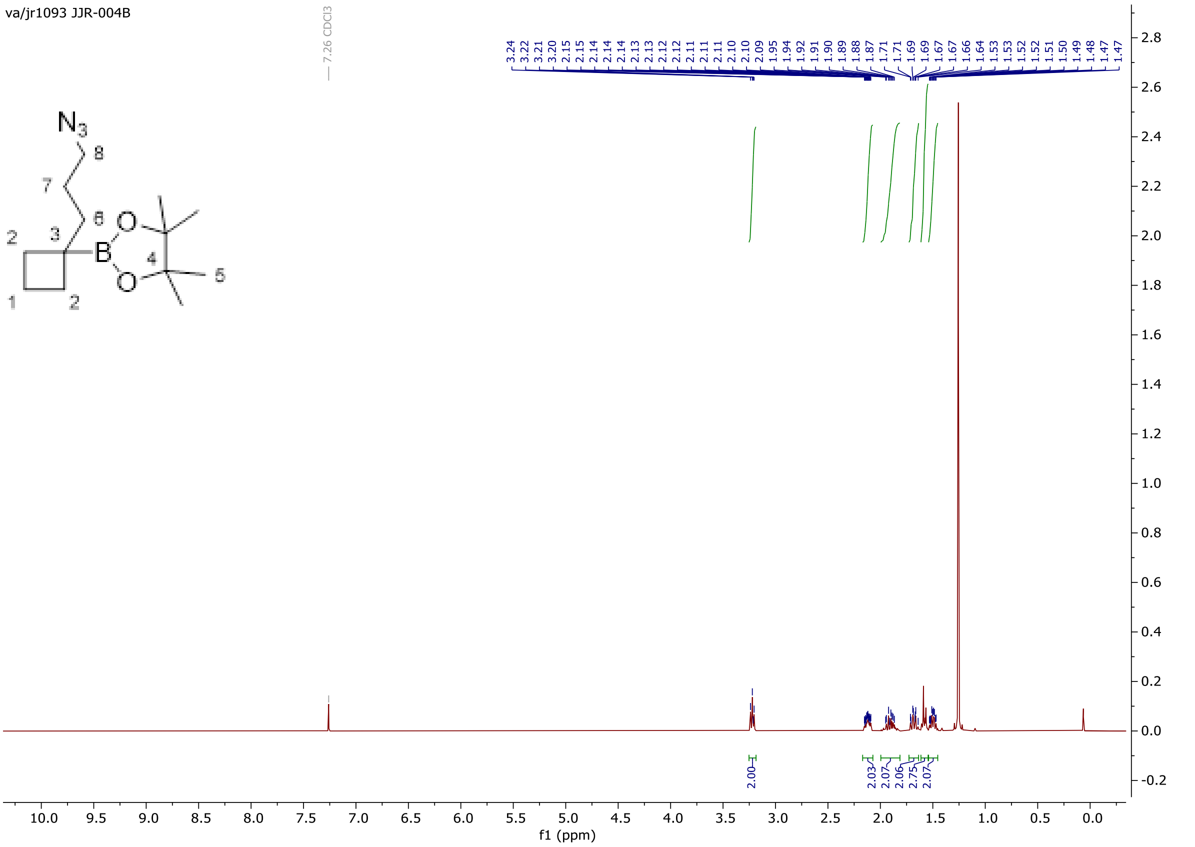

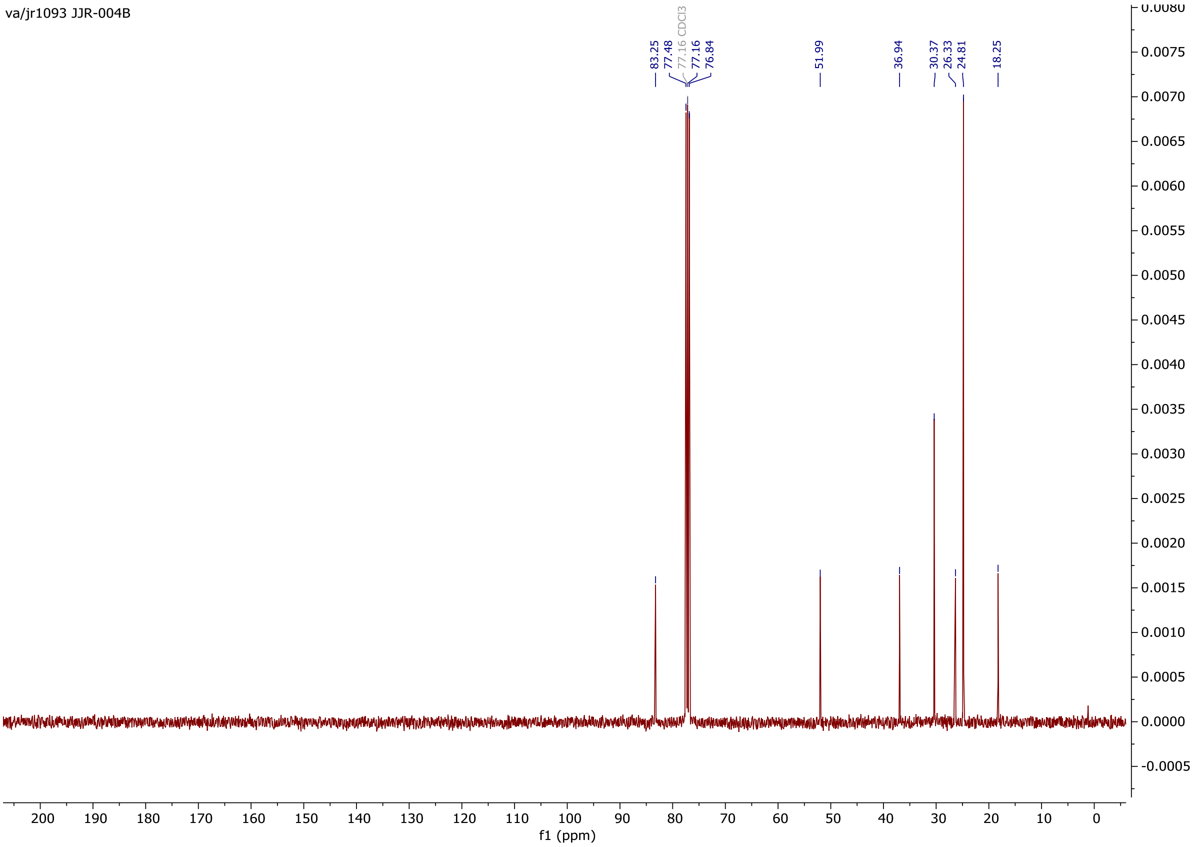

Return to contents

13 1-((4R)-4-((3R,8R,9S,10S,13R,14S,17R)-3-((tert-butyldimethylsilyl)oxy)-10,13-dimethylhexadecahydro-1H-cyclopenta[a]phenanthren-17-yl)pentyl)cyclobutan-1-ol (Lithocholic acid derivative)

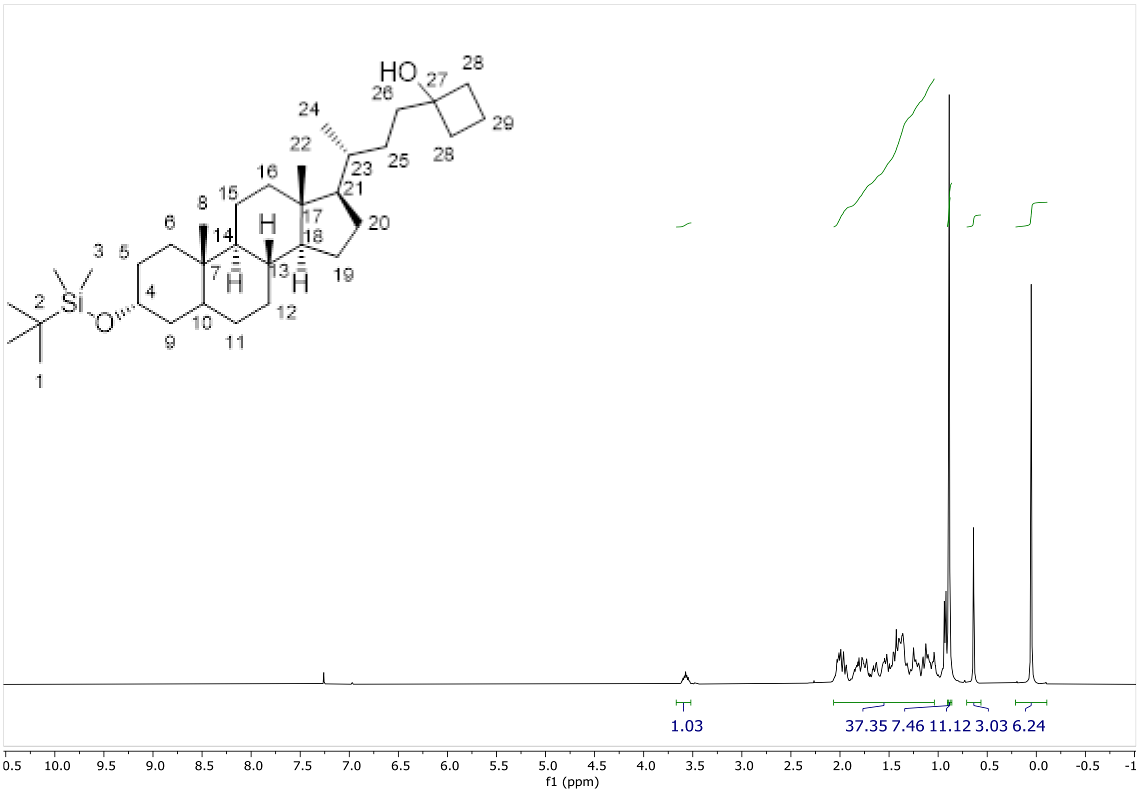

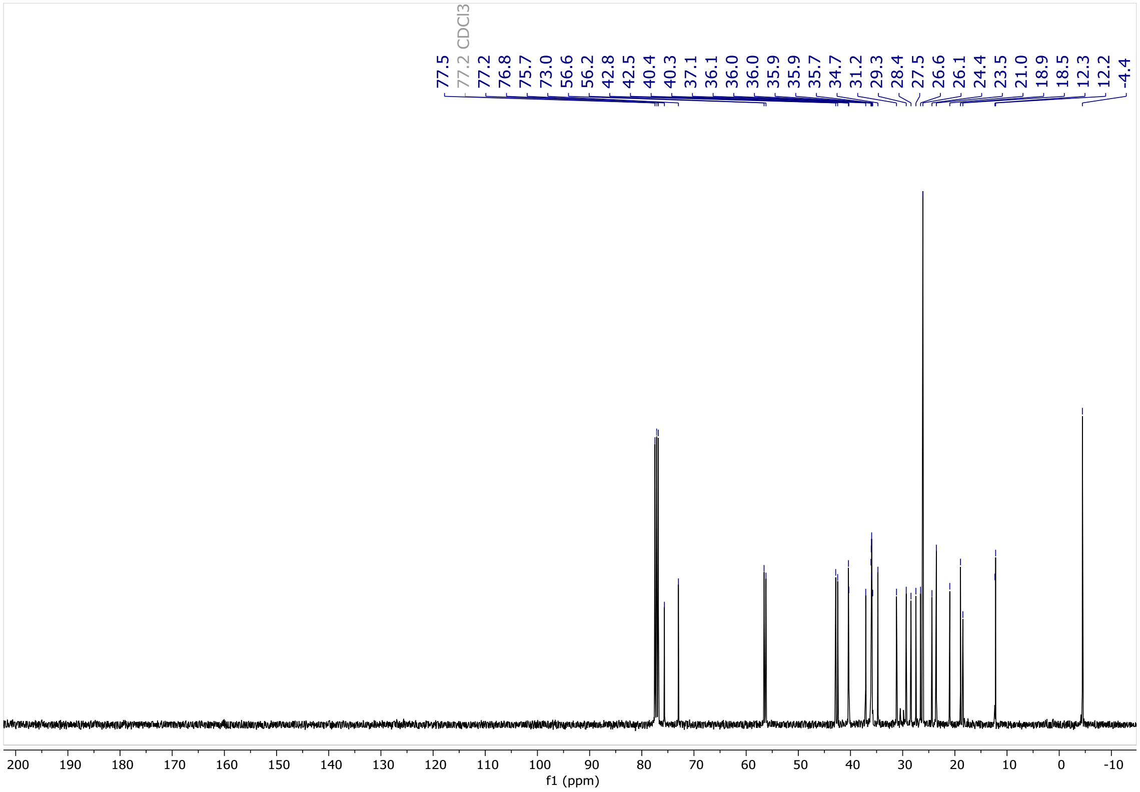

[Return to contents](#)

14 2-(1-cyclohexylcyclobutyl)-4,4,5,5-tetramethyl-1,3,2-dioxaborolane

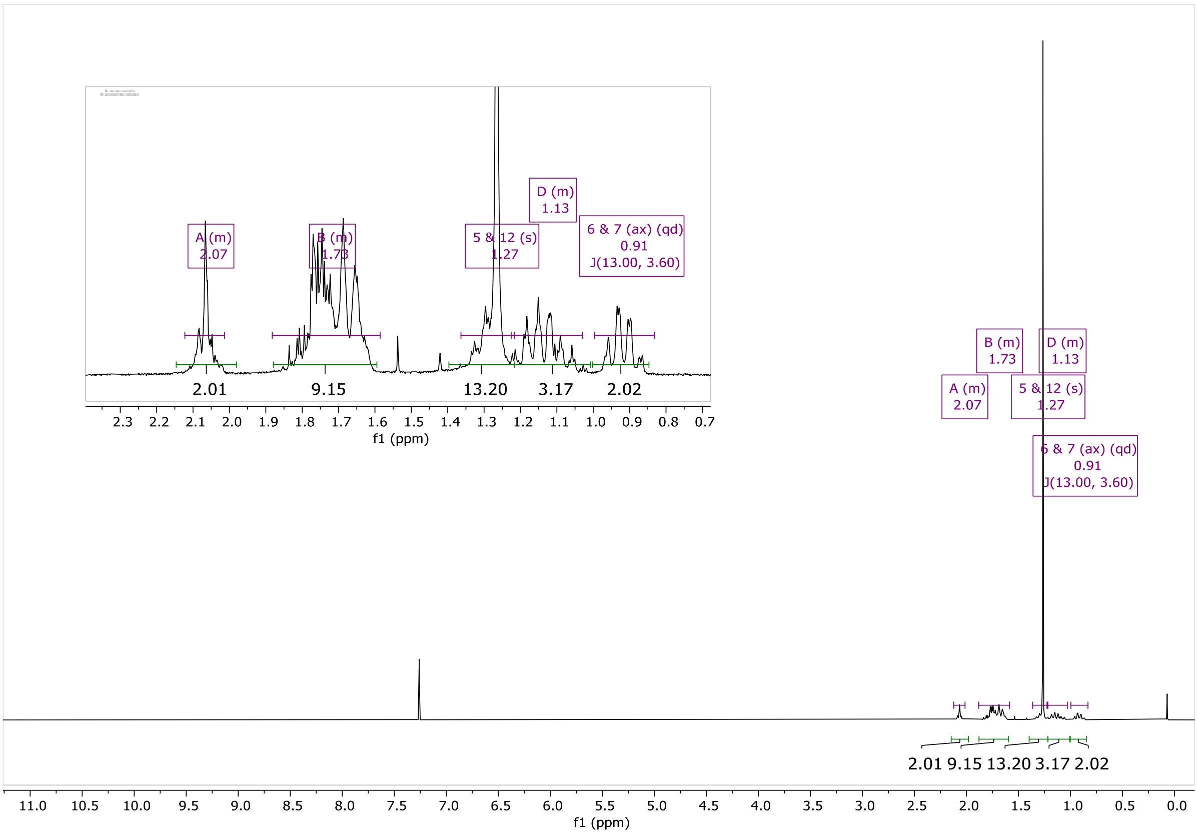

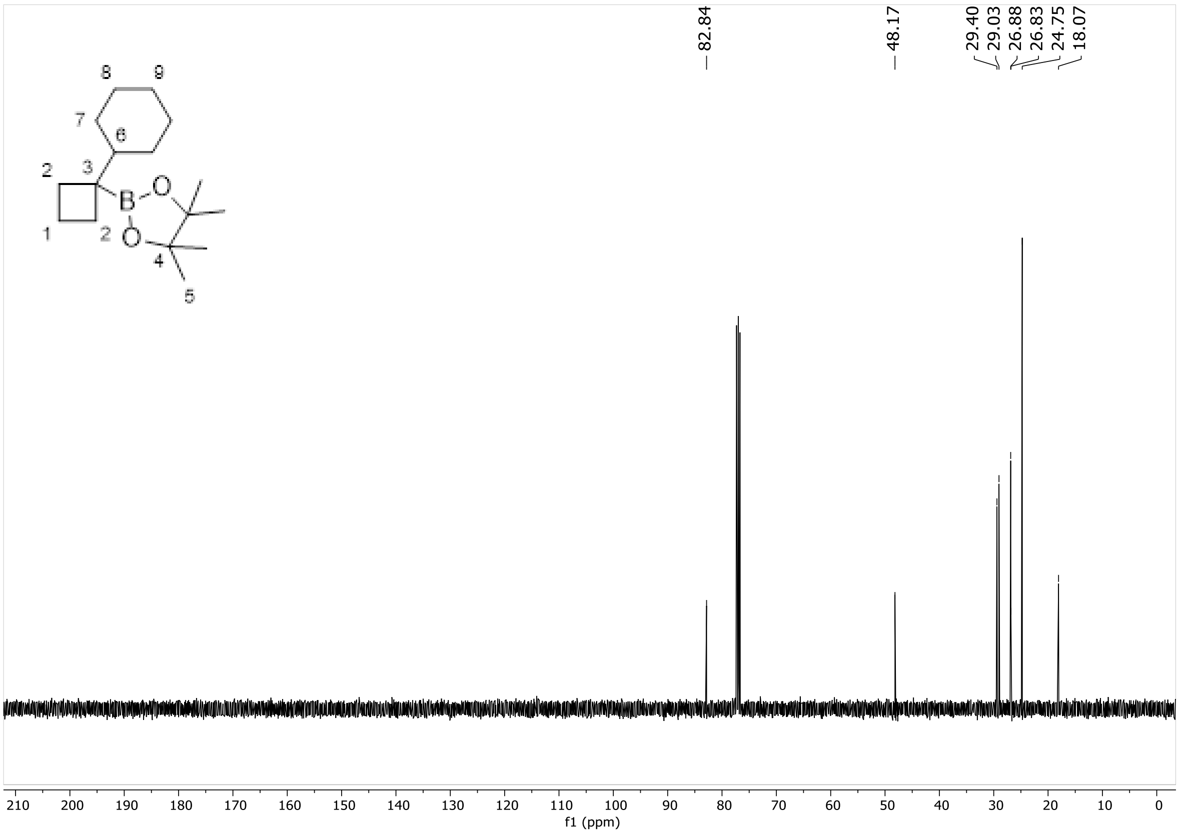

Return to contents

15 1-(1-phenylethyl)cyclobutan-1-ol

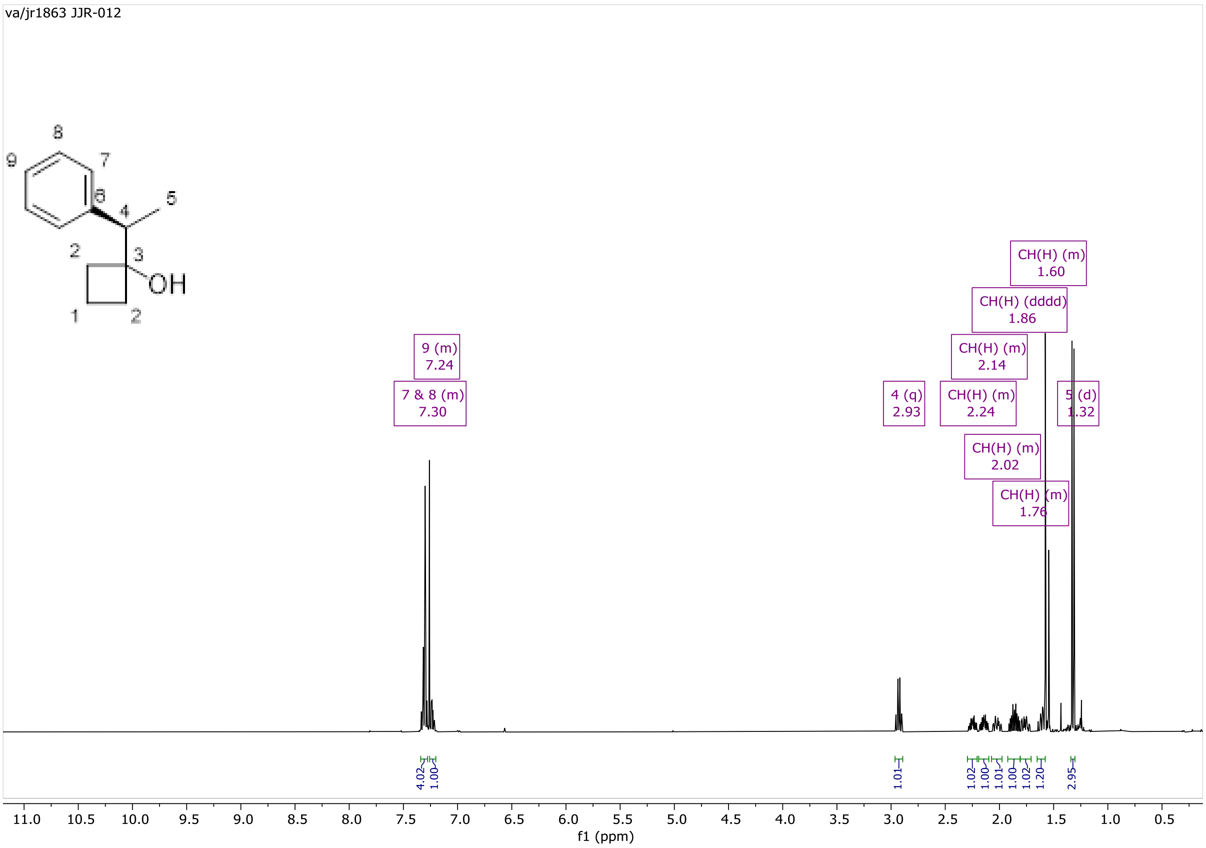

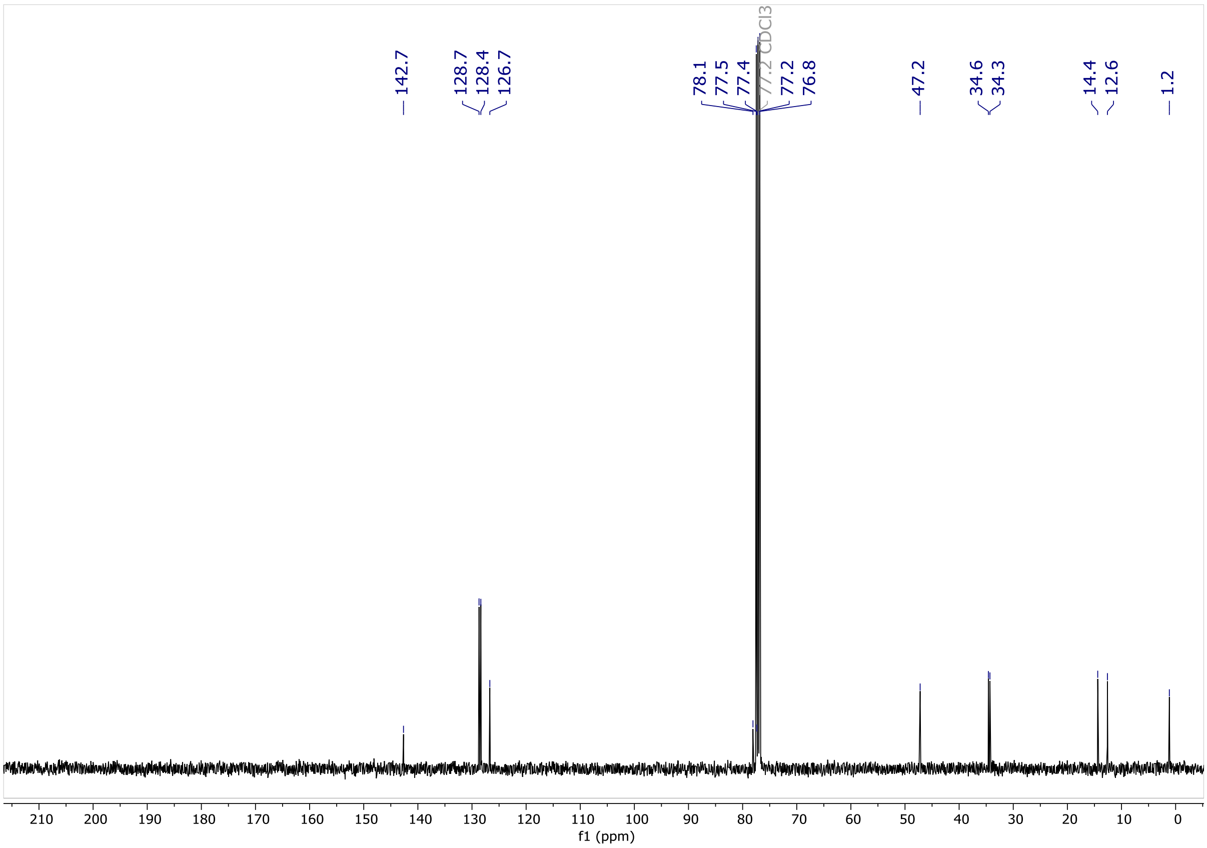

[Return to contents](#)

16 (*R*)-2-(1-(4-(4-methoxyphenyl)butan-2-yl)cyclobutyl)-4,4,5,5-tetramethyl-1,3,2-dioxaborolane

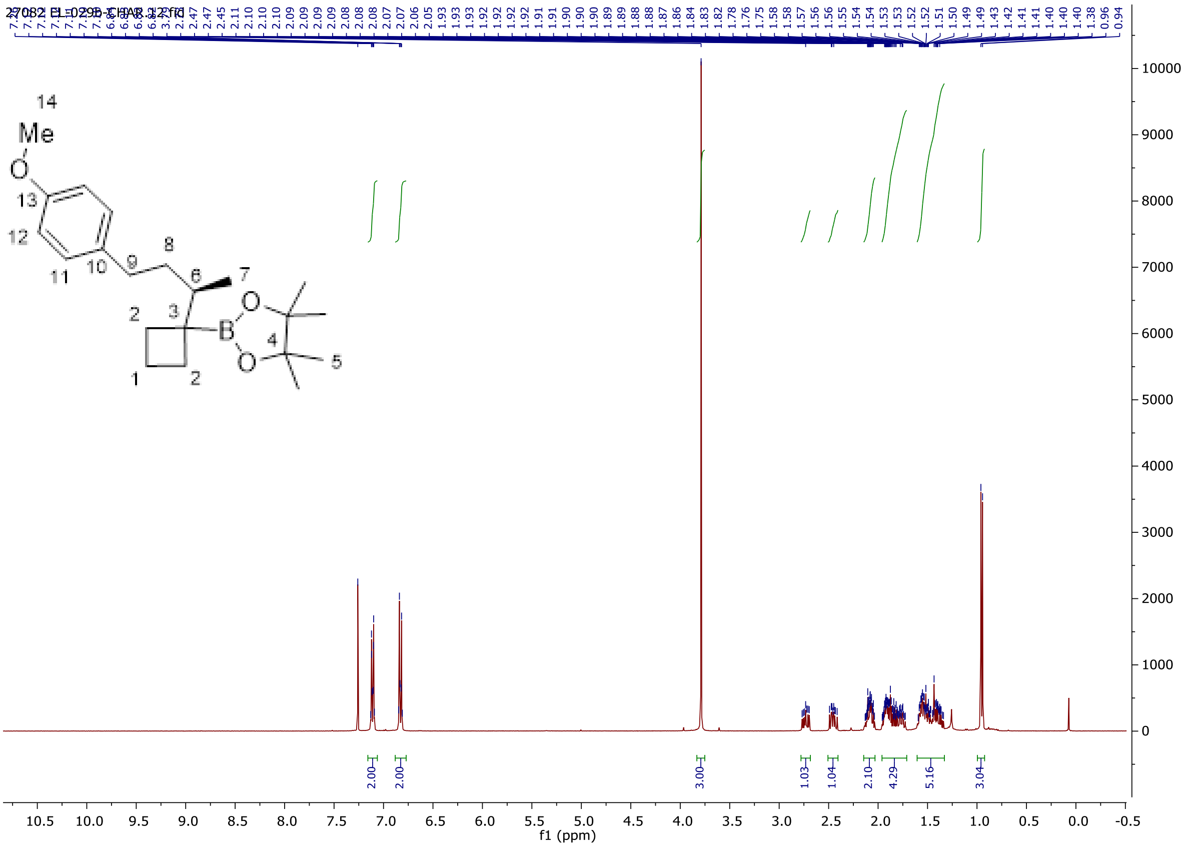

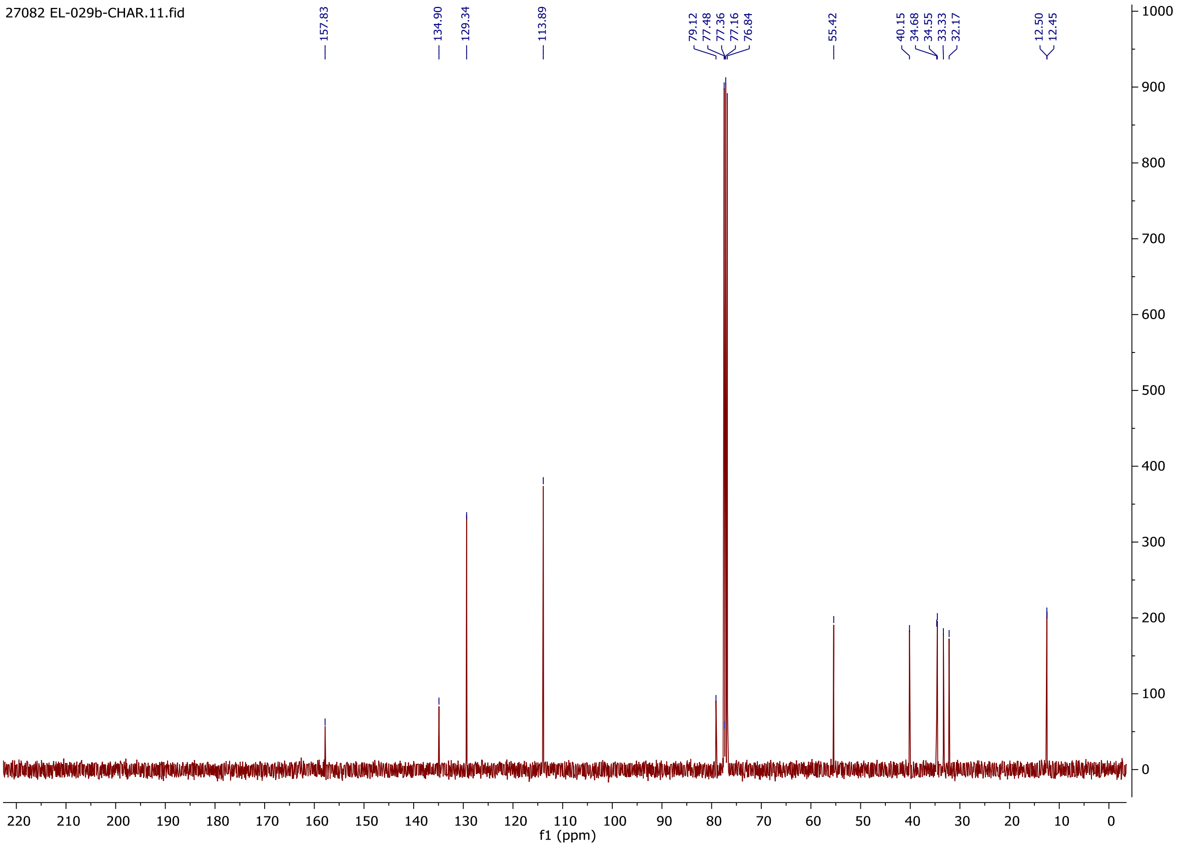

Return to contents

17 4,4,5,5-tetramethyl-2-(*trans*-2-phenylcyclopropyl)cyclobutyl)-1,3,2-dioxaborolane

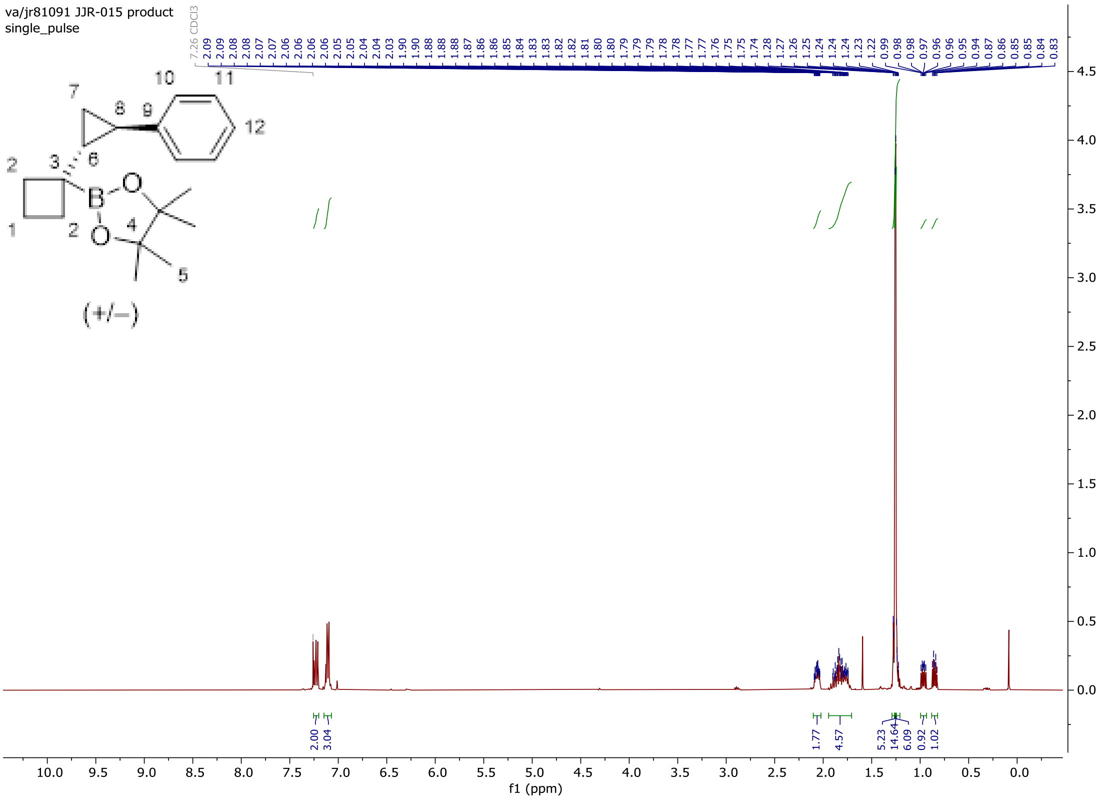

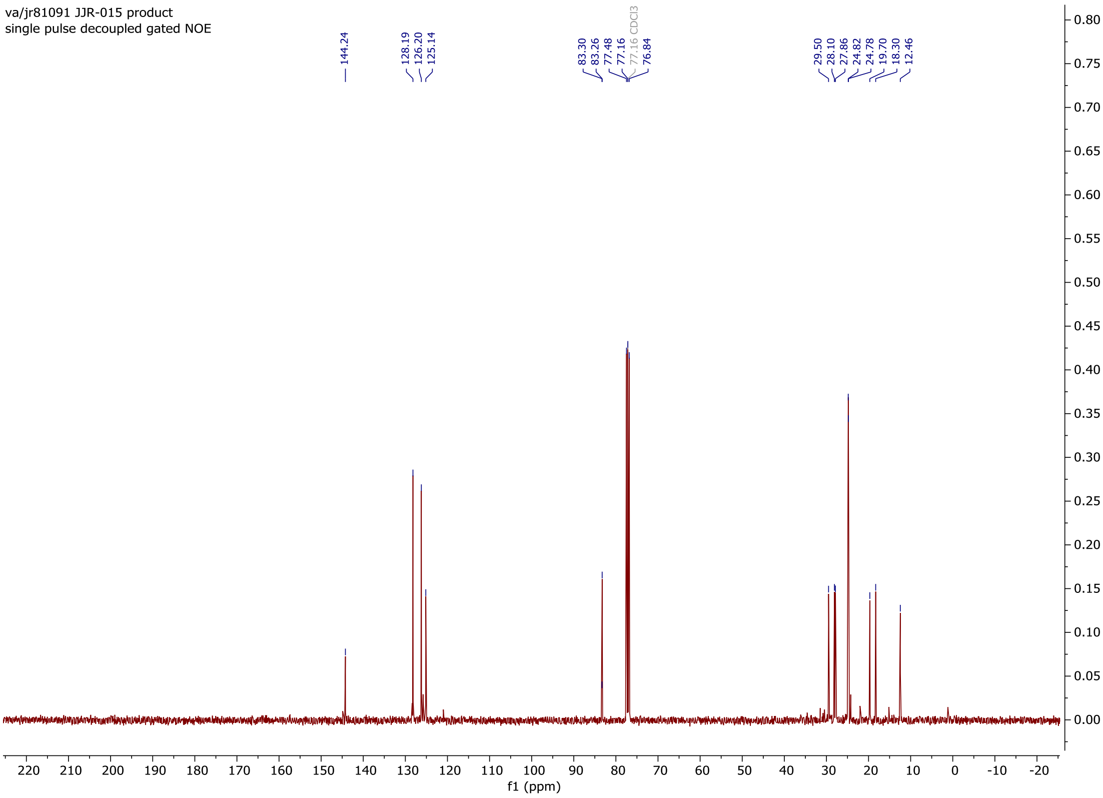

Return to contents

18 tert-butyl 2-(1-(4,4,5,5-tetramethyl-1,3,2-dioxaborolan-2-yl)cyclobutyl)pyrrolidine-1-carboxylate

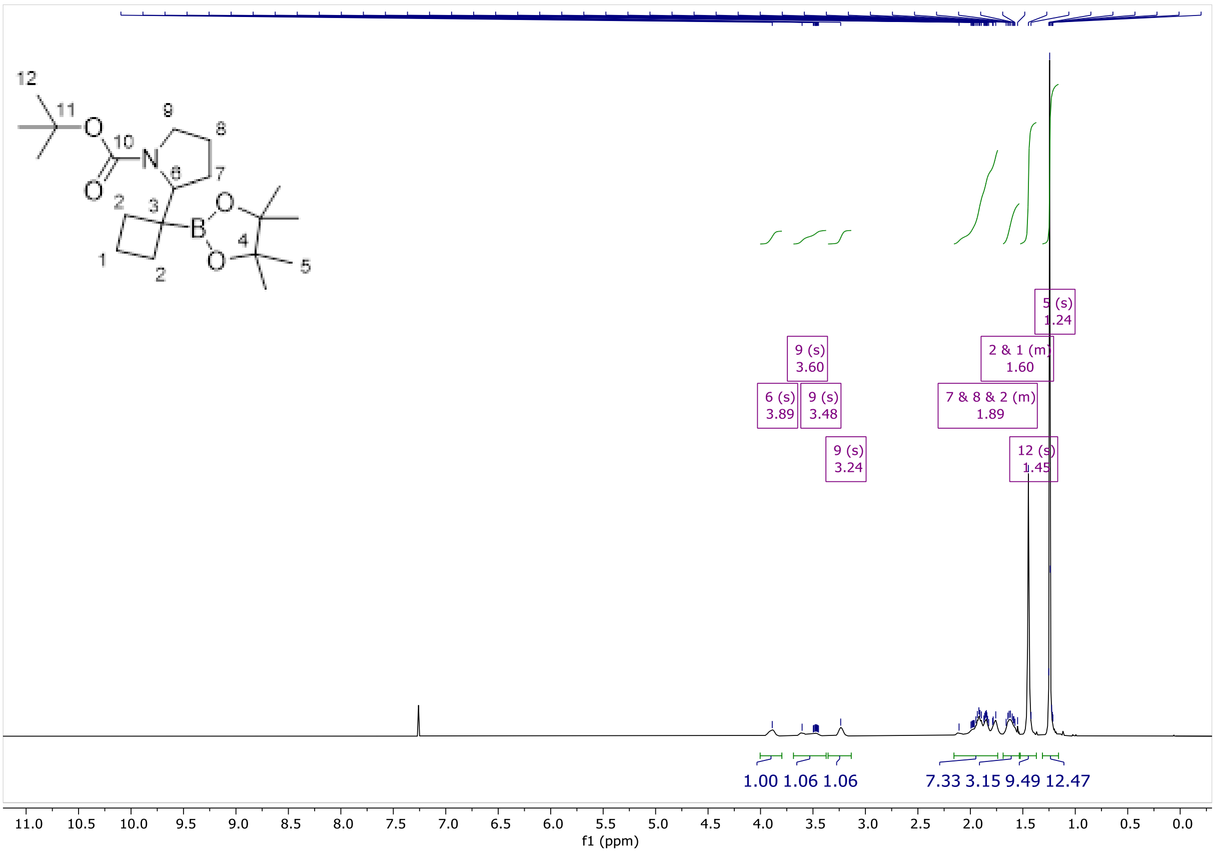

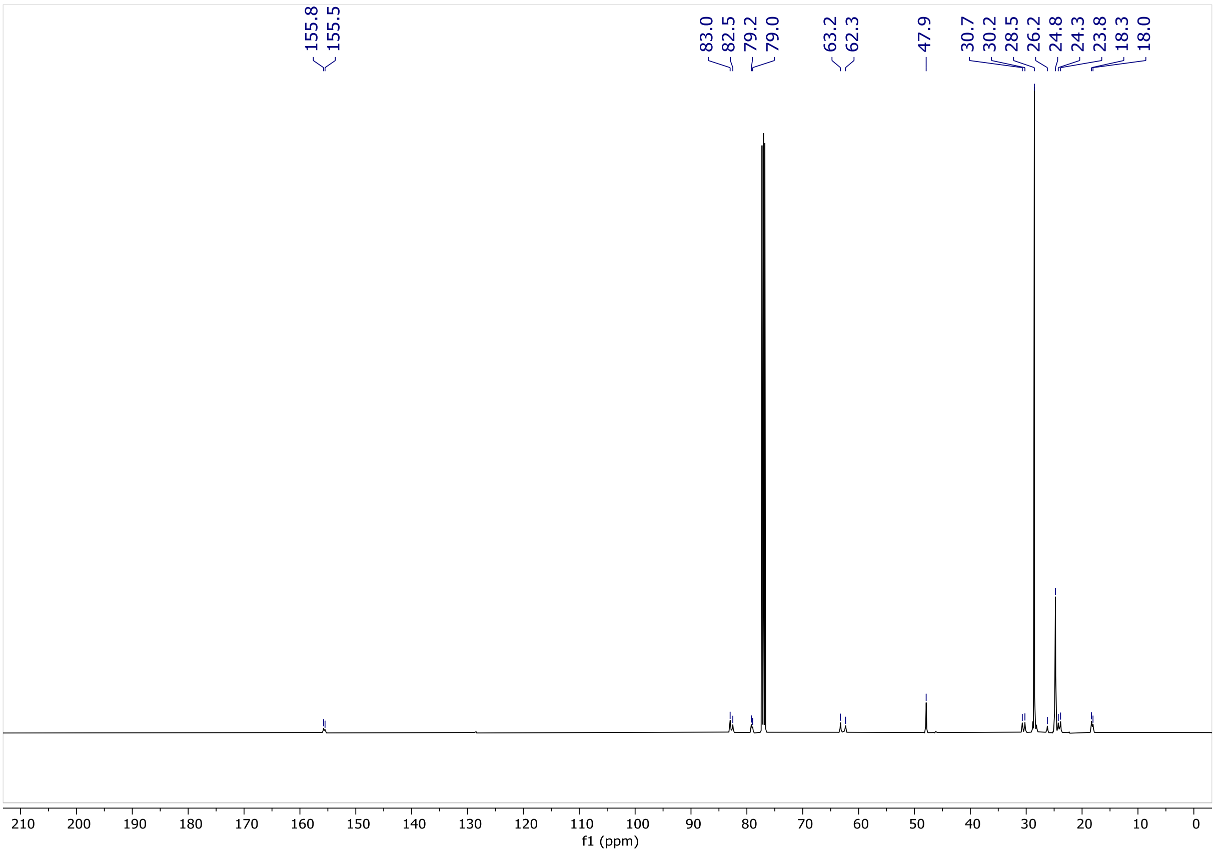

[Return to contents](#)

19 tert-butyl 4-(1-(4,4,5,5-tetramethyl-1,3,2-dioxaborolan-2-yl)cyclobutyl)piperidine-1-carboxylate

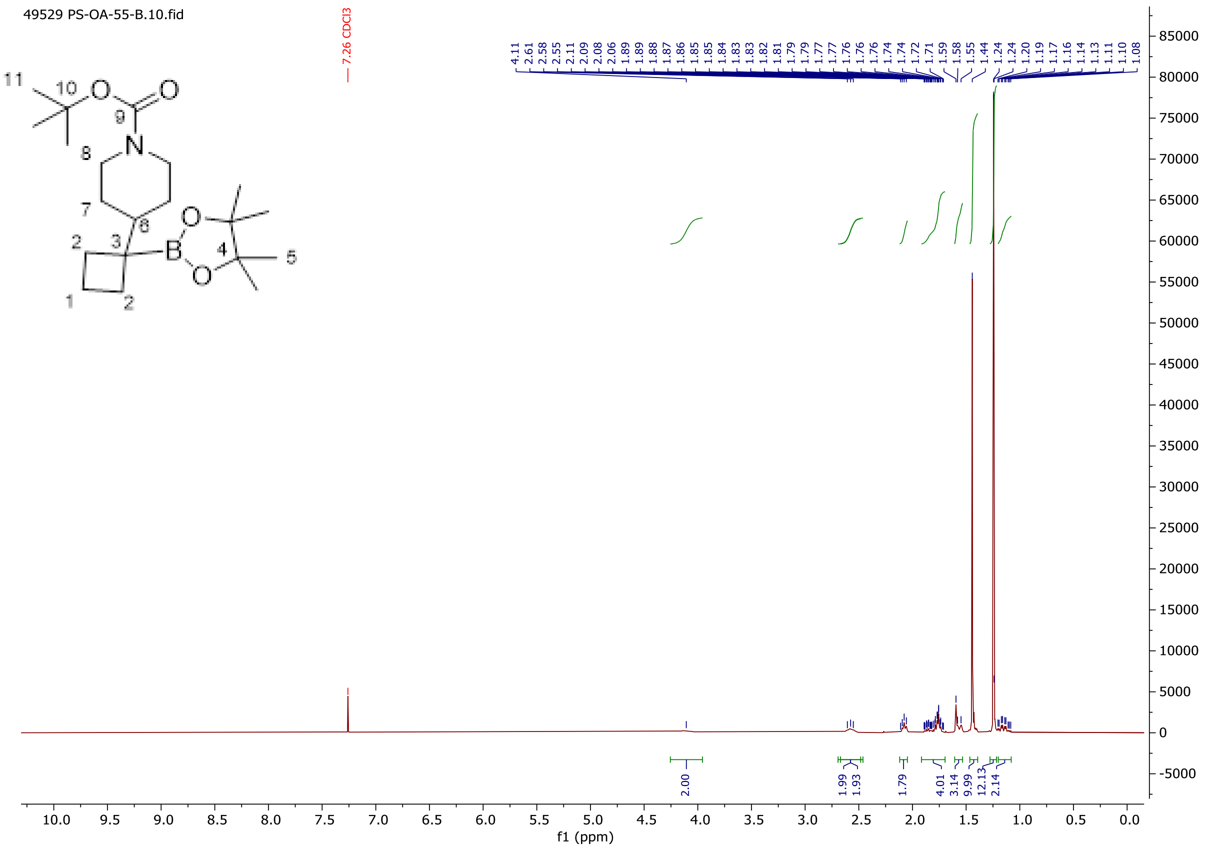

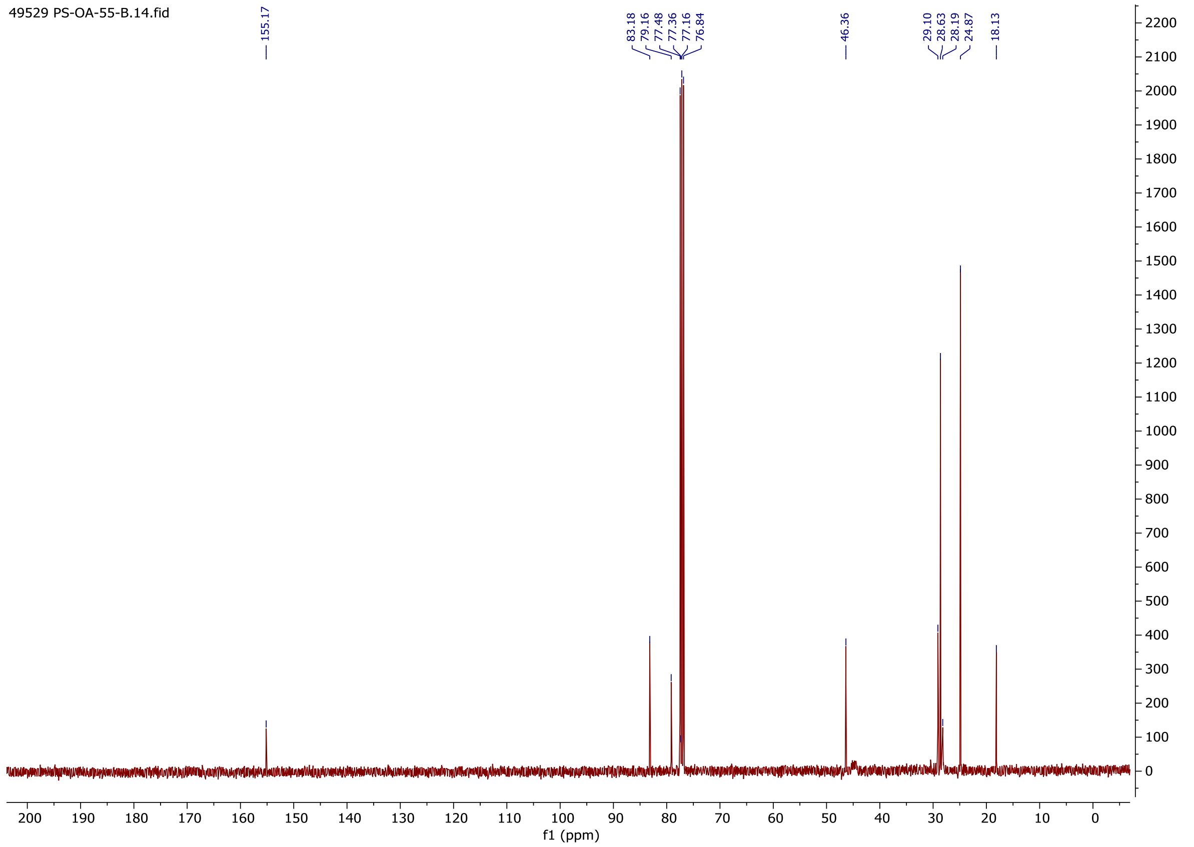

Return to contents

20 2-(1-((1*R*,2*S*,5*R*)-2-isopropyl-5-methylcyclohexyl)cyclobutyl)-5,5-dimethyl-1,3,2-dioxaborinane

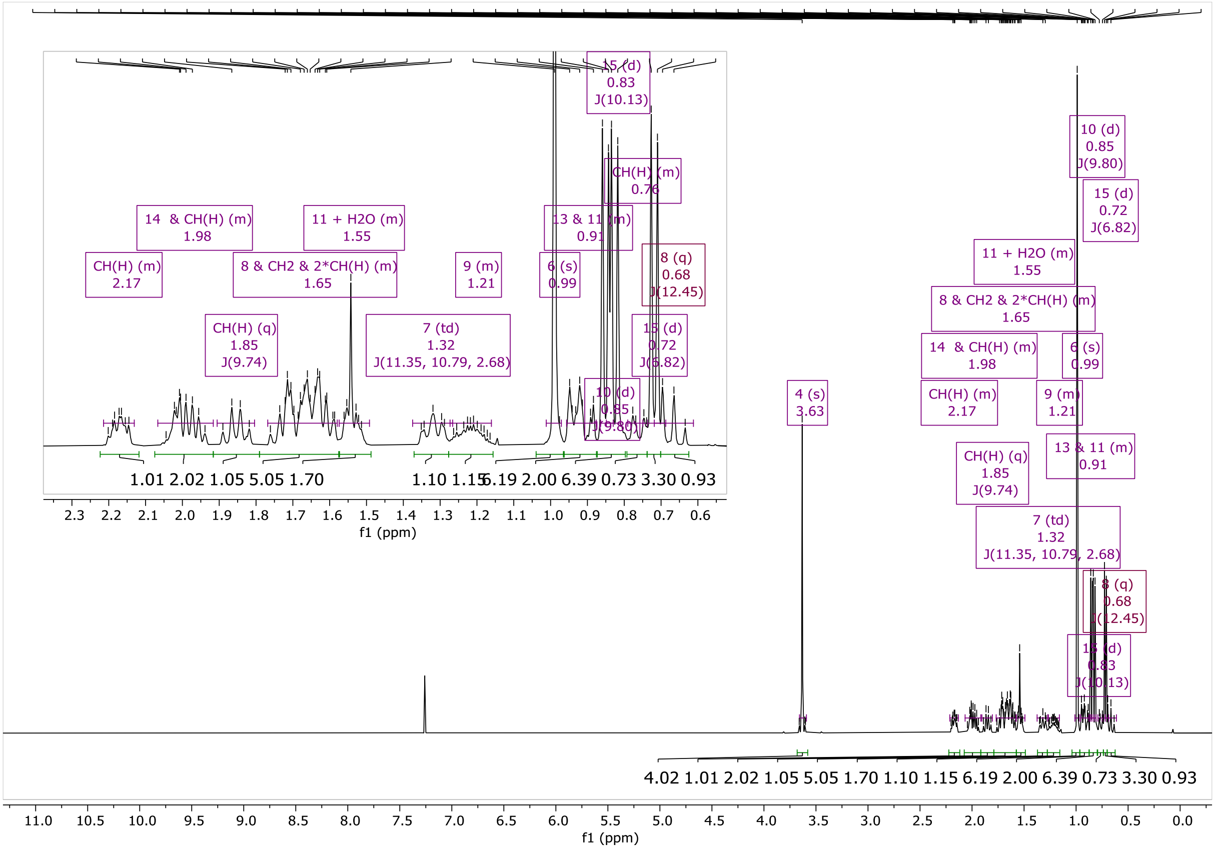

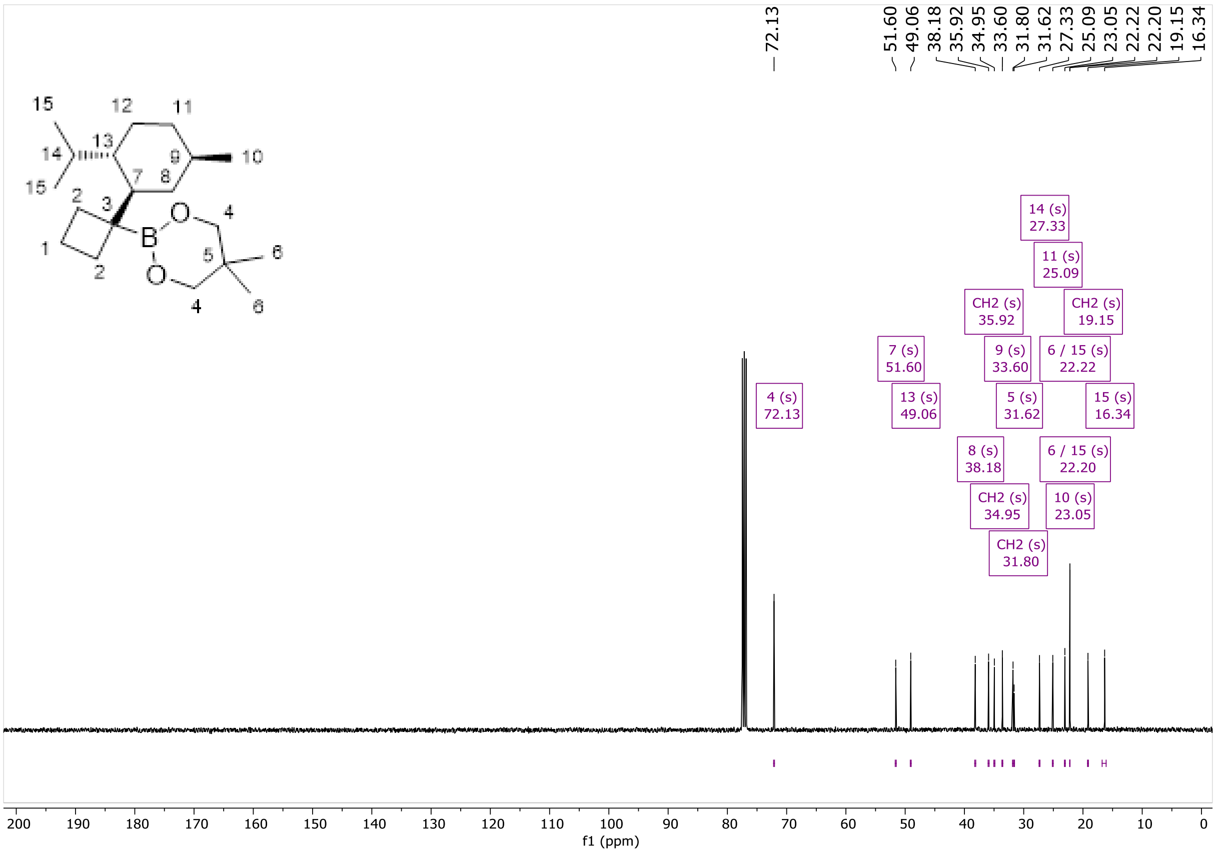

Return to contents

21 tert-butyl 2-(1-(4,4,5,5-tetramethyl-1,3,2-dioxaborolan-2-yl)cyclobutyl)piperidine-1-carboxylate

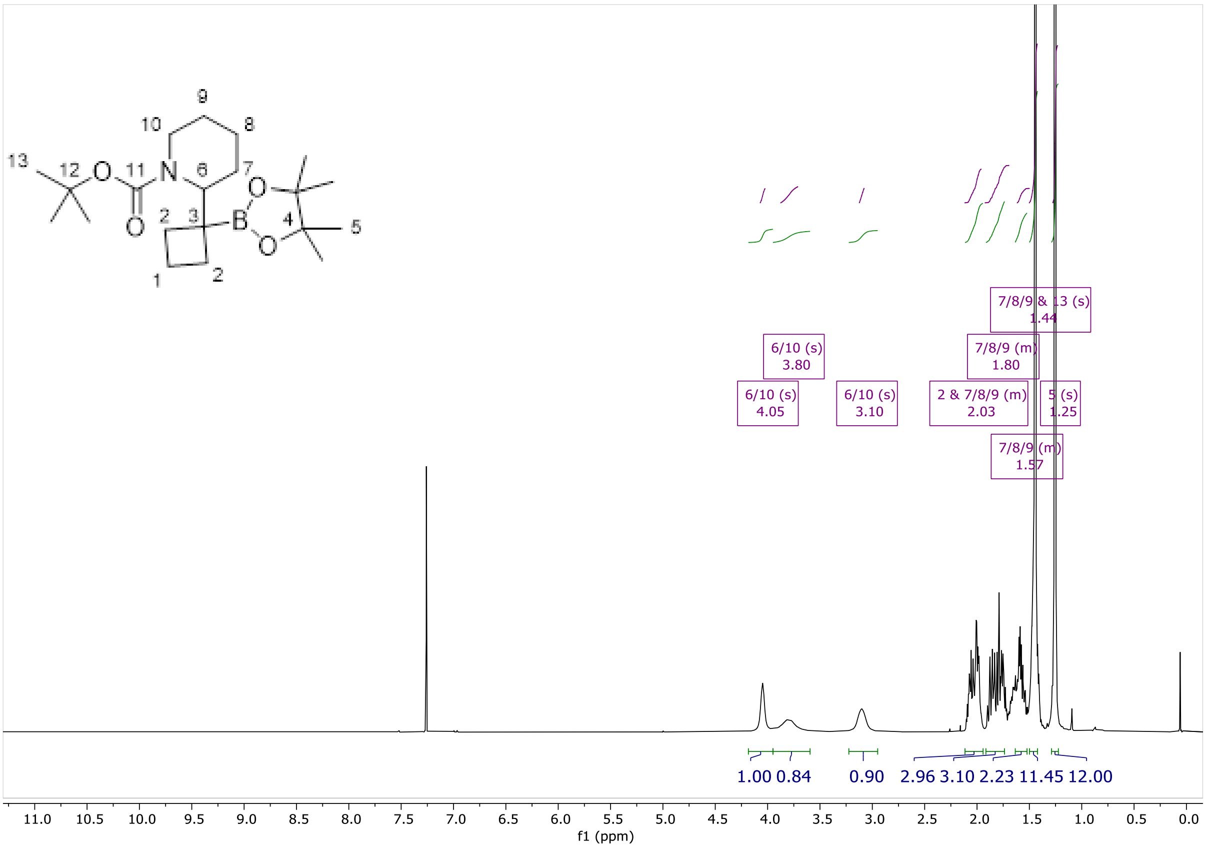

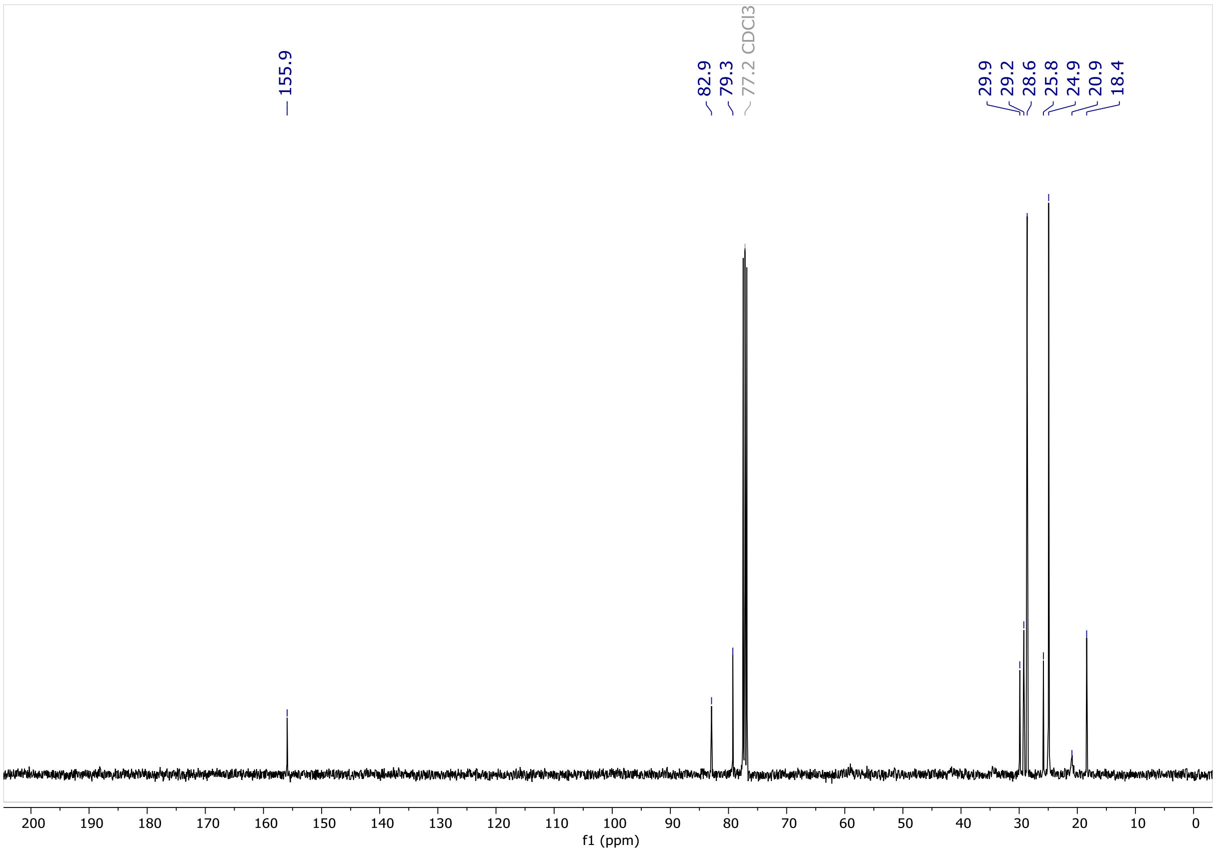

Return to contents

22 4,4,5,5-tetramethyl-2-(1-phenylcyclobutyl)-1,3,2-dioxaborolane

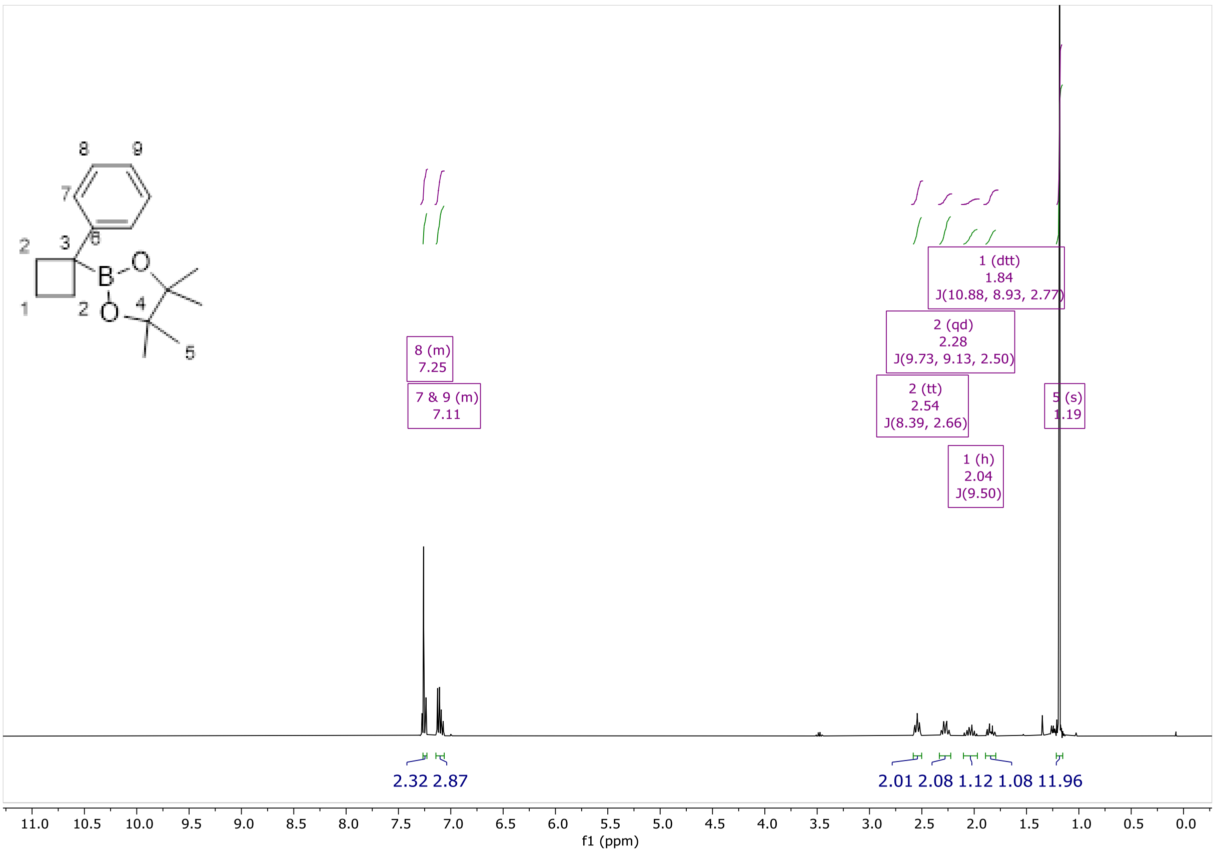

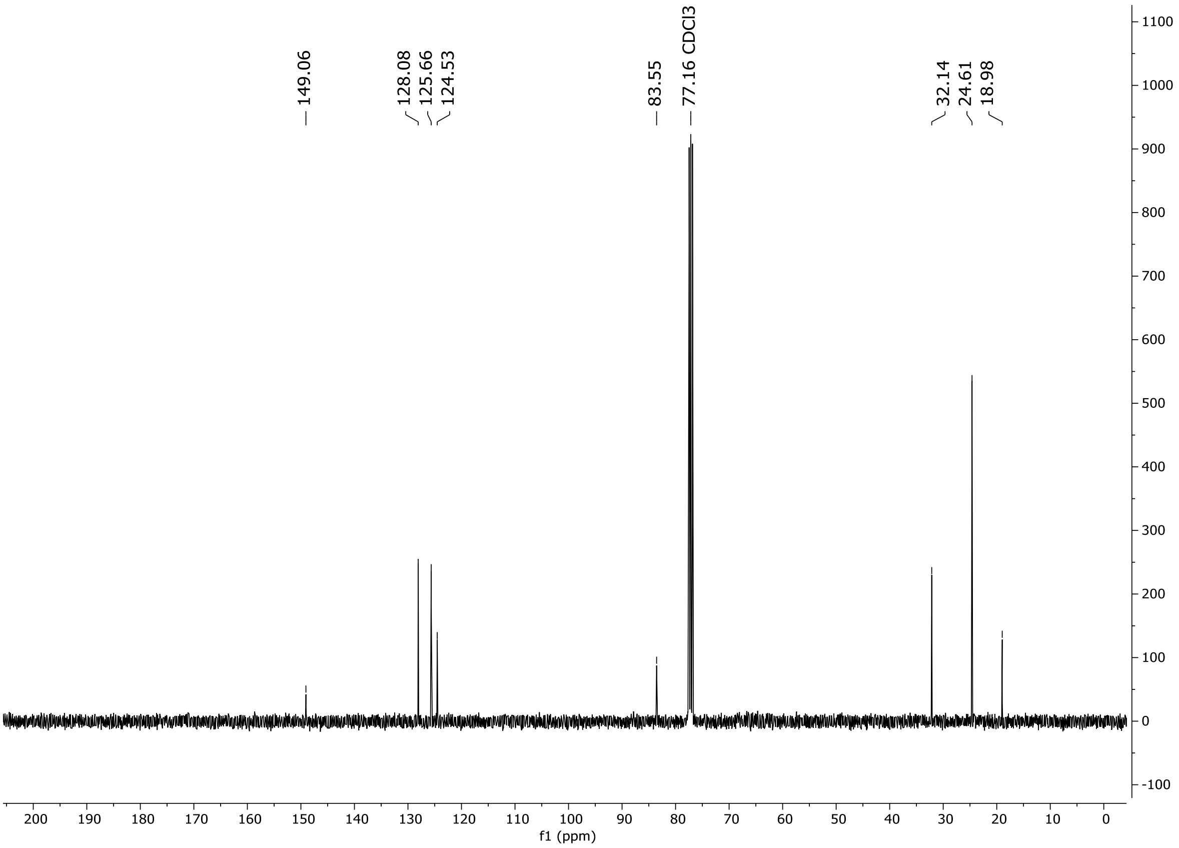

Return to contents

23 1-(4-methoxyphenyl)cyclobutan-1-ol

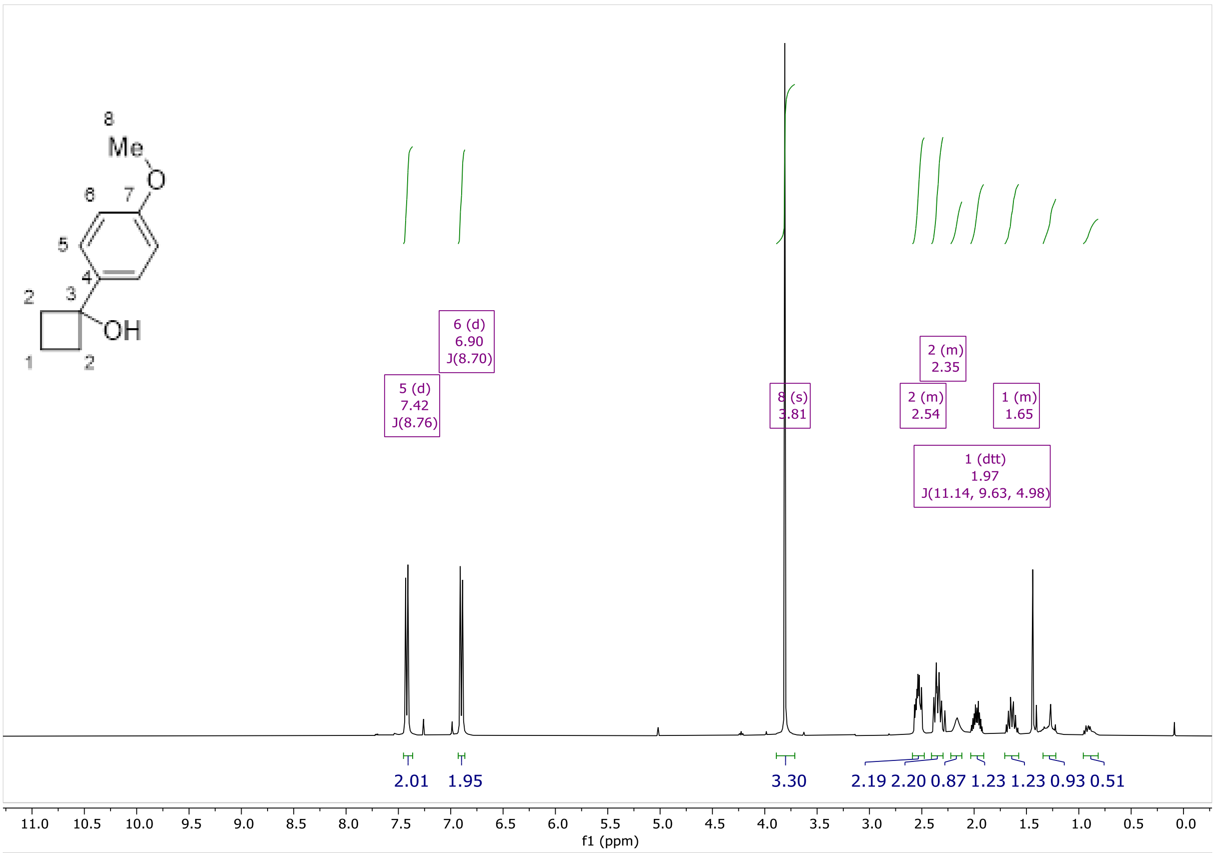

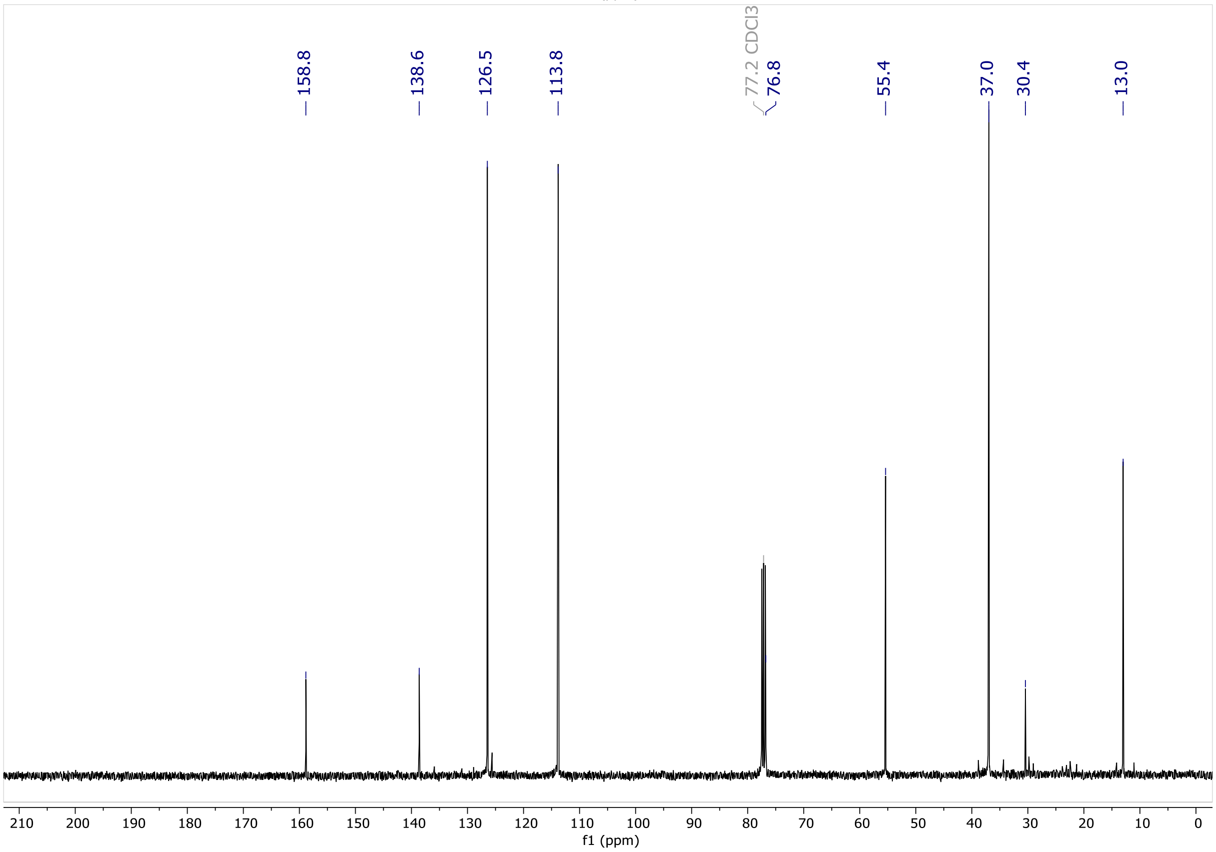

Return to contents

24 2-(1-(4-chlorophenyl)cyclobutyl)-4,4,5,5-tetramethyl-1,3,2-dioxaborolane

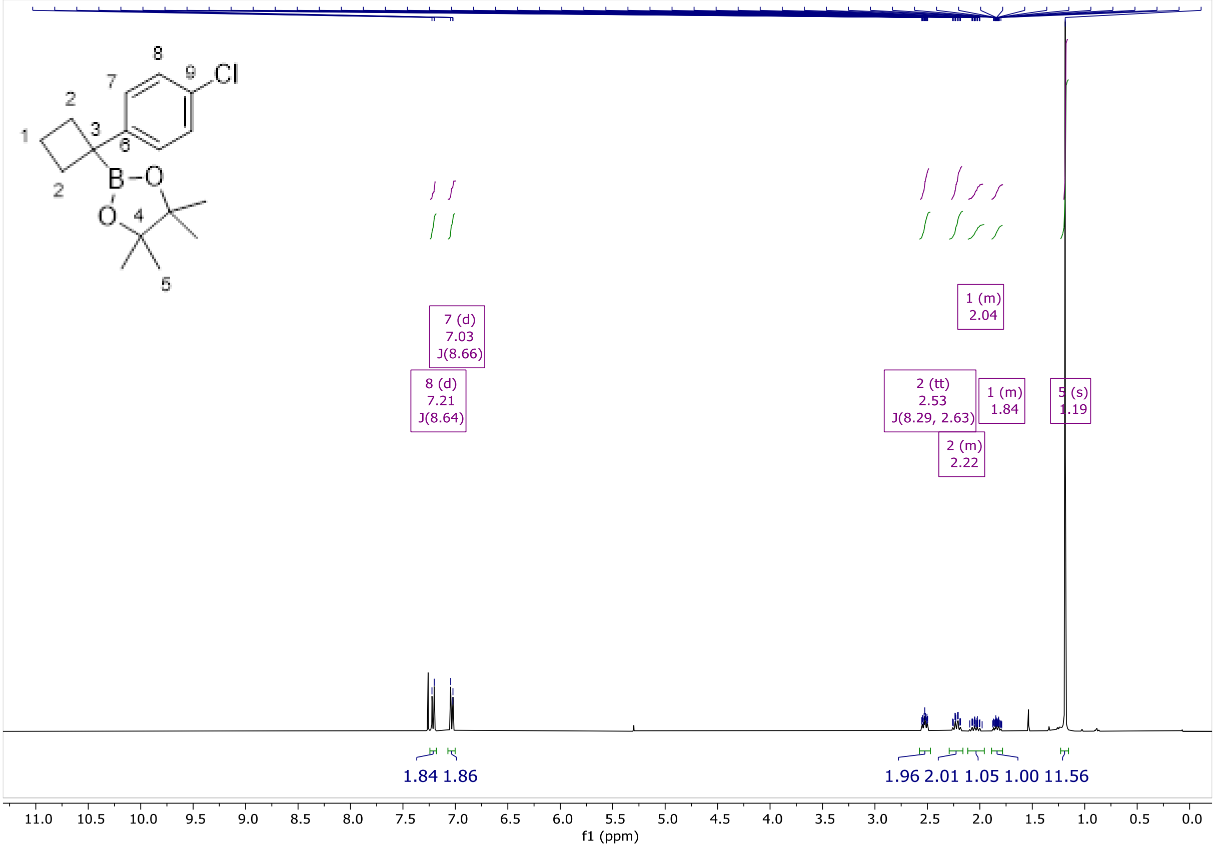

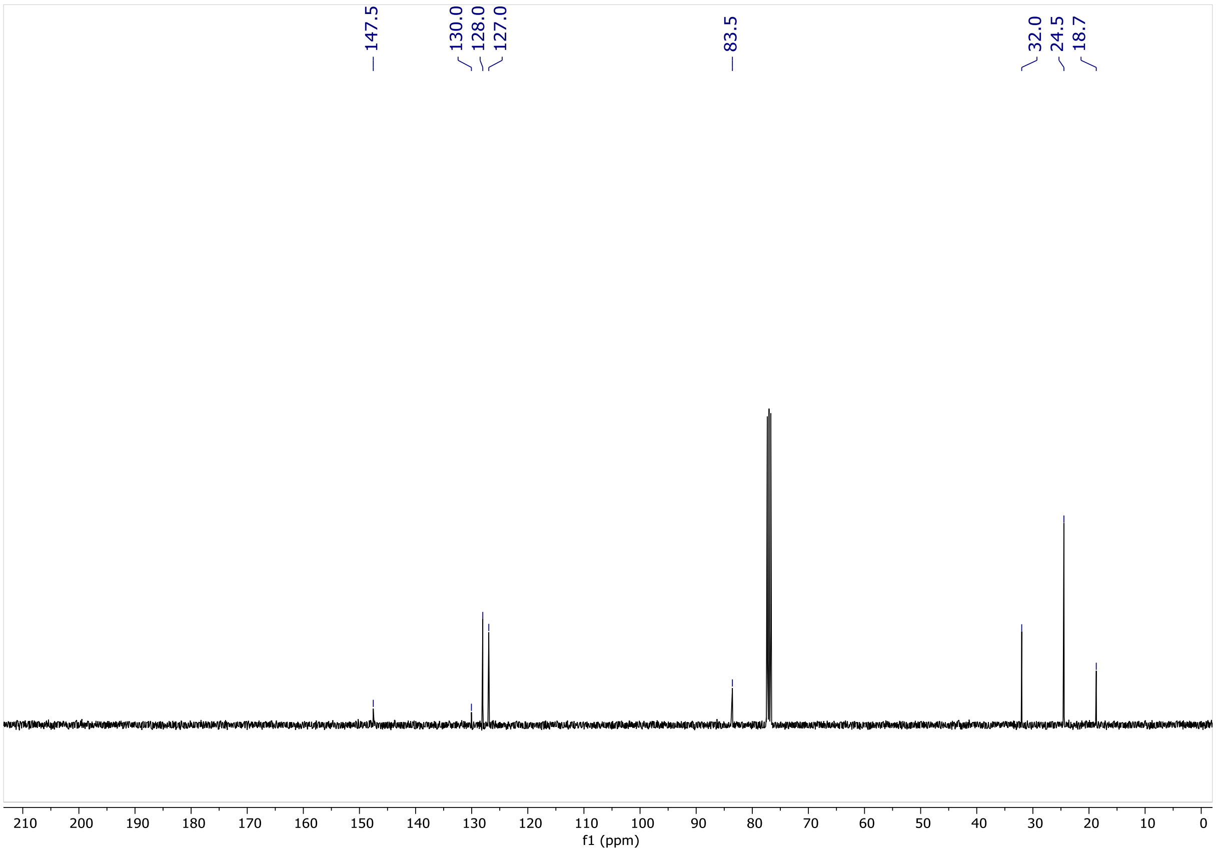

Return to contents

25 *tert*-butyl 4-(1-(4,4,5,5-tetramethyl-1,3,2-dioxaborolan-2-yl)cyclobutyl)-3,6-dihydropyridine-1(2H)-carboxylate

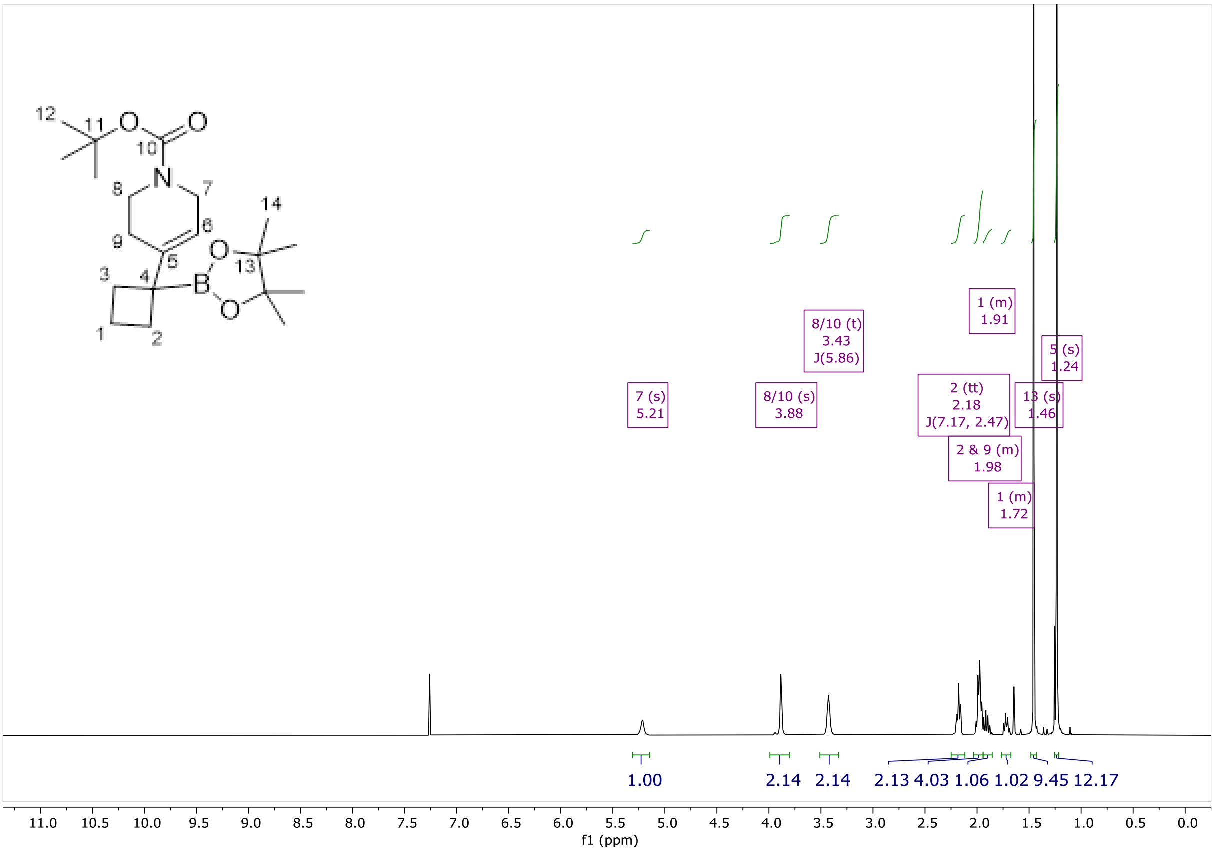

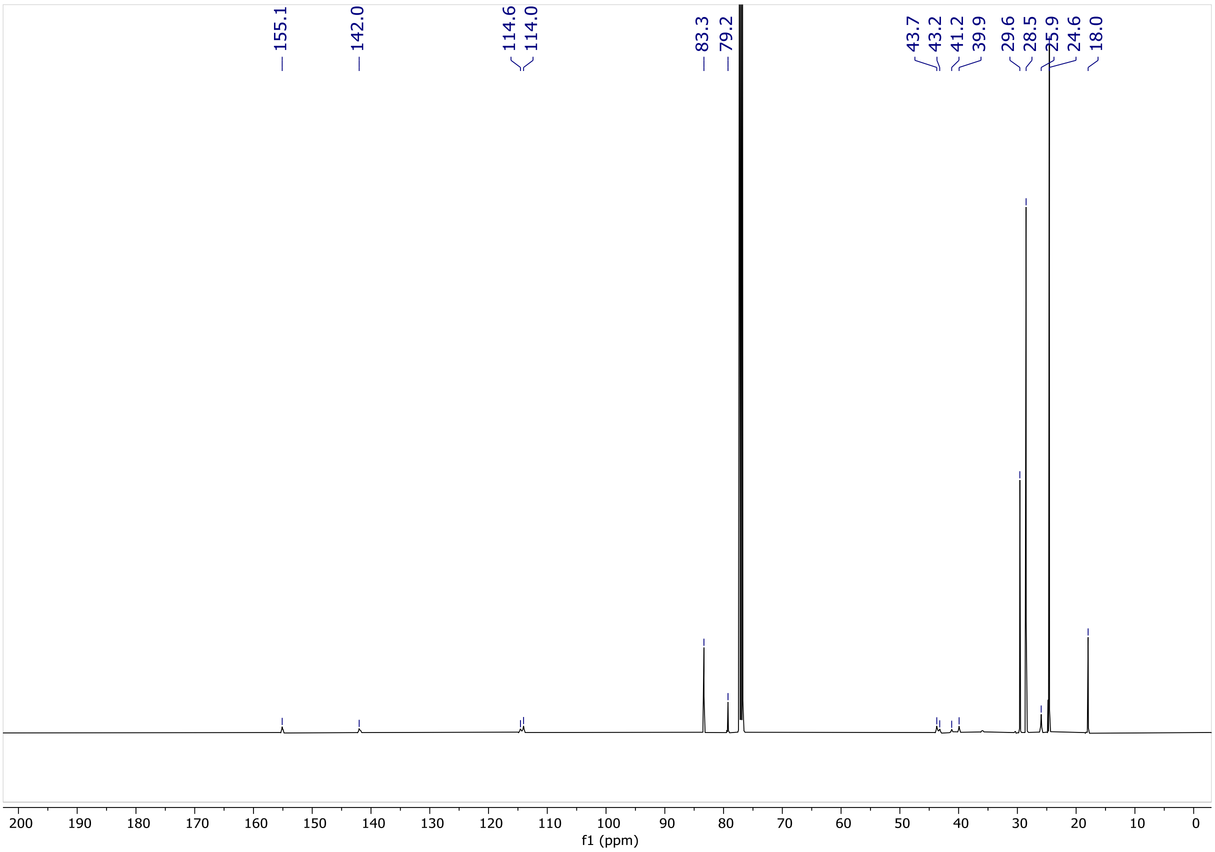

Return to contents

26 2-(1-(benzofuran-2-yl)cyclobutyl)-4,4,5,5-tetramethyl-1,3,2-dioxaborolane

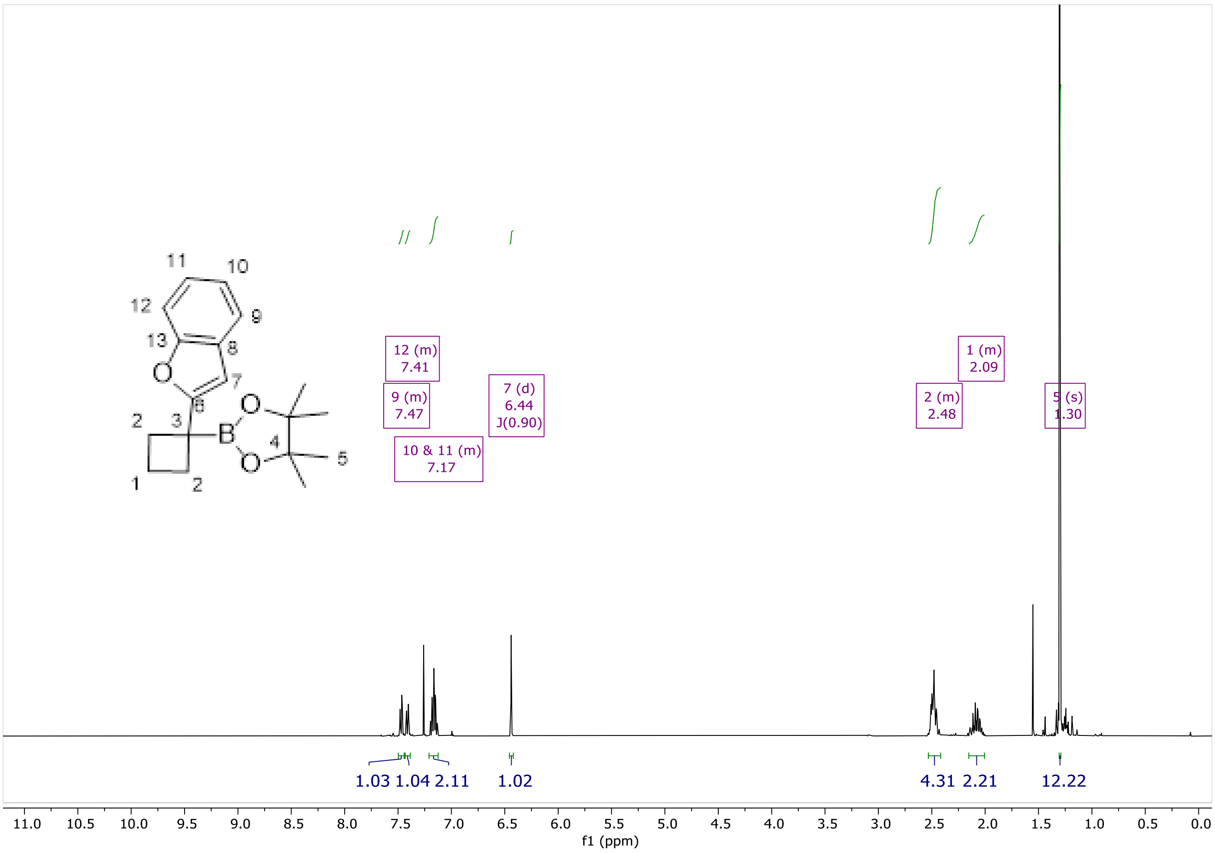

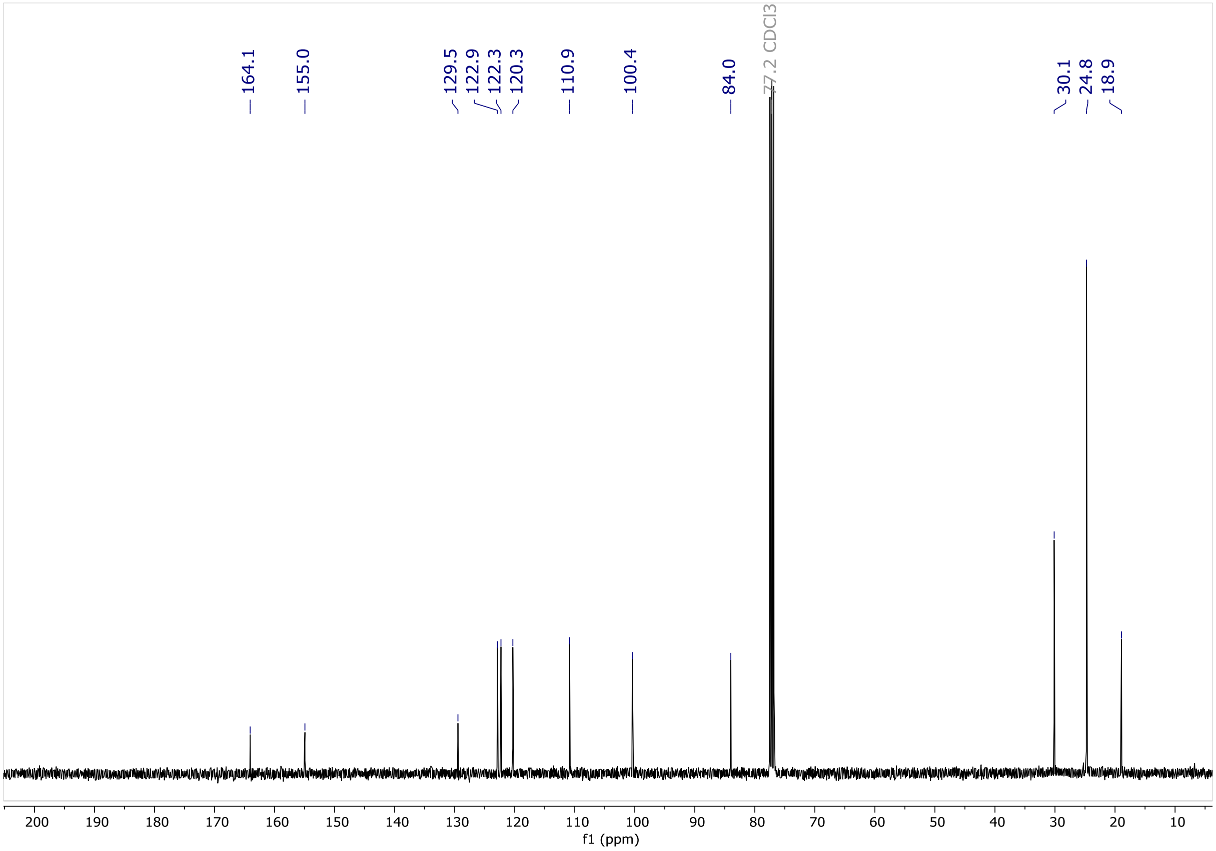

Return to contents

27 *tert*-butyl 5-(1-hydroxycyclobutyl)-1H-indole-1-carboxylate

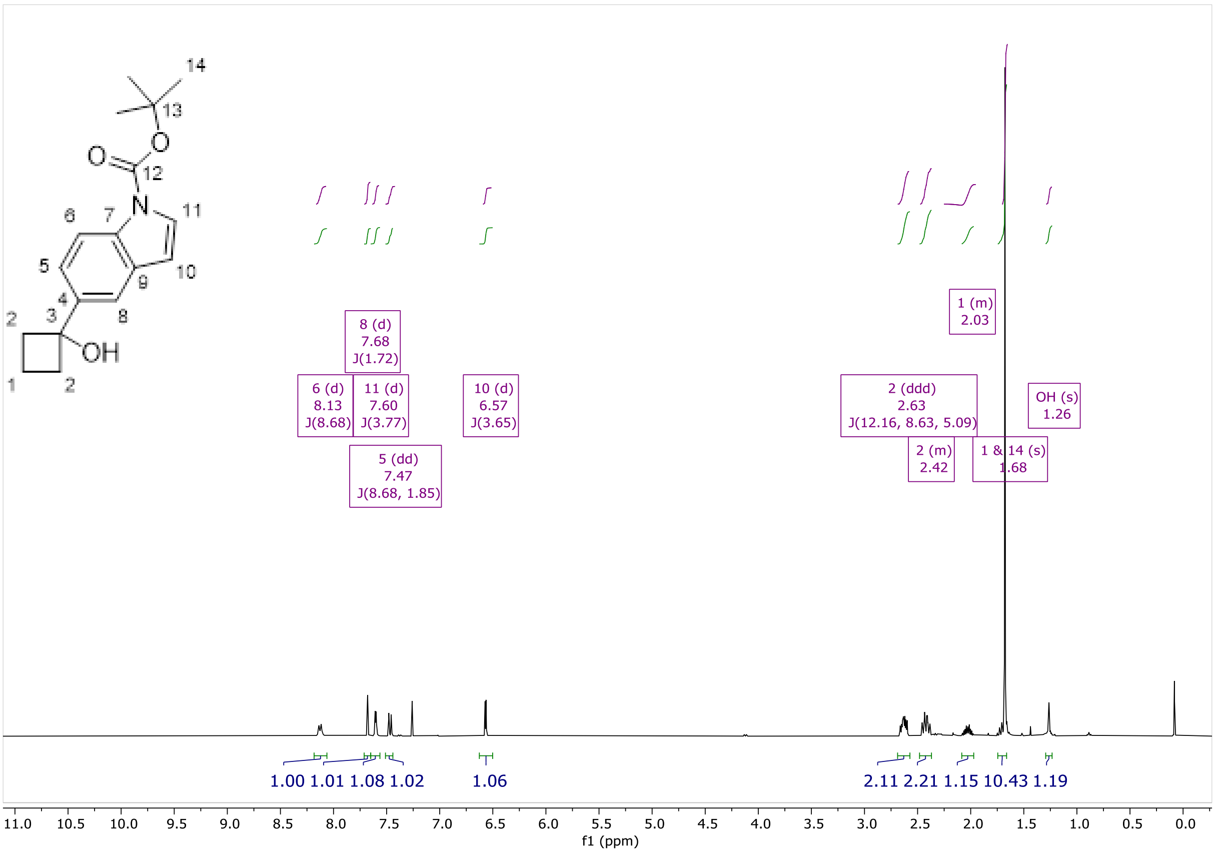

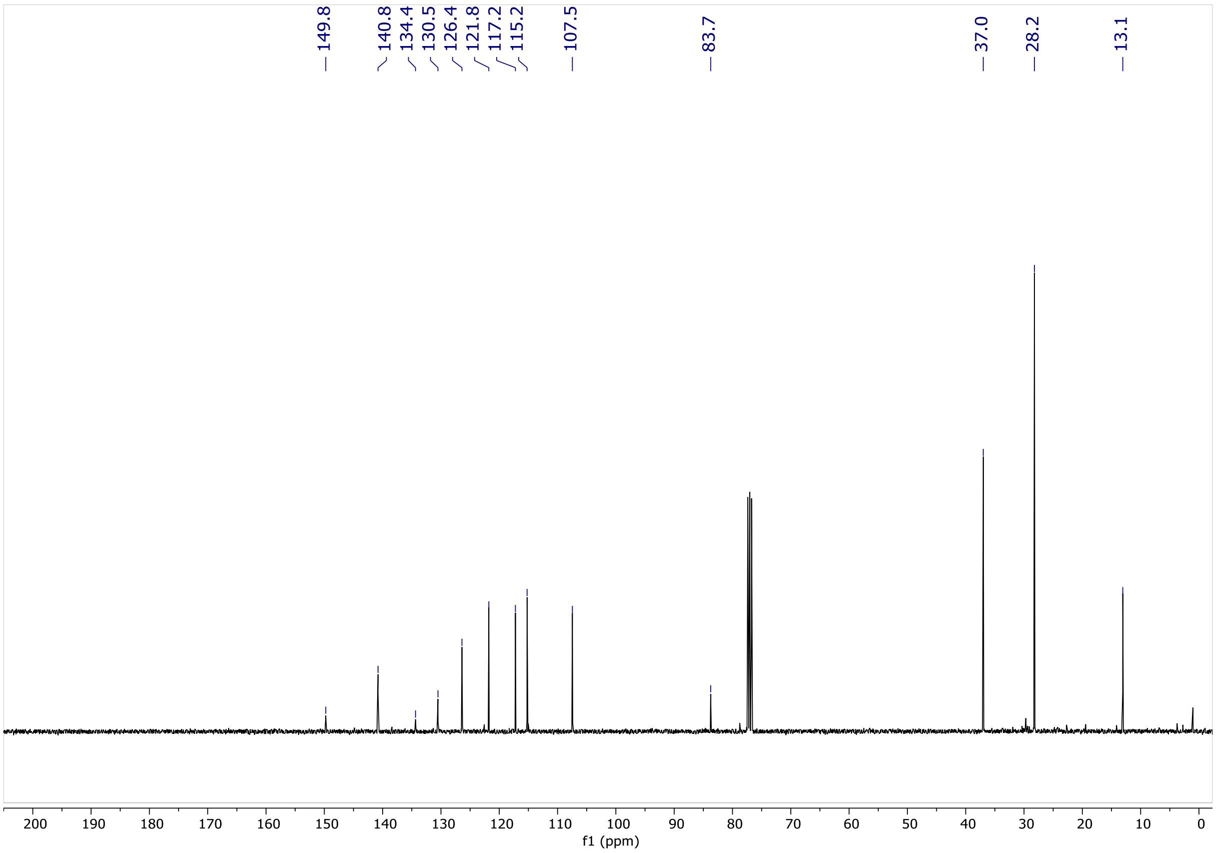

Return to contents

28 (*E*)-1-(4-((tert-butyldimethylsilyl)oxy)but-2-en-2-yl)cyclobutan-1-ol

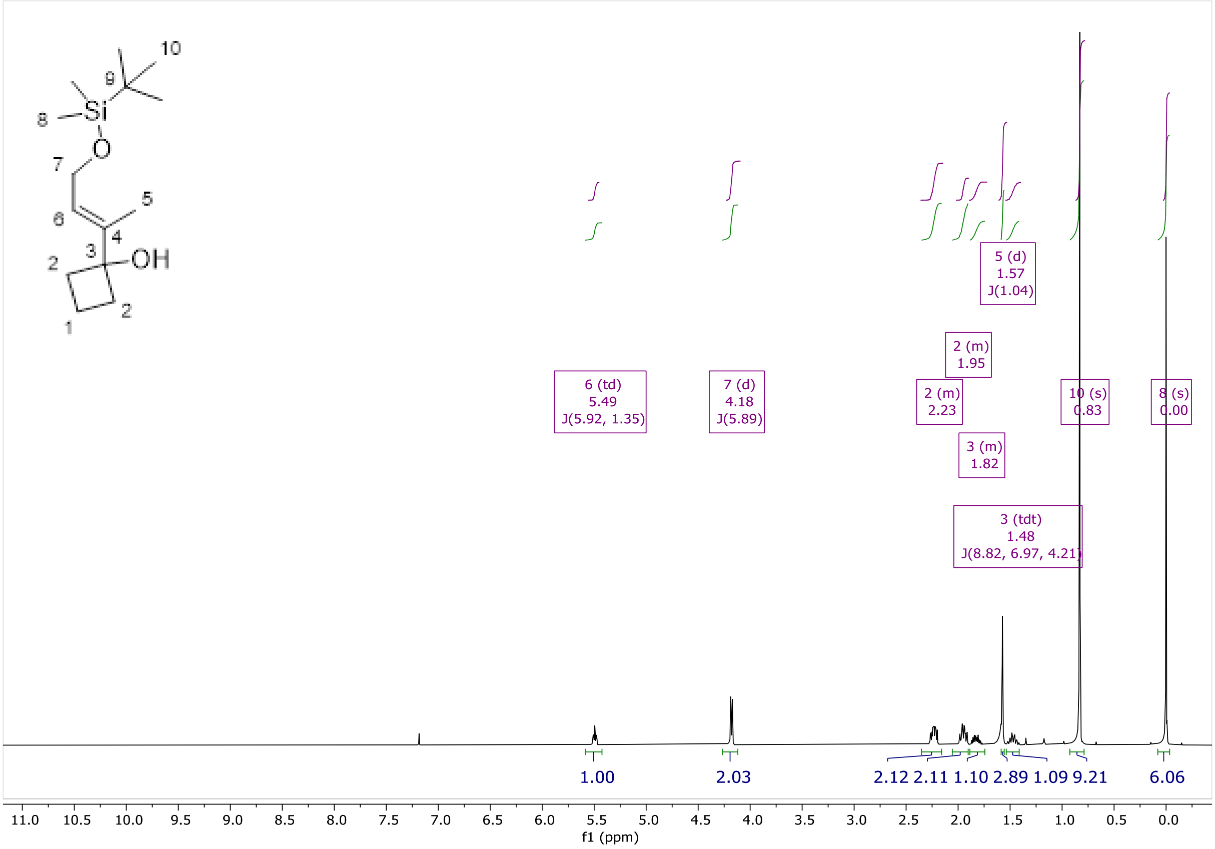

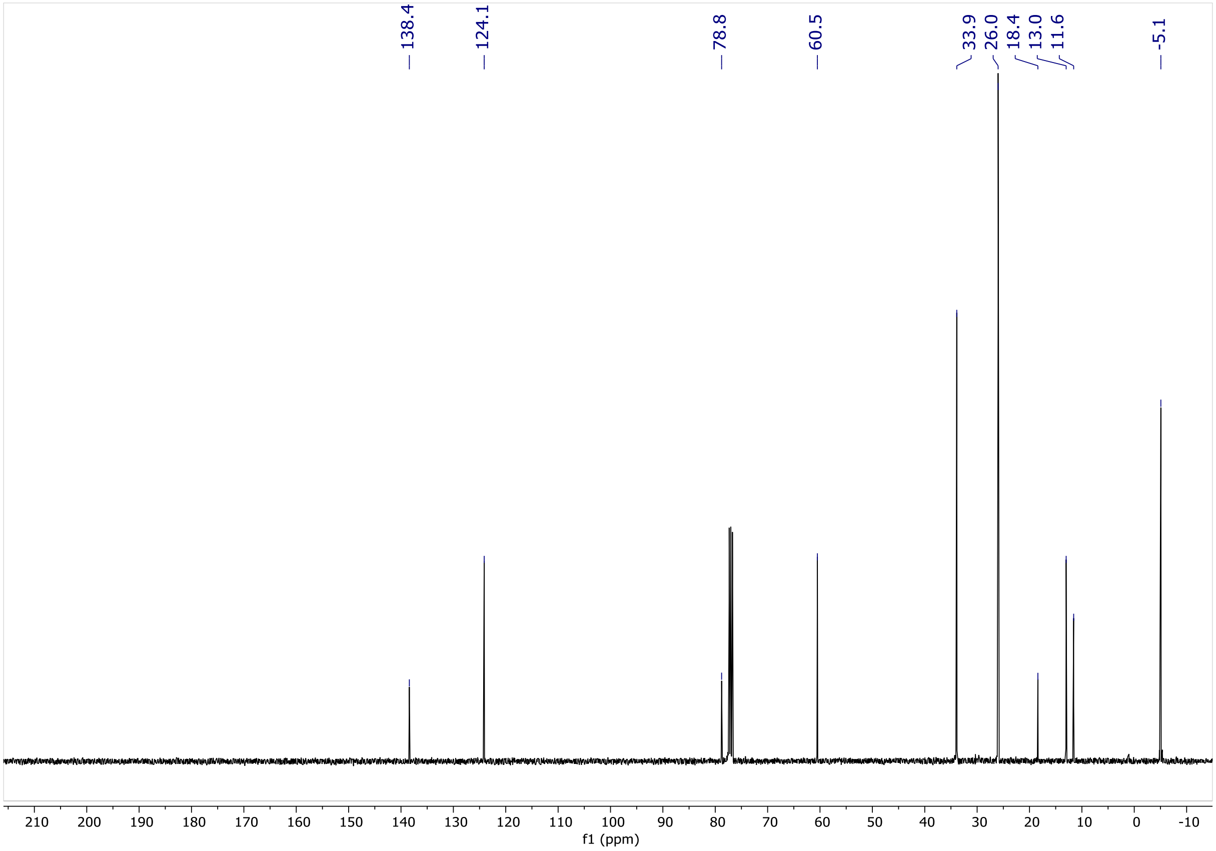

[Return to contents](#)

29 (*E*)-4,4,5,5-tetramethyl-2-(1-styrylcyclobutyl)-1,3,2-dioxaborolane

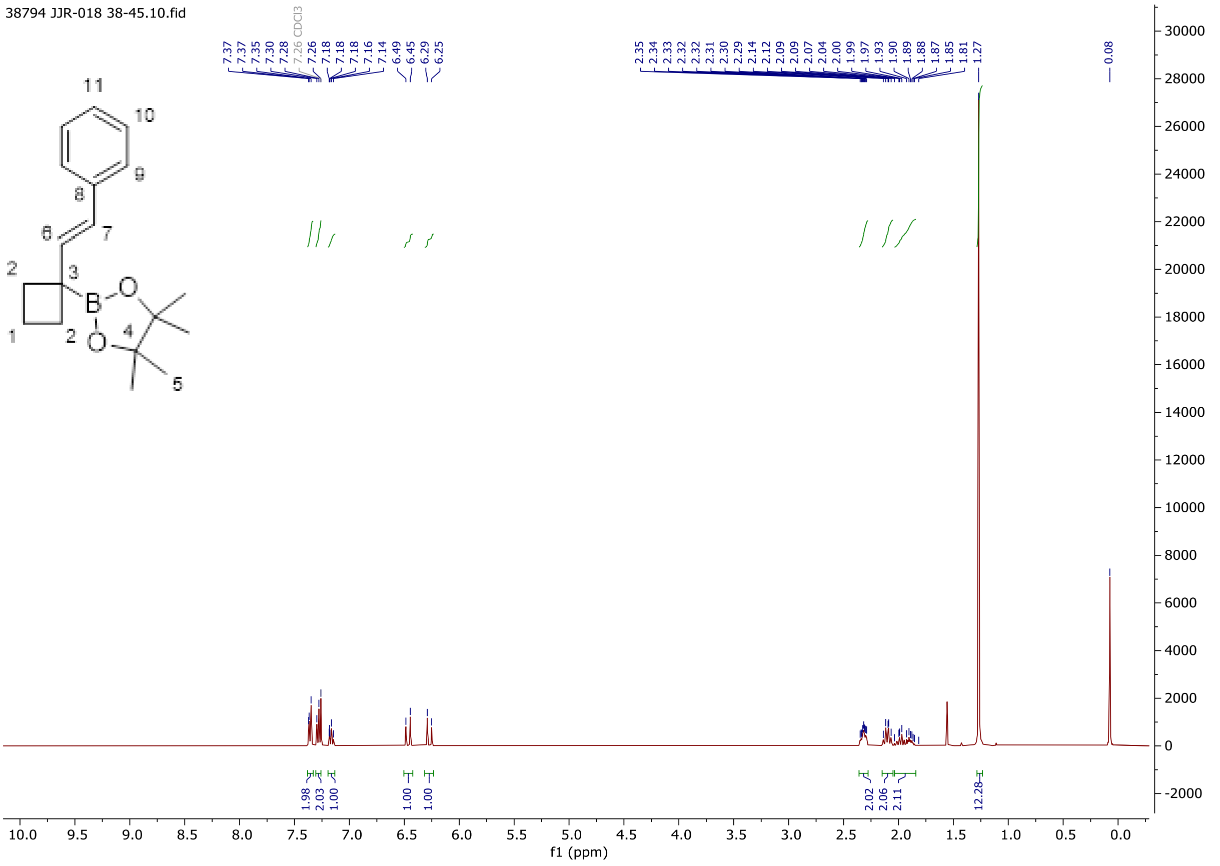

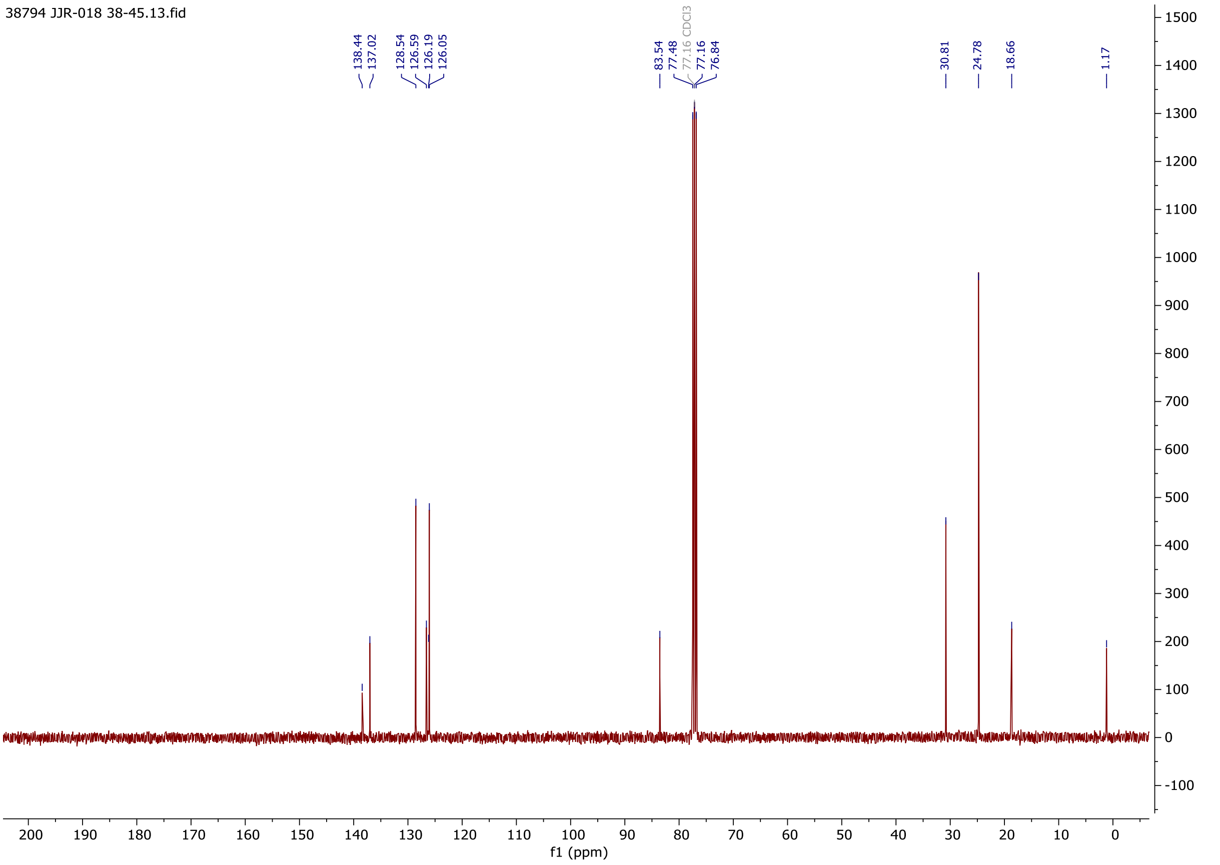

Return to contents

30 (*E*)-4,4,5,5-tetramethyl-2-(1-(3-methylbut-1-en-1-yl)cyclobutyl)-1,3,2-dioxaborolane

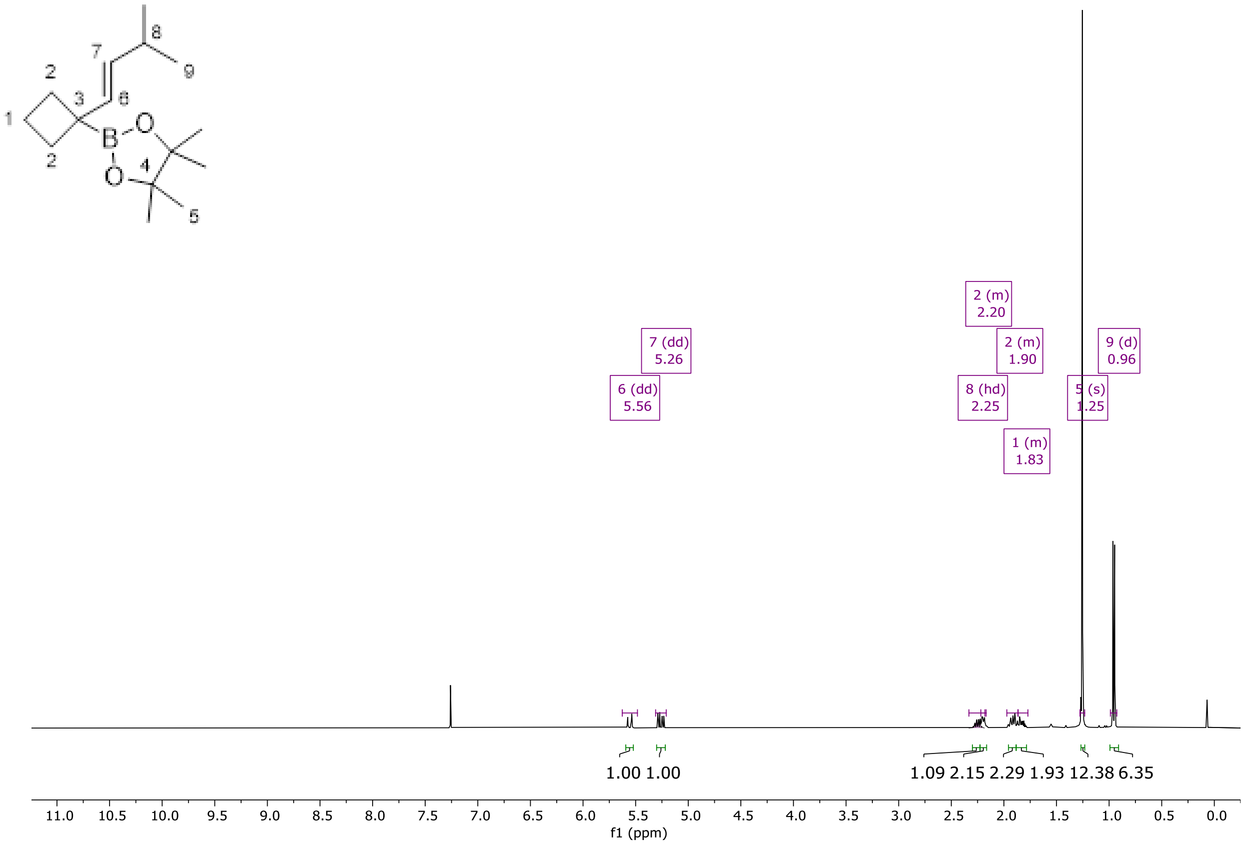

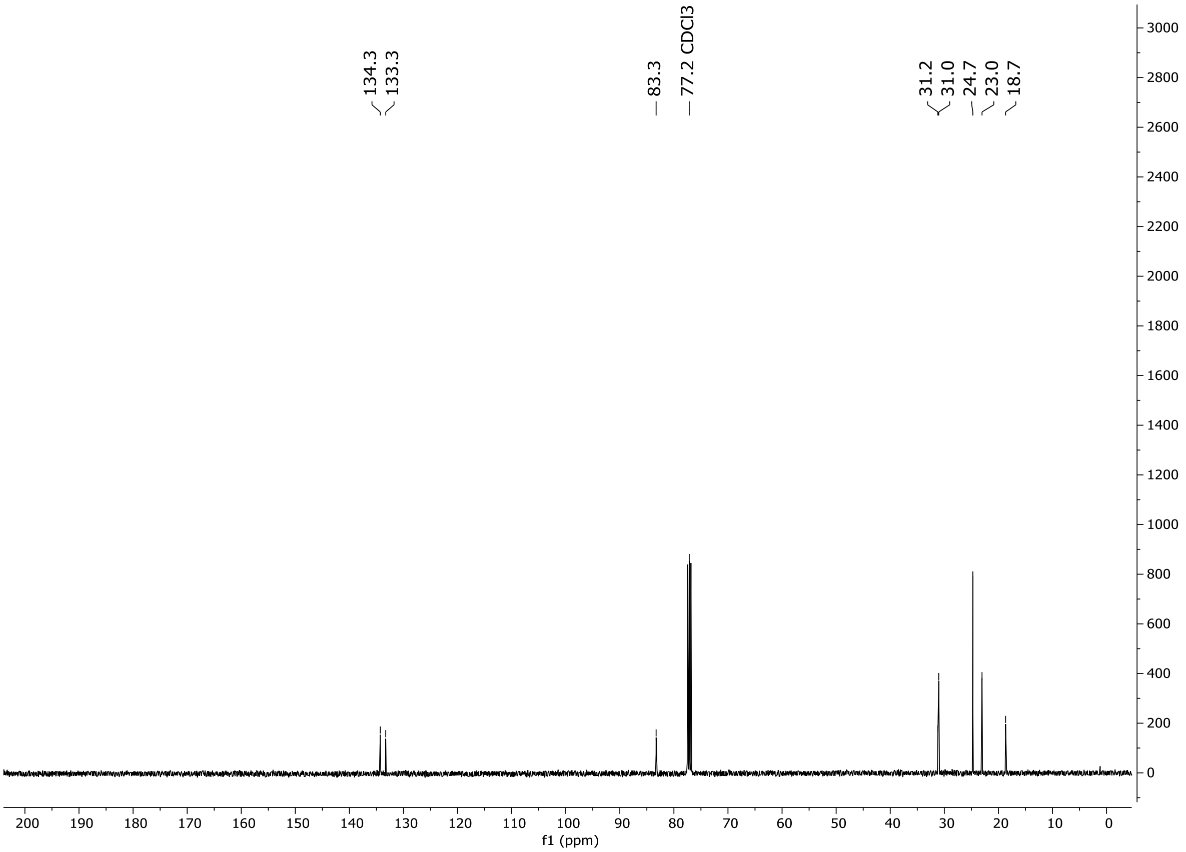

[Return to contents]

31 *tert*-butyl 4-(1-(prop-1-en-2-yl)cyclobutyl)piperidine-1-carboxylate

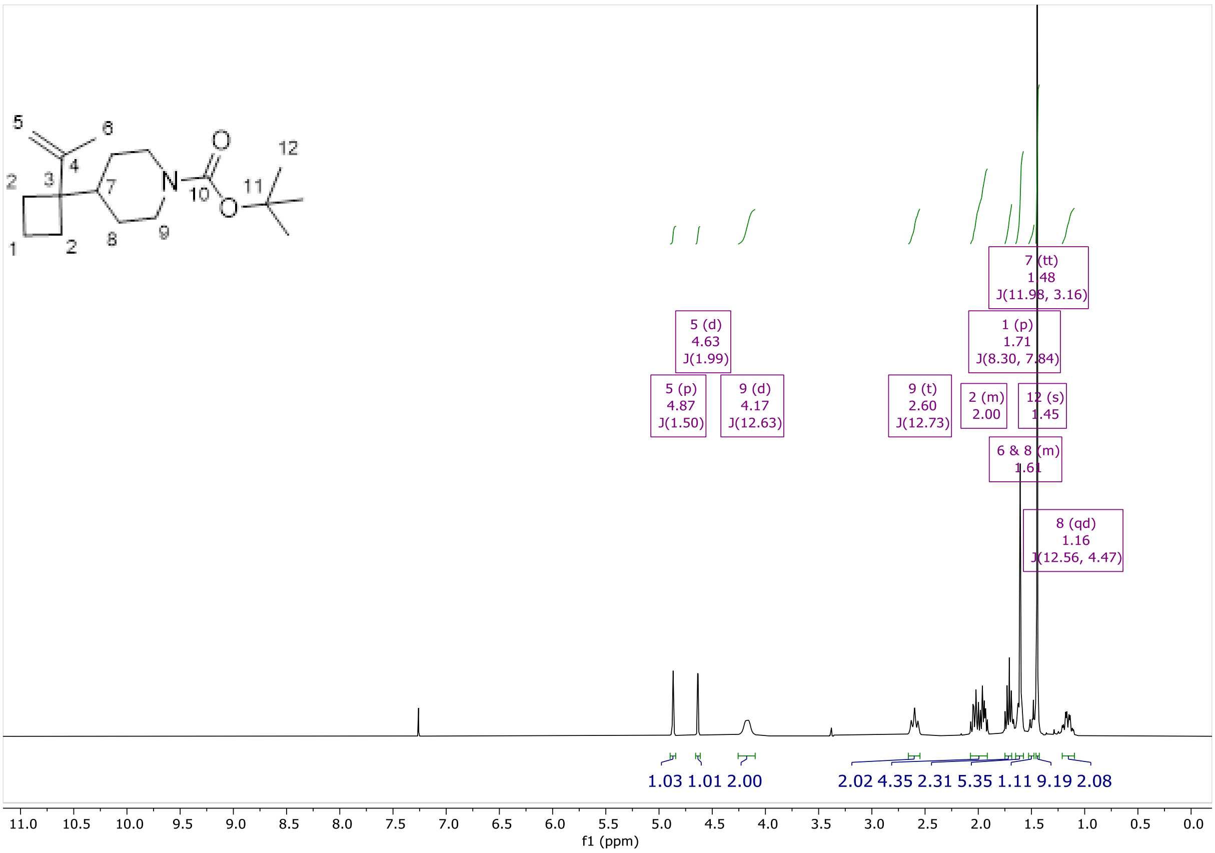

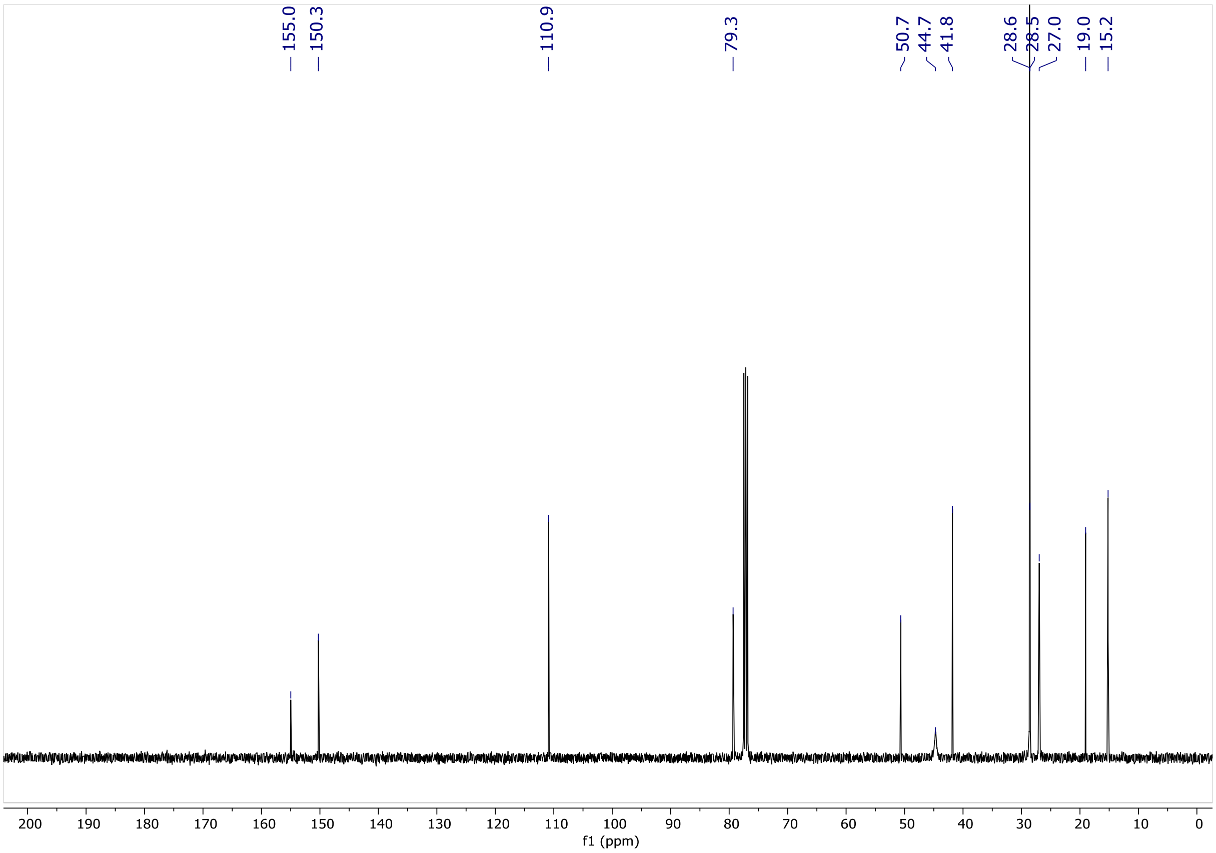

Return to contents

32 *tert*-butyl 4-(1-((4,4,5,5-tetramethyl-1,3,2-dioxaborolan-2-yl)methyl)cyclobutyl)piperidine-1-carboxylate

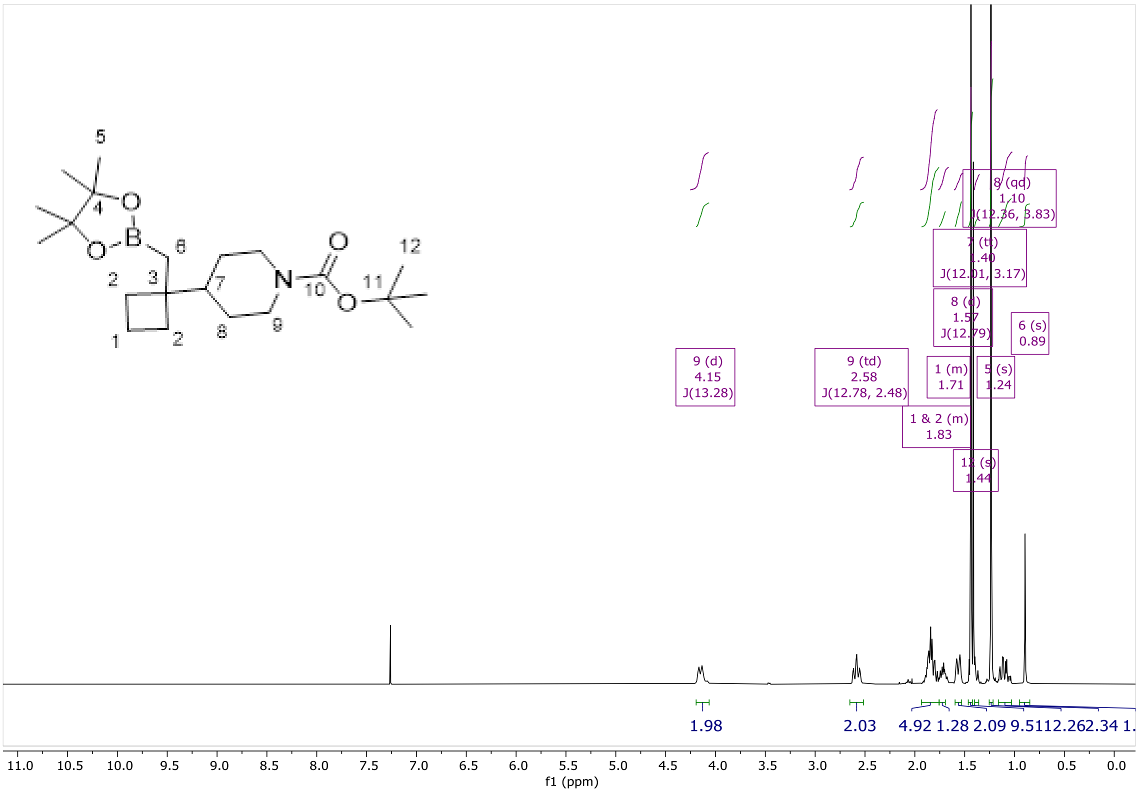

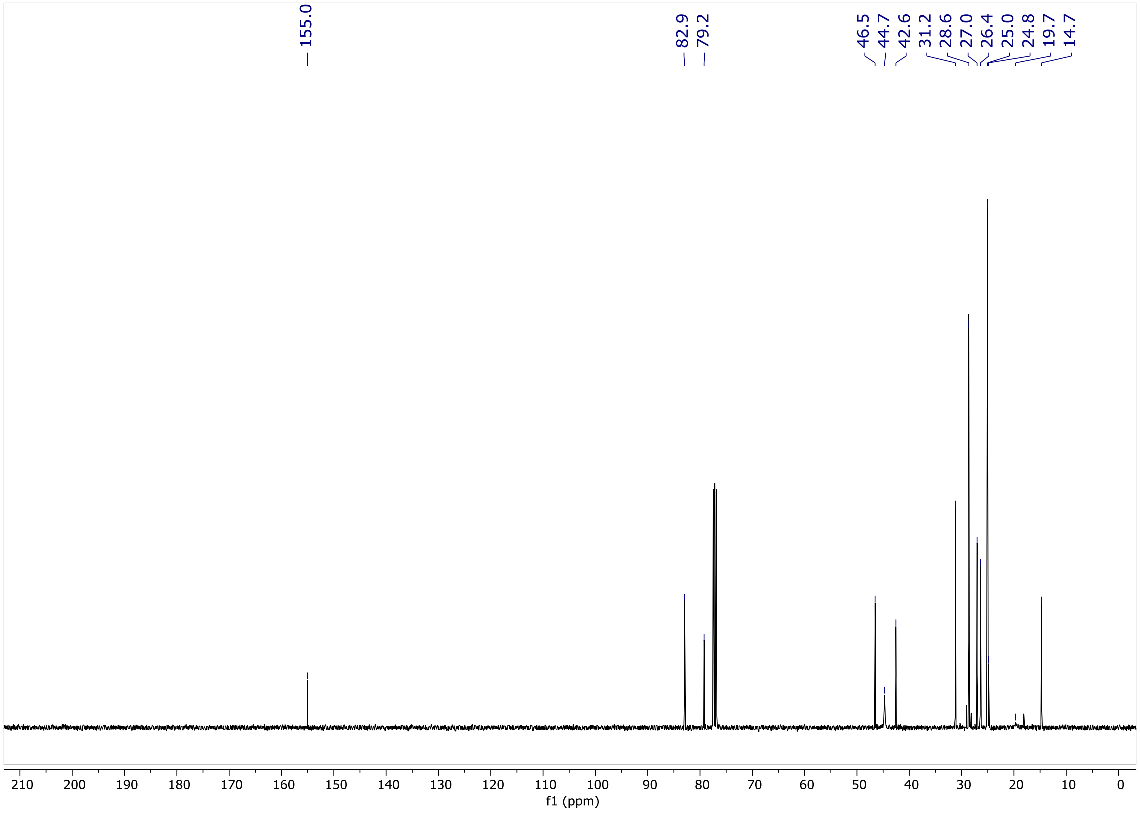

Return to contents

33 tert-butyl 4-(1-((tert-butoxycarbonyl)amino)cyclobutyl)piperidine-1-carboxylate

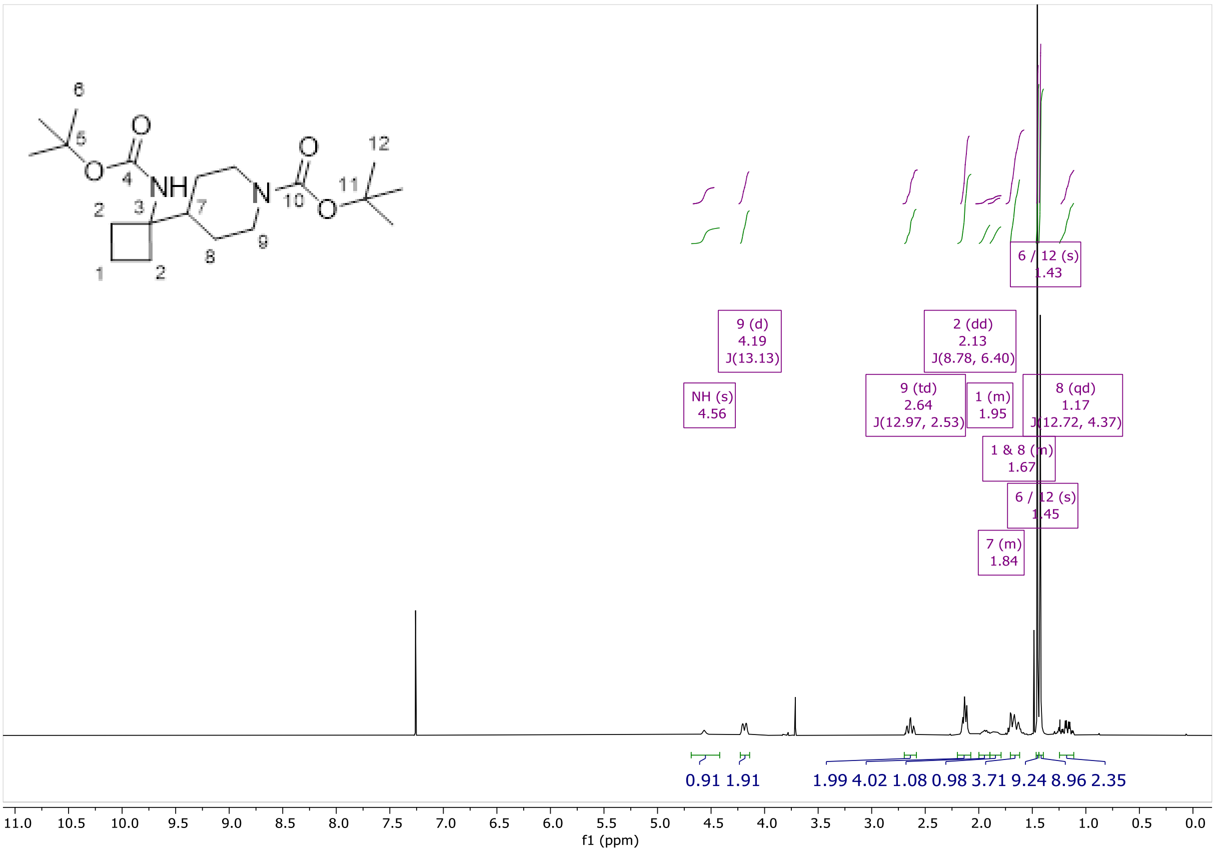

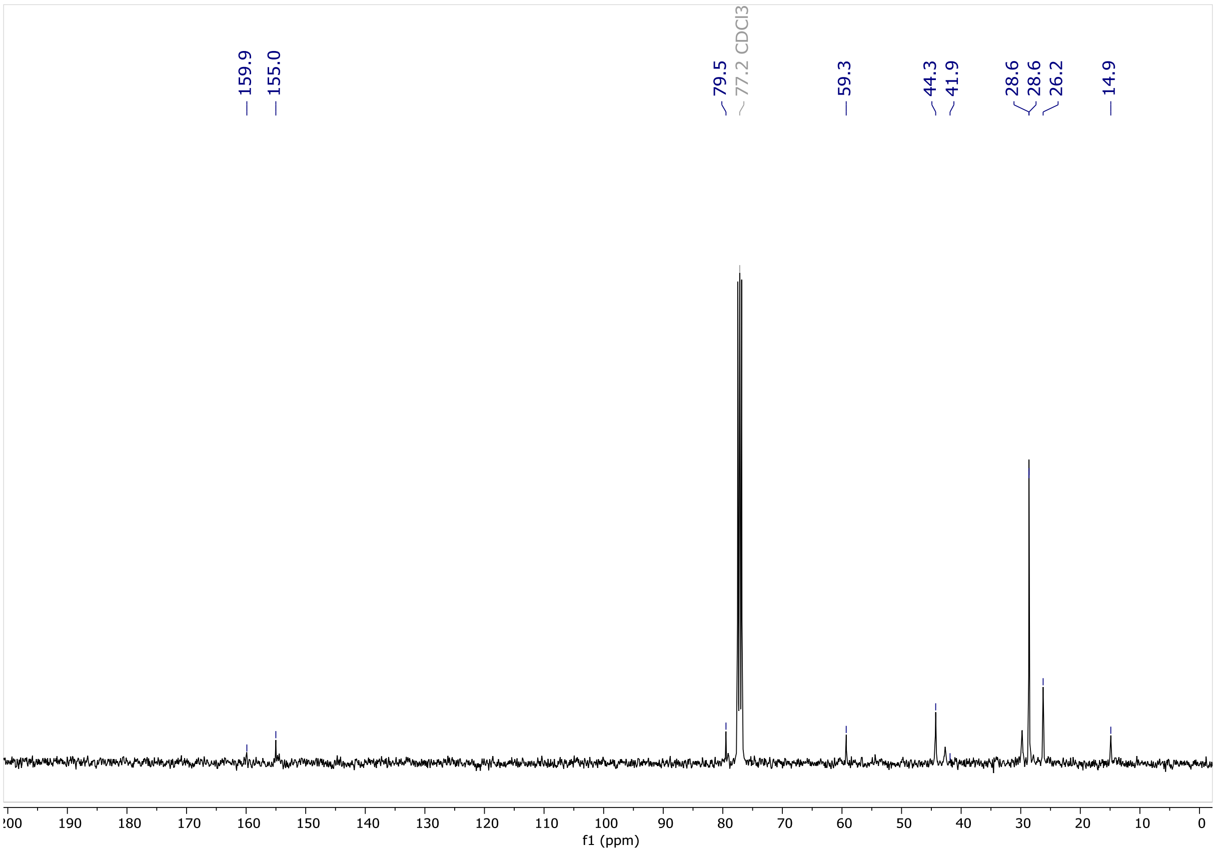

Return to contents

34 *tert*-butyl 4-(1-ethynylcyclobutyl)piperidine-1-carboxylate

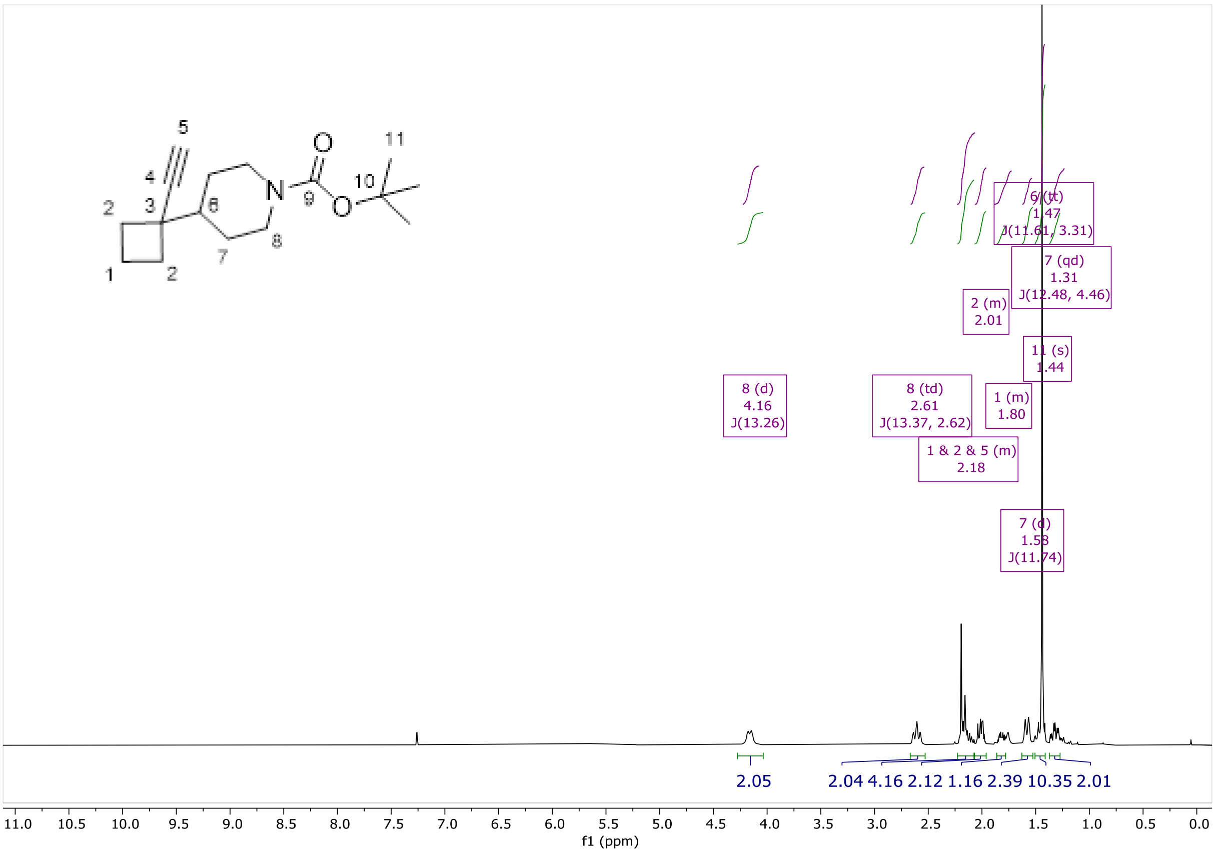

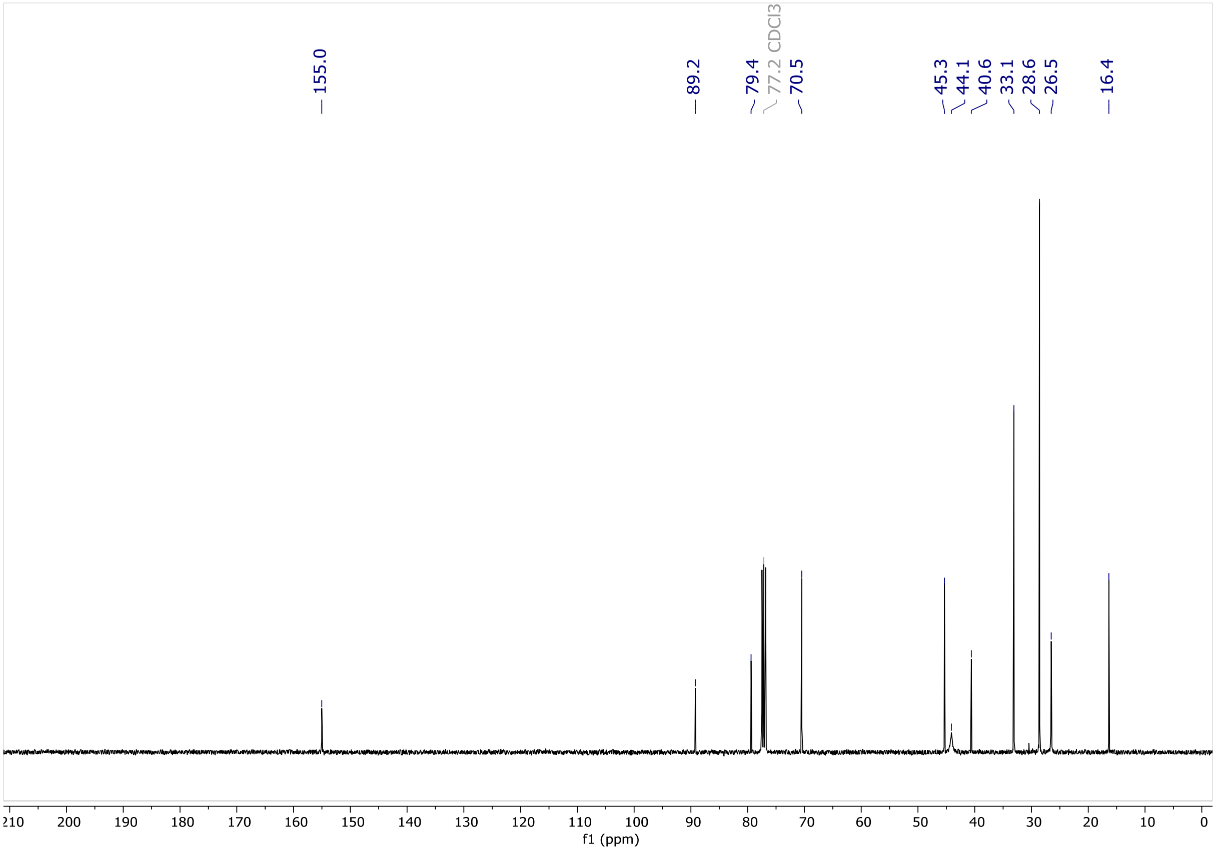

8 References

[1] R. C. Gadwood, M. R. Rubino, S. C. Nagarajan, S. T. Michel, *J. Org. Chem.* **1985**, *50*, 3255–3260.

[2] R. C. Mykura, S. Veth, A. Varela, L. Dewis, J. J. Farndon, E. L. Myers, V. K. Aggarwal, *J. Am. Chem. Soc.* **2018**, *140*, 14677–14686.

[3] D. Stead, G. Carbone, P. O'Brien, K. R. Campos, I. Coldham, A. Sanderson, *J. Am. Chem. Soc.* **2010**, *132*, 7260–7261.

[4] A. P. Pulis, D. J. Blair, E. Torres, V. K. Aggarwal, *J. Am. Chem. Soc.* **2013**, *135*, 16054–16057.

[5] K. Hong, X. Liu, J. P. Morken, *J. Am. Chem. Soc.* **2014**, *136*, 10581–10584.

[6] R. A. Croft, M. A. J. Dubois, A. J. Boddy, C. Denis, A. Lazaridou, A. S. Voisin-Chiret, R. Bureau, C. Choi, J. J. Mousseau, J. A. Bull, *European J. Org. Chem.* **2019**, *2019*, 5385–5395.

[7] S. Balieu, G. E. Hallett, M. Burns, T. Bootwicha, J. Studley, V. K. Aggarwal, *J. Am. Chem. Soc.* **2015**, *137*, 4398–4403.

[8] Y. Wang, A. Noble, E. L. Myers, V. K. Aggarwal, *Angew. Chemie - Int. Ed.* **2016**, *55*, 4270–4274.
